# Astro-Versus Microglia-Enriched Transcriptomes from Aged *Atxn2*-CAG100-Knockin Mice Suggest Underlying Pathology of RNA Processing at Ribosomes, and Possibly at U-Bodies

**DOI:** 10.3390/cells15080699

**Published:** 2026-04-15

**Authors:** Georg Auburger, Arvind Reddy Kandi, Rajkumar Vutukuri, Luis-Enrique Almaguer-Mederos, Suzana Gispert, Nesli-Ece Sen, Jana Key

**Affiliations:** 1 Experimental Neurology, Clinic of Neurology, University Hospital, Goethe University Frankfurt, Heinrich-Hoffmann-Str. 7, 60528 Frankfurt am Main, Germanylalmaguermederos@gmail.com (L.-E.A.-M.); gispert-sanchez@em.uni-frankfurt.de (S.G.);; 2 Institute for Clinical Neuroanatomy, Dr. Senckenberg Anatomy, Fachbereich Medizin, Goethe University Frankfurt, 60528 Frankfurt am Main, Germany; 3 Institute of General Pharmacology and Toxicology, University Hospital, Goethe University Frankfurt, 60528 Frankfurt am Main, Germany; vutukuri@med.uni-frankfurt.de

**Keywords:** Miltenyi MACS adult brain separator, fractionation controls *Aif1*, *Gfap*, *Map2*, *Cldn5*, RNA-seq, STRING enrichment bioinformatics, neurofilament loss, mRNA circularization, translation dynamics, uridine-rich small nuclear RNA, U1 3′ stem-loop structure recognition by SMN

## Abstract

Spinocerebellar Ataxia type 2 (SCA2) and Amyotrophic Lateral Sclerosis type 13 (ALS13) are triggered by polyglutamine expansion in Ataxin-2 (ATXN2). To understand these neurodegenerative disorders at the molecular level, the brains of 10-month-old *Atxn2*-CAG100-knockin mice were analyzed as microglial, astroglial and neuronal fractions via global RNA sequencing. Data were validated by comparison with the spinal cord oligonucleotide microarray profile or filtered by RNA-seq consistency. Here, we show that the mutation causes a massive inflammatory response in microglia and a reciprocal loss of neuronal transcripts in glial fractions, suggesting severe synapse loss. Beyond these general neurodegenerative signs, we identify pathognomonic changes in the machinery for protein translation and RNA splicing. Glial fractions showed upregulation of *Gpnmb* (to 2082%), *Cst7*, *Clec7a*, *Axl*, *Csf1*, *Lgals3*, *Lgals3bp*, *Slc11a1*, and *Usp18* as an unspecific neuroinflammatory signature, versus downregulation of axonal *Nefh* (to <19%), and synaptic *Scn4b*, *Camk2b*, *Rab15*, and *Grin1* mRNAs correlating with circuit disconnection. In all fractions, reductions in *Kif5a*, *Rph3a*, and *Cplx1* were noted versus disease-specific inductions of ribosomal subunits, presumably mirroring the partial loss-of-function of ATXN2 as RNA translation modulator. Selective accumulations of embryonic factors *Rnu1b2* and *Eef1a1* versus downregulation of adult *Eef1a2* specify the mutation impact on splicing and translation elongation. As a potential underpinning of toxic gain-of-function, the proteostasis transcript *Rnf213* appeared increased in astroglial and microglial fractions. These transcriptome data suggest altered ribosomal and spliceosome machinery, with massive microgliosis versus mild astrogliosis, at the core of SCA2 and ALS13.

## 1. Introduction

Our research on molecular mechanisms in neurodegenerative disorders is focused on a rare disease named Spinocerebellar Ataxia type 2 (SCA2), which is caused by a CAG repeat expansion with autosomal dominant inheritance and occurs worldwide [1,2,3,4]. This entity was first described in India [5], and the further definition of its characteristic features was pioneered in a large Cuban founder population [6,7,8,9,10,11,12,13,14,15,16,17,18,19,20,21], as well as in Japan, India, Europe, the USA, and China [22,23,24,25,26,27,28,29,30,31,32,33,34,35,36,37,38,39,40,41,42,43,44,45,46]. Three teams in parallel identified Ataxin-2 (ATXN2) as the responsible disease protein, demonstrating that unstable expansions of its poly-glutamine (polyQ) domain from the normal size of 22 (or 23) to >33 residues represent the causal event that triggers this multi-system atrophy [47,48,49].

Intermediate size expansions in the polyQ domain of ATXN2 do not have the strength to cause monogenic disease, but at lengths perhaps from 27Q, and certainly from 31Q until 33Q render motor neurons of the spinal cord and the brain cortex more vulnerable to manifestation of Amyotrophic Lateral Sclerosis (ALS) at adult ages [50,51,52,53,54]. The pathogenesis involving intermediate-sized polyQ expansions in ATXN2 has been named ALS type 13 (see https://omim.org/entry/601517, last accessed on 25 January 2026).

ATXN2 is a large protein with sizes between 600 and 1300 amino acids, which evolved during the time early eukaryotic life had sustained exposure to oxidative stress from endosymbionts, surrounding them with protective double membranes [55]. Thus, its functions were necessary for all fungi, insects, animals, and plants across evolution, but remain to be understood. All orthologs and paralogs of the protein family across evolution share a characteristic order of an N-terminal Like-Sm (LSm) domain containing five beta-barrels, followed by an LSm-associated domain (LSmAD) with some alpha-helix structures, and at about 3/5 of the protein length, the linear PABP-interacting motif 2 (PAM2) (in plants also known as CID) motif [56,57,58,59], with all of these conserved sequences being surrounded by intrinsically disordered regions (IDRs) of relatively high variability [55,60,61]. The LSm and PAM2 sequences are also found in other protein families, while the LSmAD seems specific to the Ataxin-2 gene family and has the strongest sequence conservation. RNA association is known to occur directly for the LSm domain [62], indirectly somewhere between LSm and LSmAD via the RNA helicase DDX6 [63,64], and indirectly via the poly(A)-binding protein (PABP) for the PAM2 motif [60,65], while the function of LSmAD remains to be understood. Although the interaction with poly(A) tails of mRNAs clearly indicates a role of ATXN2 for coding transcripts, extensive mRNA profiling efforts in diverse organisms were unable to define individual transcripts affected by ATXN2 polyQ expansions with consistency across different tissues. 

Gene duplication events occurred independently when organisms moved from protected freshwater environments to enhanced stress exposures during terrestrial life. In all animals since chordate development (except flying birds), ATXN2 coexists with its duplicated shorter homolog ATXN2L, which shows even stronger expression. LSm/LSmAD/PAM2 deletions in ATXN2L were shown to interfere with mouse embryonic development, similar to the essential role of single-copy Ataxin-2 for the development of nematodes, flies and yeast, while targeted deletion of the N-terminal IDR1 with the polyQ domain of ATXN2 in mice produced only mild phenotypes with adult progressive obesity, high blood fats, insulin resistance, locomotor hyperactivity and subtly altered ribosomal translation efficiency [66,67,68,69,70,71,72,73,74,75,76,77,78,79,80,81,82,83,84,85,86,87]. An independent gene duplication event in the evolution of pioneer terrestrial plants with enhanced stress exposure, such as *Amborella* shrub and *Araucaria* conifer monocots [55], produced Ataxin-2 orthologs that were named CID3 and CID4, but apparently failed to develop distinguishing sequence features, and play enigmatic roles for development [88]. Clearly, a necessity of Ataxin-2 for cellular stress responses and RNA quality control was documented in multiple species across evolution, and its re-localization in stress periods from ribosomes to stress granules for RNA triage (and somewhat to P-bodies where RNA degradation occurs) is its characteristic feature upon microscopic analyses in cells before and during oxidative stress [63,81,83,84,89,90,91,92,93,94]. During normal cellular growth, the experimental manipulation of the Ataxin-2 orthologs, e.g., in flies, and their association with the ribosomal translation machinery was documented. However, *Ataxin-2* mutations also prominently affect the actin cytoskeleton, and this phenotype is not understood at a mechanistic level until today [56,85,95].

Previous PAR-CLIP studies in human HEK293T cells showed that ATXN2 binds directly to AU-rich sequences in the 3′ ends of mRNAs, while luciferase assays in HEK293T and SH-SY5Y cells demonstrated reduced protein expression in the presence of ATXN2-Q31 and ATXN2-Q39 expansions [62]. The analysis of *Drosophila melanogaster* Atx2 mutant brain homogenates confirmed this binding selectivity and showed decreased levels of Atx2-target mRNAs in post-mitotic neurons upon Atx2 knockdown [96,97]. Beyond the role of Ataxin-2 family members for mRNA processing, spurious findings by RNA sequencing technologies in mice, flies, and plants also suggested some dysregulation of microRNA [77,88,98,99], without clear consistencies or a detailed understanding of the mechanism involved.

In our current study, we employed aged mouse brains to generate cell-type-enriched fractions using equipment and reagents from the company Miltenyi Biotech. To have maximal mutation effects, a Q100 domain encoded by a pure CAG100 expansion was substituted for the physiological single Q residue in mouse ATXN2 by targeted knockin technology [100]. This mouse mutant was previously shown to model the spatio-temporal SCA2/ALS13 pattern of neurodegeneration and locomotor behavior deficits, with several pathology aspects in peripheral tissues, in an authentic manner [100,101]. The RNA yields from neuronal fractions turned out to be insufficient for standard RNA-seq analyses, but the highly sensitive MACE-seq enabled quantification of >10,000 mRNAs in each fraction, and the comparison of compound KINvsWT data with individual data from astroglial KINvsWT and microglial KINvsWT permitted stringent data filtering based on consistency criteria. Overall, a prominent impact of ATXN2 polyQ expansion on ribosomal translation was elucidated, with the exploratory data pointing to an impairment of the translation elongation step. Furthermore, an impact of the mutation on RNA processing, involving non-coding small RNAs, was documented, and preliminary insights regarding a plausible mechanism were identified.

## 2. Materials and Methods

### 2.1. Mouse Husbandry

All mouse experiments were approved by regional authorities and performed in compliance with the German Animal Welfare Act, the Council Directive of 24 November 1986 (86/609/EWG), with Annex II and the ETS123 (European Convention for the Protection of Vertebrate Animals). Mice were provided with food and water ad libitum and were housed in a regular 12 h light-dark cycle at the central animal facility (ZFE) of the University Hospital Frankfurt. Heterozygous mating was employed to generate *Atxn2*-CAG100-KIN mice with a C57BL/6 background. The offspring littermates, namely homozygous *Atxn2*-CAG100-knockin (KIN) and wild-type animals of the same sex, were aged in neighboring cages for subsequent comparisons. Of the 10-month-old mice studied by RNA-seq, two mutant-WT pairs were male, and one pair was female.

### 2.2. Genotyping

DNA was isolated from ear punches. In total, 50 µL of alkaline lysis buffer (10 mM NaOH, 0.2 mM disodium EDTA, pH 12) was added to ear punches or tail clips, followed by incubation at 95 °C for 30 min. Samples were cooled down on ice, followed by adding 50 µL of neutralization buffer (40 mM Tris-HCl, pH 5). 1 µL of this extracted DNA was used for polymerase chain reaction (PCR). TaKaRa LA Taq-Polymerase (Takara Bio Inc., #RR002A, Takara Bio USA, San Jose, CA, USA) was used to identify wild-type and *Atxn2*-KIN alleles with primer pair F-NOW1 5′-TGAGTTGACTCCACAGGGAGGTGAGC-3′ and R-NOW1 5′-CCATCTCGCCAGCCCGTAAGATTC-3′. The wild-type allele yields an amplicon size of 793 bp, and the KIN allele yields 948 bp.

### 2.3. Isolation of Cell Types from the Mouse Brain

The subsequent protocol is adapted from Miltenyi adult brain dissociation (Miltenyi, #130-107-677, Miltenyi Biotech, Bergisch Gladbach, Germany): adult neuron isolation, mouse (Miltenyi, #130-126-603); Anti-ACSA2 MicroBead Kit, mouse (Miltenyi, #130-097-679); and CD11b (Microglia) MicroBeads, human and mouse (Miltenyi, #130-093-636)—see [102,103]. All steps were performed with ice-cold buffers unless stated otherwise.

#### 2.3.1. Tissue Dissociation

Mouse brains were isolated, weighed, and transferred to gentleMACS C-tubes (Miltenyi, #130-096-334) containing ice-cold DPBS with Ca^2+^ and Mg^2+^ (DPBS). Each C-tube never exceeded 500 mg of tissue. DPBS was replaced with 1950 µL of enzyme mix 1, as mentioned in the Miltenyi adult brain dissociation kit (Miltenyi #130-107-677). To this, 30 µL of enzyme mix 2 wereadded, and sealed C-tubes were attached to the gentleMACS Octo Dissociator with heaters (Miltenyi, #130-096-427). After 30 min of gentle tissue dissociation at 37 °C, samples were briefly centrifuged in 15 s pulses, never allowing them to cross 300× *g*. All material was allowed to collect at the bottom of the tube, the supernatant was discarded, and the cell pellet was resuspended gently in 10 mL of ice-cold DPBS. The MACS SmartStrainer (Miltenyi, #130-098-462) was rinsed with ice-cold DBPS, and the cell suspension was passed through and collected in a 15 mL tube. Samples were centrifuged at 300× *g* for 10 min at 4 °C. The supernatant was aspirated, the cell pellet was resuspended in ice-cold DBPS, and samples were processed for debris removal. For culturing, brain tissue from 3-day-old pups was dissociated using the Neural Tissue Dissociation Kit (P) (Miltenyi Biotech, #130-029-628), following the manufacturer’s instructions.

#### 2.3.2. Debris Removal

For debris removal, a density gradient approach was used where the cells were resuspended with 3100 µL of ice-cold DBPS, to which 900 µL of debris removal solution was added and gently mixed well. About 4 mL of ice-cold DPBS was added gradually to form two layers. Samples were then centrifuged at 3000× *g* for 10 min at 4 °C. The top two layers were aspirated off, and ice-cold DPBS was added to the bottom layer and mixed well by inverting the tube. This tube was again centrifuged at 1000× *g* for 10 min at 4 °C, and the supernatant was discarded.

#### 2.3.3. Red Blood Cell Removal

To eliminate red blood cells, 1 mL of 1× red blood cell solution (Miltenyi, #130-096-334) was added to the cell pellet and incubated at 4 °C. After 10 min, 5 mL of ice-cold DBPS was added and mixed well. Samples were centrifuged at 4 °C and 300× *g* for 10 min, and the supernatant was discarded.

#### 2.3.4. Magnetic Separation

Before magnetic separation, cell number was determined, and 80 µL of ice-cold DPBS with Ca^2+^ and Mg^2+^ + 0.5% BSA were added to the cell pellet for a total number of cells in the range of 5 × 10^6^–4 × 10^7^. Microglia, astrocytes and neurons were isolated sequentially from a single mouse brain. (i) In total, 20 µL of CD11b (Microglia) MicroBeads (Miltenyi #130-093-636) was added to the cell suspension and incubated at 2–8 °C for 10 min. The LS columns (Miltenyi, #130-042-401) were rinsed with 2 mL of ice-cold DPBS with Ca^2+^ and Mg^2+^ + 0.5% BSA, and flow-through was discarded. A final volume of 500 µL of cell suspension was allowed to flow through the LS columns, and the flow-through was collected, which contained unlabeled cells. The LS column was washed 3 times with ice-cold DPBS with Ca^2+^ and Mg^2+^ + 0.5% BSA. The microglia fraction was eluted into a new tube by firmly pushing the plunger onto the LS column containing 1 mL of ice-cold DPBS with Ca^2+^ and Mg^2+^ + 0.5% BSA. This process was repeated once again to enrich purity. (ii) The unlabeled cell fraction was centrifuged at 4 °C at 300× *g* for 10 min, and the cell pellet was resuspended in 80 µL of ice-cold DPBS with Ca^2+^ and Mg^2+^ + 0.5% BSA. In total, 10 µL of FcR blocking reagent (Miltenyi #130-115-390) was added to the cell suspension and incubated at 2–8 °C. After 10 min, Anti-ACSA-2 MicroBeads (Miltenyi #130-115-390) were added and again incubated at 2–8 °C for 15 min. Magnetic separation was performed similarly to the microglia fraction. (iii) The unlabeled cell fraction after astrocyte isolation was centrifuged at 4 °C at 300× *g* for 10 min, and the cell pellet was resuspended in 80 µL of ice-cold DPBS with Ca^2+^ and Mg^2+^ + 0.5% BSA. In total, 20 µL of non-neuronal cell biotin-antibody cocktail (Miltenyi #130-115-390) was added to the cell suspension and incubated at 2–8 °C. After 5 min, 20 µL of anti-biotin MicroBeads (Miltenyi #130-115-390) and 10 µL of CD31 MicroBeads (Miltenyi #130-097-418) were added to the cell suspension and incubated at 2–8 °C for 12 min. A final volume of 500 µL of cell mixture was allowed to flow through the LS columns. The columns were washed twice with 1 mL of ice-cold DPBS with Ca^2+^ and Mg^2+^ + 0.5% BSA. The flow-through contained neuronal cells.

#### 2.3.5. Culture of Primary Cells

The 24-well culture dishes were coated with 0.01 mg/mL poly-L-Lysine (PDL, Merck Millipore, Burlington, MA, USA) for 3 h at 37 °C, followed by washing once with sterile H_2_O. The microglia were cultured in DMEM/F12 (Gibco, Waltham, MA, USA) supplemented with 10% FBS (Gibco), 1% non-essential amino acids solution (Gibco), and 1× Pen-strep (Gibco). Astrocytes were cultured in MACS^®^ Neuro Medium (Miltenyi # 130-117-031) supplemented with 0.2% AstroMACS Supplement, 2% MACS NeuroBrew-21, 0.25% L-glutamine (0.5 mM) (Gibco) and 1× Pen-strep (Gibco). Neurons were cultured in Neuro Basal Media (Gibco), 2% B27 supplement (Thermo), 1% L-glutamine, 0.2% FGF (PeproTech), 0.2% PDGF (Proteintech) and 1× Penstrep (Gibco).

#### 2.3.6. RNA Extraction, Library Preparation, RNA-Seq, Differential Expression Analysis, and GSEA

Total RNA was extracted from all the isolated cell fractions using TRIzol (Sigma Aldrich, St. Louis, MO, USA) as per the manufacturer’s protocol. RNA quantification and quality scores were measured using a LabChipDX equipment (Revvity Inc., Waltham, MA, USA), and were adequate for subsequent sequencing in all fractions. The company GenXPro (Frankfurt am Main, Germany) performed the RNA sequencing. Sequencing libraries were prepared using the MACE-seq protocol (GenXPro GmbH, Frankfurt am Main, Germany). While standard RNA-seq requires between 100 ng and 1 µg of high-quality total RNA, MACE-seq focuses exclusively on the highly informative 3′-untranslated region, sequences short fragments of 50–800 bp, and generates reliable expression profiles even from single-cell amounts such as 0.03 ng of total RNA, being suitable even for degraded RNA from archived fixed and embedded tissue after a dozen years of storage, or for laser capture microdissected tissue, or for exosomal cargo [104,105,106,107,108,109]. Ribosomal RNA was depleted using the Qiagen FastSelect system (Qiagen Inc., Hilden, Germany), followed by polyadenylation of RNA molecules with *E. coli* poly(A) polymerase (New England Biolabs, Ipswich, MA, USA). Subsequent NGS library preparation, including the incorporation of “TrueQuant” unique molecular identifiers with dual indexing as molecular barcodes to eliminate PCR-induced bias and allow for precise quantification of mRNAs, was performed using the MACE-Seq kit (GenXPro GmbH, Frankfurt, Germany) according to the manufacturer’s instructions (see https://genxpro.net/sequencing/transcriptome/mace-massive-analysis-of-cdna-ends/, last accessed on 21 March 2026). Libraries were sequenced on an Illumina NextSeq 500 platform with 1 × 76 bps (Illumina Inc., San Diego, CA, USA). The differential expression analysis was performed with DESeq2 version 1.38, using the Benjamini–Hochberg correction for multiple testing, and employing cutoffs with an FDR-adjusted *p*-value lesser or equal to 0.05 and an absolute log2FC greater than or equal to 1.0 to establish significance. Further details on the panRNA-seq analysis procedures, quality control assessments, and gene set enrichment analyses (GSEA) can be found in Supplementary Materials S1 and S2, which were generated by the company GenXPro.

### 2.4. Reverse Transcription-Quantitative Polymerase Chain Reaction (RT-qPCRs)

TRIzol reagent (Sigma, #102543131) was used to isolate total RNA as per the manufacturer’s instructions. To convert RNA to cDNA, we used SuperScript™ IV VILO™ Master Mix with ezDNase™ Enzyme (Invitrogen #11766050, Waltham, MA, USA) with about 1 µg of total RNA as a template for all samples. After cDNA synthesis, the samples were diluted to a 1:10 ratio with ultrapure water. Each sample had biological replicates and technical duplicates. qPCR was performed using 2× FastStart Universal Probe Master (Roche, Basel, Switzerland, #4913957001) with TaqMan Gene Expression Assays (Applied Biosystems, Waltham, MA, USA) in StepOnePlus Real-Time PCR System (Applied Biosystems). The 2^−ΔΔCT^ method [110] was used for data analysis, from which expression values were averaged across biological/technical replicates per condition. The following Taqman assays were used: *Aif1*: Mm00520165_m1, *Atxn2*: Mm01199894_m1, *Cd31*: Mm01242576_m1, *Cldn5*: Mm00727012_s1, *Dlg4*: Mm00492193_m1 *Gfap*: Mm01253033_m1, *Kcnj8*: Mm00434620_m1, *Map2*: Mm00485231_m1, *Olig2*: Mm01210556_m1, *Tmem119*: Mm00525305_m1, and *Tbp*: Mm00446973_m1.

### 2.5. Immunohistochemistry

The respective cell types were cultured for 5 days post-isolation before immunocytochemical analysis. Cells were washed once with 1x phosphate-buffered saline (PBS) and fixed with 4% paraformaldehyde (PFA) in 1x PBS for 5 min at room temperature. Following fixation, cells were permeabilized with 0.1% Triton X-100 in 1× PBS for 2 min and subsequently blocked with Roti^®^-Block solution (Carl Roth, Karlsruhe, Germany) for 30 min at room temperature. For microglia and astrocyte staining, cells were incubated overnight at 4 °C in Roti^®^-Block containing the following primary antibodies: anti-IBA1 (Proteintech, CL647-81728, 1:200) or anti-GFAP (Proteintech, Rosemont, IL, USA, CL488-60190, 1:200), respectively. After primary antibody incubation, cells were washed three times with 1× PBS and counterstained with DAPI (Sigma-Aldrich, 1 mg/mL, D9542) to label nuclei. For neuronal staining, cells were incubated overnight at 4 °C with anti-MAP2 primary antibody (Proteintech, 17490-1-AP, 1:200) diluted in Roti^®^-Block. After washing with 1× PBS, cells were incubated with Donkey anti-Rabbit IgG (H+L) Cross-Adsorbed Secondary Antibody, DyLight™ 550 (Invitrogen, SA5-10039, 1:1000) for 1 h at room temperature. Following final washes with 1× PBS, all samples were mounted using DAKO mounting medium (Agilent, Santa Clara, CA, USA, #S3023) prior to imaging. Cell imaging was performed using Keyence, Frankfurt, Germany, BZ-X series, and ImageJ software (version 1.54) was used to merge images.

### 2.6. Quantification and Statistical Analysis

#### 2.6.1. Statistics

Statistical analysis was performed using GraphPad Prism (Boston, MA, USA, version 8). Volcano Plots were generated with GraphPad Prism (version 11). Bar graphs represent mean ± SEM and *p*-values from Welch’s *t*-test for spinal cord tissue. For cell culture experiments, bar graphs represent mean ± SEM, and 2-way ANOVA with multiple comparisons was used to determine *p*-values (T 0.05 < *p* < 0.1; * *p* < 0.05; ** *p* < 0.01; *** *p* < 0.001; **** *p* < 0.0001).

#### 2.6.2. STRING Pathway Interaction and Enrichment Analysis

The web server https://string-db.org/ was used from 2024 to 2026 with version 12.0, with the last access on 15 January 2026.

## 3. Results

To understand and characterize the diseases SCA2 and ALS13 in different cell populations of the central nervous system, the *Atxn2*-CAG100-KIN model was used to isolate microglia, astrocytes, and neuron fractions from the whole mouse brain. This study aimed to obtain insights into the contribution of various cell types in pathology and to enable insights into aberrant RNA processing, via the RNA-seq approach rather than previously employed oligonucleotide microarrays, and to assess small RNA species (e.g., tRNAs and microRNAs).

### 3.1. Isolation of Murine Brain Cell Types Using MACS Technology

**Figure 1:**
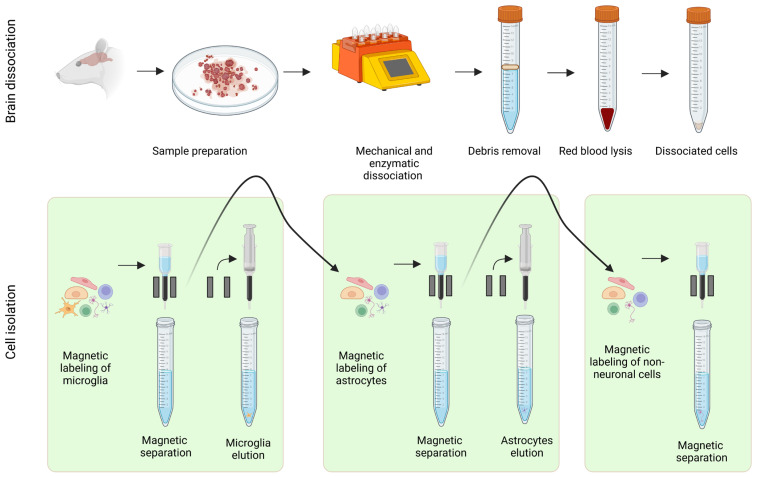
Schematic workflow for adult brain dissociation and sequential isolation of cell types (microglia, astrocytes, and neurons). Tissue is harvested rapidly, placed in ice-cold DPBS with Ca^2+^ and Mg^2+^, and cut into small pieces, then transferred to C-tubes and dissociated by a combination of enzymatic buffer solution containing papain with gentle mechanical dissociation, using a gentleMACS Octo Dissociator with heaters. Gradient centrifugation forms a compact ring containing debris (dead cells and myelin). After debris removal, red blood cells are lysed, and the dissociated cells are magnetically labeled with anti-CD11b MicroBeads against microglia. The labeled cells are passed through LS columns twice and placed on a magnetic stand. CD11b-positively selected cells remain in the column and are eluted with a plunger into a fresh tube. The negative flow-through is processed to isolate astrocytes labeled with anti-ACSA2 MicroBeads, and the process is repeated. For neuron isolation, the negative flow-through from astrocyte extraction is labeled with a non-neuronal biotin cocktail and anti-biotin MicroBeads, and the subsequent negative flow-through contains the neuron population. The arrows show the workflow handling of the cell suspensions. Image created in BioRender, Reddy, A. (2026) https://BioRender.com/mj9rqid, last accessed on 2 March 2026 and adapted from [111].

The workflow of the cell isolations is shown in Figure 1. Overall, brain dissociation followed the procedure described by [111] to isolate several cell types from a single mouse brain. Brains were dissected and collected in ice-cold DPBS with Ca^2+^ and Mg^2+^, then chopped into eight pieces and placed in gentleMACS C-tubes. Mechanical dissociation was performed with the Adult Brain Dissociation Kit (mouse and rat) and the 37C_ABDK_01 program of the gentleMACS Octo Dissociator with Heaters.

After dissociation, debris removal, and red blood cell lysis, cells were magnetically labeled and isolated using MACS technology as follows: (i) microglia with anti-CD11b MicroBeads (positive selection), (ii) astrocytes with anti-ACSA-2 MicroBeads (positive selection), and (iii) neurons with a non-neuronal cell biotin-antibody cocktail followed by anti-biotin MicroBeads (negative selection).

Initial trials showed high endothelial cell contamination in the neuron population. To resolve this, anti-CD31b MicroBeads were added alongside anti-biotin MicroBeads before magnetic separation, significantly improving neuron purity.

### 3.2. The Impact of the Atxn2-CAG100-KIN Mutation on the Brain and Its Cell Types

After establishing and validating the protocol by RT-qPCR (Supplementary Figure S1) and immunocytochemistry, we isolated microglia, astrocytes, and neurons from three wild-type (WT) and three approximately 10-month-old *Atxn2*-CAG100-KIN mutant-WT pairs to identify dysregulated molecules in different cell types due to SCA2 pathology.

**Figure 2:**
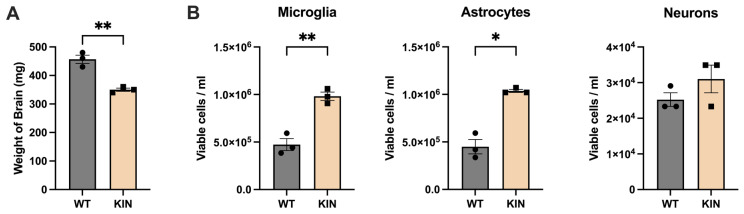
Analysis of the brain and its cell types from *Atxn2*-CAG100-KIN mice. (**A**) Statistical evaluation of brain weights from WT and *Atxn2*-CAG100-KIN mice at ages of ~10 months (*n* = 3). (**B**) Viable cell counts of microglia, astrocytes, and neurons from WT and *Atxn2*-CAG100-KIN mice (*n* = 3). Data are mean ± SEM, Student’s *t*-test with Welch’s correction, *p* < 0.05 *, *p* < 0.01 **, *p* < 0.001.

At the age of 10 months, *Atxn2*-CAG100-KIN mutant brains showed significant weight reduction, averaging 350 mg compared to the wild-type average of 450 mg, consistent with previous findings regarding the severe cerebellar and moderate cortical atrophy [100] (Figure 2A). Post-isolated individual cells had 92% viable single-cell suspension. An average of 444,000 microglia, 419,667 astrocytes, and 23,666 neurons was obtained. *Atxn2*-CAG100-KIN mutants had significantly higher numbers of microglia and astrocytes, averaging 981,333 cells/mL and 1.03 million cells/mL, respectively (Figure 2B), in good agreement with the reactive microgliosis and astrogliosis expected during a neurodegenerative process with loss of neuronal processes. In view of the two-fold increase in microglial and astroglial cell numbers, normalized transcript levels of any markers of microglia and astroglia mass would therefore be expected around 200% even in the absence of reactive astrogliosis processes that imply active transcriptional upregulation. No difference in neuronal cell numbers was observed.

Upon RT-qPCR quality control analyses of purity in fractions from the 10-month-old brains, the factors studied included microglia markers (*Aif1*, *Tmem119*), an astrocyte marker (*Gfap*), a neuron marker (*Map2*), endothelial cell markers (*Cldn5*, *Cd31*), an oligodendrocyte marker (*Olig2*), and a pericyte marker (*Kcnj8*) (Figure S1).

### 3.3. Further Purity Analyses of Isolated Individual Brain Cell Types

**Figure 3:**
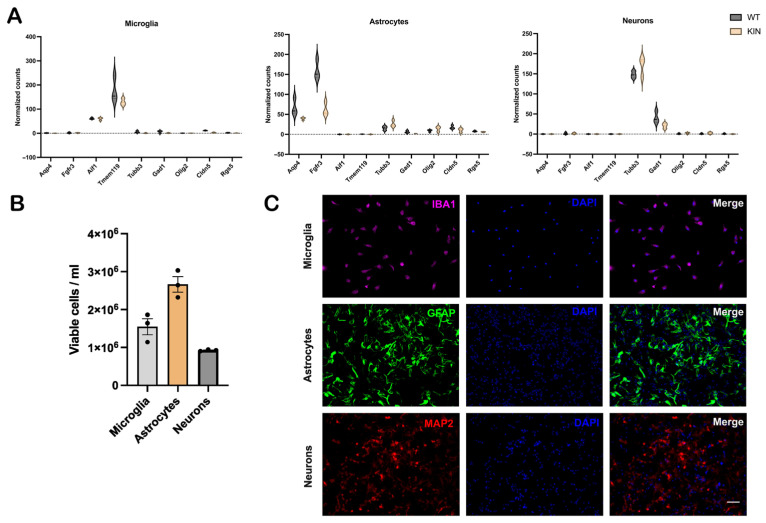
Purity validation of isolated microglia, astrocytes, and neurons. (**A**) Expression of specific markers in the gene sequencing data of 10-month-old mice. (**B**) Viable cell counts from 3-day-old wild-type mice (*n* = 3). Data are mean ± SEM. (**C**) Immunofluorescence staining of microglia with the marker IBA1, astrocytes with GFAP, and neurons with MAP2 after 5 days in culture. DAPI was used for staining the nucleus. Scale bar: 5 µM.

Given that microglia have the physiological function to prune synapses and protect them from injury [112,113,114], mechanically disconnected synapses will co-purify with microglia. Similarly, astrocytes function to surround and protect synapses as well as Ranvier nodes from pulsating blood vessels, modulating the morphology of synapses, axons, and dendrites [115,116,117], so astrocytes will also co-sediment with severed neural projections. Finally, it is known that the non-labeled flow-through cells will not only contain neurons, but also endothelial cells [118]. Therefore, the purity of the isolated cell populations was further assessed by analyzing the expression of a complementary set of specific marker mRNAs in the RNA-seq output from 10-month-old brains as violin plots (Supplementary Material S1 and Figure 3A). Here, *Aqp4* and *Fgfr3* were documented as astroglia (as well as radial glia and ependymal cells) markers, *Aif1* and *Tmem119* as microglia markers, *Tubb3* as neuronal differentiation marker, *Gad1* as interneuron marker, *Olig2* as oligodendroglia marker, *Cldn5* as endothelial marker, and *Rgs5* as pericyte marker.

Microglia and neuron populations were approximately 90% pure; however, in the astrocyte population, there was a minor presence of *Map2*, *Cldn5*, *Olig2*, and *Kcnj8*. Importantly, data from brainrnaseq.org (last accessed on 5 February 2026) indicates that these latter transcripts exhibit mild expressions in astrocytes, which could explain their appearance in the RT-qPCR results [119].

Immunocytochemistry was performed to further validate the isolated cells on a cytological level. 3-day-old pups were used for cultivation (Figure 3B), since cells from 10-month-old mice showed reduced survivability. All cells were plated in a 24-well plate with the following seeding density: microglia at 2 × 10^5^ cells/well, astrocytes and neurons at 3 × 10^5^ cells/well. All isolated cells survived in culture for ~7 days post-isolation and seeding. However, the morphology and growth were best at two days for microglia and five days for astrocytes and neurons. Anti- IBA1, GFAP, and MAP2 antibodies were used for microglia, astrocytes, and neurons, respectively, in immunofluorescence staining (Figure 3C). All isolated cell types showed a cell-type-specific morphology and cellular marker expression.

### 3.4. Global Volcano Plots of RNA-Seq Profiles, as Well as Consistency Between New RNA-Seq Transcript Analysis and Previous Oligonucleotide Microarray Data Document Loss of mRNA in Synapses and Neurites from Glial Fractions, with an Elevated Neuroinflammation Signature

The extraction of RNA yielded sufficient amounts in astroglial and microglial fractions, but lower amounts in the neuronal fraction (Table S1) made it impossible to use the standard RNA-seq protocol. Therefore, in the present study, sequencing libraries of all fractions were prepared using the highly sensitive MACE-seq (Massive Analysis of cDNA Ends-sequencing) protocol with the inclusion of additional rRNA depletion and polyadenylation steps, thus allowing the capture of all RNA molecules simultaneously in comparable quality, including small RNA. The company GenXPro uses the term”panRNA-Seq” for this combination of MACE-Seq with polyadenylation. The standard RNA-seq procedure with traditional protocols uses hexamer primers to generate cDNA and analyze mRNAs in 10–30 fragments, thus generating shorter cDNA products, requiring at least 100 ng RNA as well as length-based normalization in typically 20 million reads. In contrast, the MACE-seq technique (which was developed in 2008 and optimized repeatedly, with >100 citations at present) focuses on a single highly specific fragment at the 3′-end of each mRNA where alternative splicing and alternative polyadenylation are frequent, with equivalent analytic depth being achieved from 0.03 ng samples at much lower cost, even in degraded RNA [104,120] and in circulating tumor cells (https://edocs.tib.eu/files/e01fb24/1896619908.pdf, last accessed on 21 March 2026) even at single-cell resolution.

In this manner, the expression in the neuronal fraction was quantifiable for 12,000 genes, despite the low cell number and RNA amount there. While the data are therefore comparable among all fractions, only the more abundant neuronal transcripts may reveal consistency with glial transcripts.

**Figure 4:**
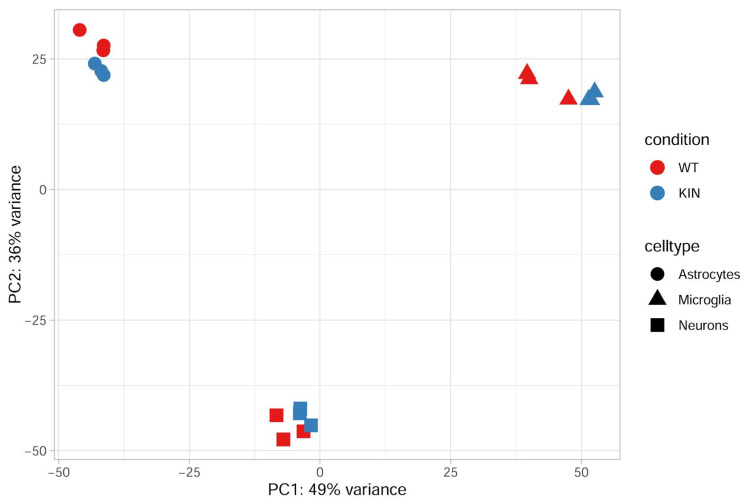
Principal component analysis (PCA) of RNA-seq data from astrocytes, microglia, and neurons derived from WT and KIN mice. PC1 (49% variance) and PC2 (36% variance) are shown. This plot shows potential clusters based on gene expression.

In general, the assessment confirmed the high quality of data, with a quantitative detection of 21,375 transcripts (isoforms of 16,387 genes) in the different fractions. Relevant quality control steps for each fraction involved FastQC 0.11.9 and MultiQC 1.32, including principal component analyses (Figure 4) as well as heatmaps showing Pearson Correlation Coefficients based on expression of the top 500 genes among all samples, boxplots of Cook’s distances from DESeq2 analysis to illustrate the distributions of values across samples, and dispersion plots from DESeq2 analysis to show gene-wise, fitted, and final dispersion estimates (Supplementary Materials S1 and S2).

Within each cell type, WT and KIN samples formed distinct but closely related subclusters (Figure 4), suggesting genotype-dependent transcriptional alterations that are smaller in magnitude compared to cell-type-specific differences. Importantly, biological replicates clustered tightly together, demonstrating high data consistency and experimental reproducibility.

**Figure 5:**
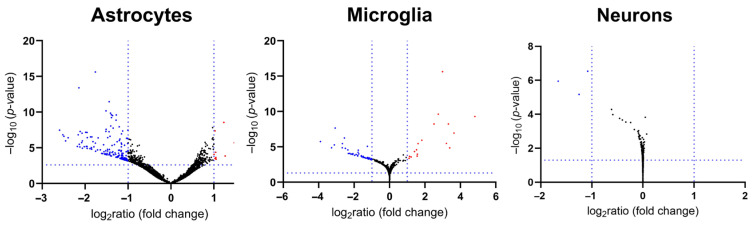
Volcano plots of different cell populations from 10-month-old *Atxn2*-CAG100-KIN mouse brains. Log2-fold changes and -log10 (*p*-values) of all sequenced genes are provided. Downregulations are shown in blue and upregulations in red.

As documented by global Volcano plots (Figure 5), decreased mRNA levels dominated in all fractions. Mainly, the microglial fraction showed strong upregulations. The differential expression analysis across all KIN brain fractions as compound DESeq2 aggregate, with subsequent GSEA documentation of enriched pathways, highlighted the “transmission across chemical synapses” in the Reactome database, “voltage-gated channel activity” among GO-MF terms, “regulated exocytosis” among GO-BP terms, and “synaptic membrane” among GO-CC terms, as prominently downregulated mRNA clusters (Supplementary Material S1).

To filter the crucial data that are reproducible (independent of the transcriptomic techniques used) and are correlates of the disease process (independent of the region of the nervous system under analysis), all novel RNA-seq transcript data from 10-month-old brain fractions with dysregulations of nominal significance were compared to all previously documented [101] dysregulations of nominal significance in 14-month-old spinal cord Affymetrix Clariom D oligonucleotide microarray data. It is important to note that the unstable repeat in *Atxn2*-CAG100-KIN mice is continuously expanding over successive generations since the generation of this mutant [100], and an ever earlier onset of locomotor deficits was observed. It is also relevant that the spinal cord is affected earlier in SCA2 than the cerebellum and much earlier than the rest of the brain. Thus, both datasets were selected to represent disease-relevant stages rather than strictly age- or tissue-matched conditions. It must be considered that the spinal cord consists of many neuronal projections with myelination but relatively few neurons, resulting in a transcriptional profile that differs significantly from that of the brain. Consequently, this comparison of both approaches may identify the dysregulation of crucial molecules that are invariably associated with the SCA2/ALS13 disease process in any neuro-glial tissue, but risks ignoring many relevant molecular adaptations (particularly those with tissue-specificity).

Initial assessment of markers for specific compartments among the new RNA-seq transcript isoform profiles may provide a notion about data consistency and also about the severity of the disease process at the age under analysis. This comparison also enables the normalization of RNA-seq data, to distinguish which abnormalities will be due to the loss of neural projections and their corresponding myelin sheaths, versus increased glia mass together with reactive astrogliosis and microgliosis, or alternatively represent truly active transcriptional regulations.

Firstly, neuronal loss was substantial with reduced levels for the dendrite markers *Map1b* (log2FC −1.70 in astroglial, and −1.60 in microglial fraction) and *Mapt* (log2FC −2.10 in microglial fraction), postsynaptic marker *Nrgn* (log2FC −2.03 and −3.64, respectively), extracellular postsynapse marker *Nptx1* (log2FC −3.31 and −2.53, respectively), presynaptic markers *Snap25* (log2FC −4.43 in astroglial fraction) and *Gap43* (log2FC −2.69 in astroglial fraction), axonal marker *Nefl* (log2FC −0.65 and −0.30, respectively), neuron cytoskeleton marker *Sptan1* (log2FC −2.04 in microglial fraction), neuron soma marker *Eno2* (log2FC −1.37 and −1.17), neuron nucleus marker *Rbfox3* (encoding NeuN, log2FC −0.50 and −0.12), glutamatergic neuron marker *Slc17a7* (log2FC −1.85 and −0.65), glutamatergic neuron markers *Gls* (log2FC −0.33 and −0.003) and *Grin1* (log2FC −0.55 and −0.37), GABAergic interneuron markers *Gad1* (log2FC −0.42 and −0.08) and *Gad2* (log2FC −0.33 in astroglial fraction), glutamatergic neuron marker *Neurod2* (log2FC −0.50 in astroglial fraction), and interneuron marker *Pvalb* (log2FC −0.21 in astroglial fraction). Overall, the strongest dysregulation among markers of neuronal loss was found for the postsynaptic marker *Nrgn* and the presynaptic marker *Snap25*, suggesting that synaptic components may be present only at around levels of 5% in some compartments of the mutant brain.

Secondly, astrogliosis was not conspicuous, with astrocyte markers *Sox9*, *Gs*, *Acsbg1*, *C3* and *Stat3* at normal levels, *Gfap* showing almost normal levels (log2FC 0.33 without significance in astroglial fraction), astrocyte markers *Vim* (log2FC 1.05 and 0.005, respectively) and *S100b* (log2FC 0.59 and 0.01) showing significant upregulations, but astrocyte markers *Aldh1l1* (log2FC −0.50 in astroglial fraction), *Ndrg2* (log2FC −1.1 in astroglial fraction), *Slc1a2* (log2FC −1.27 in astroglial fraction), and *Aqp4* (log2FC −0.47) showing significant downregulations. Overall, the strongest dysregulations among markers of reactive astrogliosis were found for *Vim* and *Slc1a2*, with approximately two-fold changes up and down, corresponding to the increased astroglial cell number in the mutant brain.

Thirdly, microgliosis markers *Aif1*, *Trem2*, *Hexb*, *Sall1*, *Olfml3*, *Hexb*, *Fcrls*, *Spi1*, *Cx3cr1*, the microglia homeostasis marker *P2ry12*, and the mature microglia marker *Tmem119* appeared unchanged, while the *Trem2*-signaling partner *Tyrobp* exhibited a statistical trend towards upregulation (log2FC 0.02 in microglial fraction). Lysosomal *Cd68* as a marker of microglial activation showed a significant upregulation (log2FC 0.73 in microglial fraction). Importantly, selected known markers of reactive microgliosis, like *Trem2*-dependent *Apoe*, *Cst3*, *Cst7*, *Lpl*, *Clec7a*, *Axl*, *Spp1*, *Lgals3*, *Lgals3bp*, *Itgax*, *Cd63* and *Gpnmb*, as disease-associated microgliosis markers, showed massive and strongly significant upregulations that are detailed below. Overall, the strongest dysregulation among markers of reactive microgliosis was found for *Gpnmb* (log2FC 4.38, corresponding to 2082%), well above the two-fold increase in cell number documented for microglia in the mutant brain.

A significantly reduced expression of the disease gene *Atxn2* with the CAG-repeat expansion was documented only in the microglial fraction (log2FC −0.03).

Overall, the constitutive markers of astroglial and microglial mass did not exhibit increases in proportion to the two-fold increase in their cell numbers, suggesting partial efficacy of compensatory experimental efforts (with equal RNA amounts being used for cDNA preparation, and sequencing data being normalized). There was a prominent loss of synaptic components and a massive upregulation of several disease-associated microgliosis markers.

Within the global comparison of microarray with RNA-seq profiles, the consistent downregulations (Table S2A) showed the strongest effects for (i) the 0.06-fold reduction in *Scn4b* encoding the sodium voltage-gated channel beta subunit 4 (also known as Navbeta4 or LQT10), in both tissues and in the astrocytic fraction. The SCN4B protein regulates the depolarization of excitable membranes [121]. Other consistent prominent decreases were documented for (ii) the synaptic factor *Snap25* (encoding the Synaptosome-Associated Protein 25, as t-SNARE complex member involved in the molecular regulation of neurotransmitter release) in both tissues and astrocytic fraction, (iii) the synaptic factor *Unc13c* (encoding Munc13-3, as exocytosis factor in the presynaptic active zone), as well as (iv) the neurite transport factor *Kif5a* (encoding the Neuronal Kinesin Heavy Chain, as a microtubule-dependent motor is required for slow axonal transport) again in both tissues and astrocytic fraction. In addition, a strong decrease of (v) *Hmgcr* (encoding 3-hydroxy-3-methylglutaryl-CoA reductase, as the rate-limiting enzyme for cholesterol synthesis, regulated via negative feedback by sterols) was apparent in both tissues and the astrocytic fraction. Overall, these downregulations provide molecular insights into the detrimental changes that affect the functions of the astrocyte–microglia–synapse–neurite unit.

Among the consistent upregulations (Table S2B), the strongest effects were observed in both tissues and the microglia fraction, regarding (i) *Gpnmb* to log2FC of 4.38, equal to 2082% (encoding the glycoprotein nonmetastatic melanoma protein B, also known as hematopoietic growth factor inducible neurokinin-1, as integrin binding factor associated with amyloidosis type PLCA3), (ii) *Cst7* (encoding cystatin-F, as protease-inhibitory factor associated with cerebral amyloid angiopathy), (iii) *Clec7a* (encoding the C-type lectin domain containing protein 7A, also known as CD369, as pattern-recognition receptor in the innate immune system that recognizes a variety of beta-1,3-linked and beta-1,6-linked glucans from fungi and plants, activating NF-kappa-B and MAP kinase p38 pathways and enhancing cytokine production), (iv) *Slc11a1* (encoding the solute carrier family 11, member 1, as proton-coupled divalent metal ion transporter that sequestrates Fe^2+^ and Mn^2+^, localized to late-endosomal/lysosomal membranes), (v) *Lgals3* (encoding lectin, galactoside-binding, soluble factor 3, as advanced glycation end-product receptor that coordinates the recognition of membrane damage with mobilization of core autophagy regulators), (vi) *Lgals3bp* (encoding galectin-3 binding protein, as factor that promotes integrin-mediated cell adhesion and stimulates host defense). Overall, these upregulations provide molecular insights into the mechanisms of the reactive microgliosis in a neurodegenerative process.

### 3.5. RNA-Seq Transcript Consistency Analysis Among Glial Fractions Shows a Prominent Increase in Transcripts Encoding Cytoplasmic Ribosomal Proteins of Large and Small Subunits

Thus, the new RNA-seq profiles showed consistency with previously validated microarray data. Given that RNA-seq findings may reveal RNA processing changes that cannot be detected by rigid oligonucleotide approaches, the subsequent effort considered only RNA-seq consistency. For this purpose, all RNA-seq data were filtered to identify effects with reproducibility between the overall brain compound RNA-seq, the astrocytic fraction, and the microglial fraction.

Among the consistent downregulations (Table S3A), known glial factors (*Ndrg4*, encoding N-myc downstream-regulated gene 4 protein, required for mitogenic signaling and cell cycle progression), but also synaptic factors (*Slc17a7* encoding the vesicular glutamate transporter 1, *Cplx1* encoding complexin 1, *Syt1* encoding syntaxin 1, *Scnb* encoding synuclein beta), axonal factors (*Nefh* below the log2FC of −2.4 in the microglial fraction, corresponding to 19%, and with *Nefl*, both encoding neurofilament subunits), and dendritic factors (*Nptx1* encoding neuronal pentraxin 1 as AMPA receptor clustering factor) were prominent, as unspecific correlates of the neurodegenerative process. As the strongest impact by the polyQ-expansion of the translation modulator ATXN2, with immediate credibility, strong decreases in *Cyfip2* (encoding cytoplasmic FMR1 interacting protein 2) and *Eef1a2* (encoding eukaryotic translation elongation factor 1 alpha 2, as a neuron-specific, monomeric, adult paralog) were observed. In view of the effect of mutant cytoplasmic ATXN2 on *Cyfip2* expression here, it is noteworthy that knockout of its more nuclear paralog ATXN2L was reported to selectively deplete the levels of *Nufip2* (encoding nuclear FMR1 interacting protein 2) [67].

Among the consistent upregulations (Table S3B), prominent effects included the inductions of immunological factors *Usp18* (encoding ubiquitin specific peptidase 18, as interferon-induced ISG15-specific processing protease) and *Ifitm3* (encoding the interferon-induced transmembrane protein 3, as antiviral protein that disrupts intracellular cholesterol homeostasis), as well as *Eef1a1* (encoding the eukaryotic translation elongation factor 1 alpha 1, as a ubiquitous, dimeric, embryonic paralog), while practically all other consistent increases were mild and concerned selectively the cytoplasmic ribosomal factors, both for the large and the small subunit.

### 3.6. STRING Analysis of Interactions and Bioinformatics Enrichments in Glial Fractions Extends the Observations of Transcript Reduction in Synapses, Axons, and Dendrites, Versus Induction of Ribosomal and Inflammatory Factors

Analyzing the consistent dysregulations in brain compound, astroglial, and microglial fractions further, and evaluating comprehensively also medium and small effects, not only the strong effects, an additional bioinformatics STRING interaction and pathways enrichment analysis was performed.

**Figure 6:**
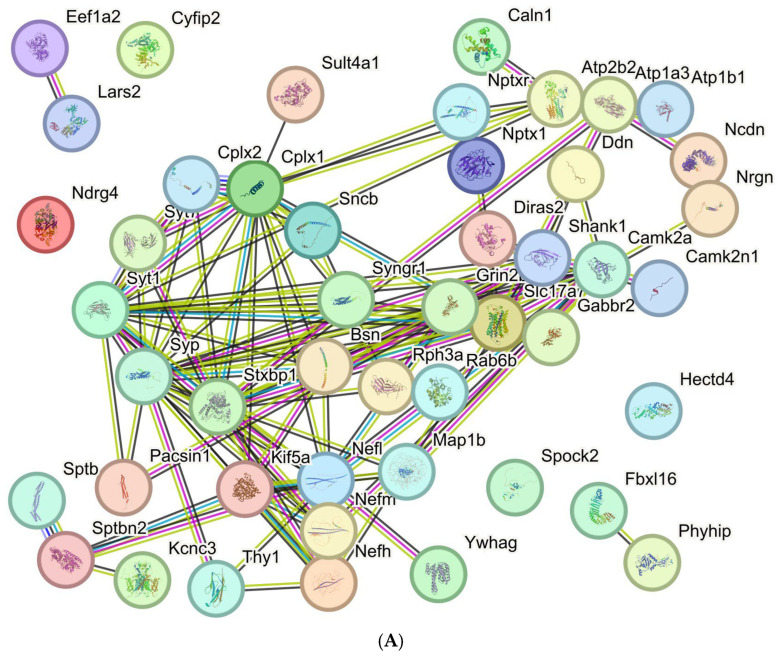

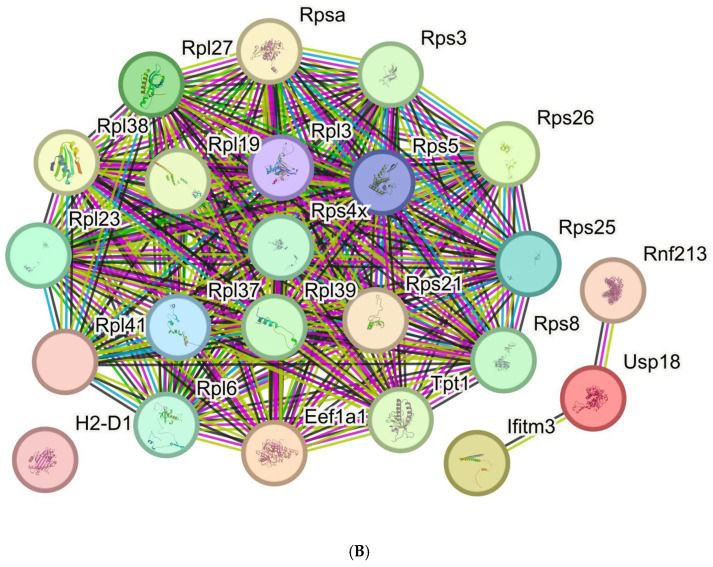
(**A**): STRING protein–protein interaction diagram of all downregulated transcripts that are consistent between brain compound, astroglial, and microglial fractions, upon RNA-seq of 10-month-old *Atxn2*-CAG100-KIN mice; (**B**): STRING protein–protein interaction diagram of all upregulated transcripts that are consistent between brain compound, astroglial, and microglial fractions, upon RNA-seq of 10-month-old *Atxn2*-CAG100-KIN mice.

Regarding consistent downregulations, statistically significant deficits were found (Figure 6A) for transcripts in presynapses (Gene Ontology term Cellular Compartment 0098793, false discovery rate 4.55 × 10^−19^), postsynapses (GOCC 0098794, FDR 6.40 × 10^−18^), dendrites (GOCC 0030425, FDR 3.50 × 10^−16^), axons (GOCC 0030424, FDR 1.09 × 10^−16^), with an overall protein–protein-interaction enrichment *p*-value < 1.0 × 10^−16^. It was noteworthy that *Spock2* (encoding secreted protein acidic and rich in cysteine, or SPARC, as a glial and neuronal factor that is usually induced during diseases or injury [122]) showed decreased levels. It was also interesting to note that reduced *Fbxl16* (encoding F-box and leucine-rich repeat protein 16, as a C-myc stabilizer [123]) was in direct correlation with the reduction in *Ndrg4* (encoding an N-myc downstream-regulated factor [124]), suggesting altered growth signaling in the myc oncogenic pathway.

Regarding consistent upregulations, statistically significant deficits were found (Figure 6B) for cytoplasmic translation (GO term Biological Process 002181, FDR 4.82 × 10^−29^) and for ribosome assembly (GO term Biological Process 0042255, FDR 6.27 × 10^−6^), with an overall protein-protein-interaction enrichment *p*-value < 1.0 × 10^−16^.

### 3.7. RNA-Seq Consistency Analysis Among Glial Fractions for Small RNAs Finds Upregulation of All tRNAs Versus Downregulation of All Antisense RNAs and All Processed Pseudogenes

Performing also a small RNA sequencing analysis (sRNA-seq, including, e.g., antisense RNA, lincRNA, miRNA, mt-tRNA, piRNA, processed pseudogene RNA, processed transcripts, small protein coding mRNA, pseudogene RNA, retained intron RNA, ribozyme RNA, scaRNA, sense intronic RNA, snoRNA, tRNA and unprocessed pseudogene RNA), again consistency criteria were applied to define effects in direct correlation among astroglial and microglial fractions. Details on quality control assessment are provided in Supplementary Material S2.

Exclusively, downregulations were observed for two sRNA classes: antisense RNA and processed pseudogene RNA (Table S4A). The strongest effect concerned Gm36043-201, a predicted long non-coding RNA documented in the Ensembl genome browser to contain two exons on mouse chromosome 19.

Exclusively upregulations were observed for one sRNA class: cytoplasmic tRNAs (Table S4B). These effects were all mild. The relatively biggest effect concerned tRNAs for threonine.

### 3.8. RNA-Seq Analysis at the Gene Level of Microglia-Specific Effects Reveals ~Eight-Fold Accumulation of Uridine-Rich Small Nuclear RNA Rnu1b2

Given that the dysregulations were much stronger for microglial than for astroglial factors, we explored them without consistency filtering, based on gene-based analysis rather than on the previous transcript-based analysis that takes different isoforms into account. This slightly different strategy identified various dysregulations that are specific to microglia and not recognized in brain homogenate as a complex tissue (Table S5A). This approach, without consistency evaluation, has great potential to identify cell-specific dysregulations that get diluted in the complete tissue, but it is also the most artifact-prone strategy, so future validation will be critical. Given that reactive microgliosis and astrogliosis are expected during any neurodegenerative processes, moderately increased glial mRNA levels (below two-fold) must be viewed with caution. During this study, great care was taken to use equal amounts of RNA for cDNA preparation and to normalize the sequencing data, but of course, the relative decrease in neuronal transcripts may cause a converse relative increase in glial transcripts in any normalized homogenate. Clearly, increased mass or number of glial cells may also act as confounders (see Figure 2), and pathological RNA accumulation due to a processing block upon *Atxn2* mutation may be present, so increased expression levels are not necessarily due to transcriptional upregulations.

In addition to the previously mentioned strong upregulations of *Gpnmb*, *Cst7*, *Clec7a*, *Slc11a1*, *Lgals3*, and *Lgals3bp*, further strong inductions were noted for *Itgax*, *Spp1*, *Apoe*, *Axl*, and below two-fold inductions for *Csf*, *Cd63*, *Fam20c*, *Lpl*, and *Mpeg1*, as a probably unspecific neuroinflammatory signature. Interestingly, a potentially *Atxn2* mutation-specific massive mutation effect was noted for the pre-mRNA splicing factor *Rnu1b2*, which increased 14-fold (log2FC of 3.8, corresponding to 1390%), well above the levels that can result from the two-fold increase in microglia cell numbers. This uridine-rich small RNA is a potential target of the LSM domain in ATXN2, so this novel exploratory observation is interesting and merits future follow-up experiments, although it is microglia-specific and currently unconfirmed.

In addition to the previously mentioned strong downregulations of the glial factor *Ndrg4* and of several synaptic/axonal/dendritic factors, further strong reduction was noted for the long non-coding small nucleolar RNA *Rian*, which was previously found downregulated in Parkinson’s disease and shown to modulate its neuroinflammatory process [125]. Furthermore, the observation of reduced *Syne1* levels has particular credibility given that the SYNE1 protein was previously reported to co-accumulate with ATXN2 aggregates in the *Atxn2*-CAG100-KIN mouse brain [67].

### 3.9. RNA-Seq Analysis at the Gene Level of Astroglia-Specific Effects Mainly Documents the Neurite Retraction

To gain similar insights also in the astroglia-specific effects, again gene-based analysis was conducted and showed milder dysregulations (Table S5B).

The expression increases were close to the two-fold threshold that could be explained by relative enrichment of glial transcripts upon relative loss of neuronal transcripts in any normalized homogenate, and by increased astrocyte mass during a process of reactive astrogliosis.

In addition to the previously mentioned decreases in *Cplx1*, *Camk2b*, *Snap25*, *Ndrg4*, *Kif5a*, *Eef1a2*, only the new decreases in *Agt* (encoding Angiotensinogen, also known as Serine Proteinase Inhibitor A8, as potent regulator of blood pressure, body fluid and electrolyte homeostasis) and *Etnppl* (encoding ethanolamine-phosphate phospho-lyase, as cellular response factor to glucocorticoid stimulus, implicated in the ceramide phosphoethanolamine catabolic process and the phospholipid transfer to membranes) represent astrocyte-enriched transcripts, while the other decreases affect inhibitory neuron factors (*Dnm1*, *Sptbn2*, *Adcy1*, *Kcnq2*, *Slc12a5*, *Celsr3*, *Cplx2*, *Unc13a*, *Trank1*, *Camk2a*, and *Atp1a3*) or excitatory neuron factors (*Mical2*, *Ppfia4*, and *Slc17a7*), according to http://www.braincellatlas.org (last accessed on 23 January 2026) cerebellar data. Overall, the reactive astrogliosis appears to be of little relevance in comparison to the strong reactive microgliosis and the massive neurite retraction according to these RNA-seq data.

### 3.10. RNA-Seq Analysis at the Gene Level of Neuron-Specific Effects Documents Novel DNA Effects

To also gain similar insights into the neuron-specific effects, pathway enrichments were considered instead of a tabular compilation of the strongest dysregulation, in view of the low fold-changes that are usual for neurons.

Expression increases in most histone isoforms led to significant enrichment for several DNA damage response pathways (Supplementary Material S1, Figures S17–S20, Tables S16–S19). Although these exploratory data are unconfirmed, they represent the first evidence that DNA handling and repair mechanisms are relevant for the disease course in SCA2, as was previously reported for Huntington’s disease [126,127,128].

Conversely, the expression decrease in many mitochondrial factors was reflected by significant enrichment for several pathways of respiratory activity and oxidative phosphorylation (Supplementary Material S1, Figures S17–S20, Tables S16–S19). Certainly, the general loss of respiration-dependent neuronal tissue in the aged SCA2 mouse model may have contributed to these decreases.

### 3.11. Systematic Interrogation of RNA-Seq Findings at the Gene Level for Uridine-Rich snRNAs and the SMN Pathway Shows Increases Selectively in the Astroglial Fraction That Could Be Explained by Astrogliosis

To assess if the strong *Rnu1b2* accumulation in microglia was a unique finding or recapitulated for other uridine-rich snRNAs with a weaker effect size in any fraction, a systematic compilation of all *Rnu* gene family data is shown in Table S6. Indeed, many *Rnu* family members were detected, sometimes with two isoforms and additional pseudogenes. But the strong *Rnu1b2* accumulation in microglia was not recapitulated for other *Rnu* variants destined to the spliceosome. Curiously, several of these *Rnu* genes showed significantly but mildly increased expression, and two isoforms showed converse decreases, always in the astroglia-enriched fraction. Here, it has to be taken into account that astrogliosis is present in the mutant brains, and that effect sizes with log2FC values around 0.4 can simply be due to the relative increase in glial transcripts due to the relative decrease in neural transcripts in any normalized homogenate. They need not be explained by transcriptional upregulation or by accumulation due to impaired processing.

To also understand if the pathways of spliceosome biogenesis and subsequent splice activities are affected, we interrogated the RNA-seq data for dysregulations of components of (i) the SMN complex that acts as assembly site for the spliceosome, (ii) the Sm proteins within the spliceosome, and (iii) the PRMT5 complex that is responsible for the methylation of Sm proteins to form 6S Sm-subcomplexes that get loaded onto snRNA by the SMN complex [129]. Significant changes were only found in the astroglial fraction for *Gemin7* (log2FC 1.80), *Snrpd1* (log2FC 1.73), and *Snrpd3* (log2FC 1.36), while *Smn1*, *Gemin2*, *Gemin4*, *Gemin8*, *Strap*, *Snrpd2*, *Snrpf*, *Snrpg*, *Prmt5*, *Clns1a* and *Wdr77* showed non-significant results, and all other components were not detected. Thus, these upregulations do not appear connected with the microglial Rnu1b2 excess and are within the range of changes that occur during reactive astrogliosis.

## 4. Discussion

Recent research showed that most of the disease genes underlying the neurodegenerative processes of Alzheimer’s disease and Parkinson’s disease are strongly expressed in glial cells (astrocytes, microglia, and oligodendrocytes, not only neurons) [115,130]. In the case of SCA2/ALS13, it is well established that the responsible disease protein Ataxin-2 is best detected in the vulnerable neurons due to their large cytoplasm, but it is ubiquitously expressed according to comprehensive databases (www.proteinatlas.org, last accessed on 23 January 2026) and gets transcriptionally induced during stress periods of glial cells [131,132]. However, the role of glial cells upon polyQ-expansion or knockout of Ataxin-2 and its orthologs/paralogs has not been studied previously. All transcriptomic, proteomic, phospho-proteomic, lipidomic, and metabolomic studies done so far were in whole tissue homogenates or organismal extracts in humans, mice, flies, yeast, and plants [66,67,68,69,72,82,83,86,88,96,99,101,133,134,135,136,137,138,139]. As a matter of course, relevant mutation effects in specific cell types may get diluted and obscured when complex tissue extracts are used for subsequent analyses.

The present study successfully enriched astroglial and microglial fractions. In contrast, the neuronal fraction showed 20-fold fewer cell numbers and a low RNA yield. This prompted the highly sensitive MACE protocol for RNA-seq to be employed for all fractions, achieving the quantification of >10,000 among the most abundant transcripts even in the neuronal fraction. It remains unclear whether the significance of dysregulations is detected with similar efficacy in the neuronal fraction as in the two glial fractions, where sequencing depth and the number of unique reads were much higher. Fraction purity was assessed by RT-qPCR and immunocytochemistry, showing an approximately two-fold higher glial cell number from mutants, despite the lower brain weight of mutants. Both glial fractions from 10-month-old *Atxn2*-CAG100-knockin mice, as an authentic model of SCA2/ALS13, were compared. Overall, the data indicate that the loss of synaptic/axonal/dendritic transcripts is prominent at this stage (with *Nefh* mRNA for the neurofilament heavy chain transcript as an axonal factor down in glial fractions to below 19%), and microglial dysregulations are strong (with the *Gpnmb* transcript encoding Osteoactivin up to above 2000%), while astroglial dysregulations are minor. The unspecific signs of the neurodegenerative process include the induction of a microglial neuroinflammatory signature including not only *Gpnmb*, but also *Cst7*, *Clec7a*, *Slc11a1*, *Lgals3*, *Lgals3bp*, *Itgax* (CD11c), *Spp1* (Osteopontin), *Apoe*, *Axl*, *Usp18*, *Ifitm3*, *Rnf213*, in a response pattern that has been observed also in neurodegenerative processes such as Alzheimer’s, Fronto-temporal dementia, and Parkinson’s disease, as well as nervous system trauma and infarction [140,141,142,143,144,145,146,147,148,149,150,151,152,153,154,155]. These findings lead us to propose that disease severity and progression in our SCA2 mouse model should be quantified by measuring the ratio between *Gpnmb* increase and *Nefh* decrease. These nonspecific findings in glial cells showed high consistency and are in excellent agreement with the robust literature on neuroinflammatory signatures in other neurodegenerative disorders, so they are very credible.

There were also specific dysregulations that relate to the altered function and interactome of the disease protein ATXN2, firstly at the translation apparatus. Strong decreases in the *Cyfip2* and *Eef1a2* transcripts were documented, with a parallel increase in *Eef1a1*, suggesting that polyQ expansion of ATXN2 selectively impairs the translation at the initiation and re-initiation (i.e., elongation) steps. In good agreement, ATXN2 has a known direct protein interaction with PABPC1 (polyA-binding protein, cytoplasmic, type 1), which associates with the 3′ tail of mRNAs to mediate their circularization, in a step before ribosome recruitment and translation initiation, and their association is affected by the polyQ expansion [65]. The two translation elongation factor paralogs are 97% identical. *Eef1a2* serves as a monomeric, adult form with selective neuronal expression and high phosphorylation, particularly on tyrosine residues, with established functions in neuronal signaling (e.g., mTOR-dependent axonal repair). In comparison, *Eef1a1* functions as a dimeric, embryonic ubiquitous form with a seven-fold higher GDP dissociation rate, with known roles for cytoskeletal modifications [156]. The *Cyfip* family is thought to modulate translation initiation, with the isoform *Cyfip1* acting at the 5′ end while *Cyfip2* targets the 3′ end of mRNAs, affecting mainly the expression of actin cytoskeleton factors that are crucial for synaptic plasticity [157]. This deleterious effect of polyQ-expansions in ATXN2 on mRNA circularization and re-initiation of translation for elongation efficiency may be the underlying cause for the transcriptional upregulation of cytoplasmic ribosomal factors in the large and small subunits, and of the cytoplasmic tRNAs. These mild but systematic and consistent RNA-seq observations are likely to represent a cellular compensation effort to maintain sufficient protein biosynthesis. However, a partial substitution of deficient neuronal *Eef1a2,* which mediates the necessary stimulus-dependent protein synthesis for synapses, by enhanced levels of embryonal high-yield *Eef1a1* and elevated levels of translation machinery (ribosomes and tRNA) would be inadequately governed by the excitatory versus inhibitory signals within synapses. Overall, these data elucidate how ATXN2 polyQ expansion impacts the translation regulation steps, in similar ways as was reported for ATXN2-null mice [69,70,72]. Although these data are novel and are complementary to previously published knowledge on ATXN2, we have to caution that independent validation in patient tissue or other SCA2 models, with additional techniques, is necessary to establish their value.

Secondly, there were additional specific dysregulations that relate to ATXN2 functions in RNA processing upstream from translation events. The finding that significantly dysregulated small RNAs invariably showed decreases for all antisense RNAs and for all processed pseudogenes indicates that the impact of ATXN2 polyQ expansion is not restricted to translation. This notion is supported by the deficient levels of the long non-coding small nucleolar RNA *Rian*. Apparently, the processing of non-translated sRNAs is affected by cytoplasmic ATXN2, even if an RNA like *Rian* is trafficked to the nucleolus rather than the cytoplasm. Of course, indirect effects might be responsible, but the exploratory observation of a 13.9-fold accumulation of *Rnu1b2* levels provides an interesting potential explanation via a direct mechanism. ATXN2 structure is dominated by its N-terminal LSm-fold with five beta-barrels. LSm domains are derived from bacterial *Escherichia coli* Hfq/YlxS and archaeal Sm1/Sm2 proteins, which function as RNA chaperones, with preferential binding to regulatory small uridine-rich RNAs, and crucial roles for spliceosome evolution and ribosomal translation [62,158,159,160,161]. Five primary uridine-rich small nuclear RNAs are present in the spliceosome (U1, U2, U4, U5, and U6), having an essential role for pre-mRNA splicing [162,163], and another uridine-rich snRNA (U7) modulates histone mRNA 3′ end maturation [164]. The murine *Rnu1b2* snRNA (corresponding to human *RNU1-2*) represents one isoform of the murine U1 snRNA [165]. U1 RNA is essential for recognizing the 5′ splice site of mRNA precursors to facilitate intron removal [163,166]. *Rnu1b2* and its paralog *Rnu1b1* are chromosomally located five kilobases apart, coded on opposite DNA strands, with juxtaposed 5′ ends, and share identical flanking sequences 5′ and 3′ to the gene [167,168,169,170,171]. *Rnu1b* paralogs show embryonic and fetal expression, while *Rnu1a* paralogs show constitutive expression in differentiated cell types [172,173,174]. It remains unclear what the functional differences between *Rnu1b2* and *Rnu1b1* might be. Crucially, the uridine-rich snRNAs undergo several processing steps. U1, as a Sm-class snRNA, is transcribed by RNA polymerase-II and receives a 7-methylguanosine (m^7^G) cap in the nucleus. It is then exported to the cytoplasmic U-bodies, where 3′-end trimming occurs together with cap hypermethylation to 2,2,7-trimethylguanosine (m_3_G). U-bodies form upon cellular stress in the vicinity of P-bodies, where 5′-cap processing and 3′-end trimming also occur, but are taken further until the complete degradation of RNA [175,176,177]. For P-bodies, a perturbation upon experimental manipulation of ATXN2 was already documented [63]. In U-bodies, the SMN complex recognizes the 3′-end trimming of uridine-rich snRNAs and assembles their Sm sites with seven Sm proteins (B/B’, D3, D2, D1, E, F, and G) [178]. The resulting ribonucleoprotein complex is re-imported into the nucleus to become the spliceosome [175,179,180,181]. Clearly, the U1 processing step at U-bodies via the SMN complex provides an opportunity where cytoplasmic ATXN2 with its LSm domain has access to uridine-rich snRNAs before they become part of the spliceosome. In this context, it is interesting to note that mutations in the SMN1 protein as part of the SMN complex result in axonal motor neuron degeneration with fetal, pediatric or juvenile onset [182,183,184,185,186,187], and mutations in ATXN2 cause pathologically indistinguishable axonal motor neuron degeneration with adult onset [7,101,188,189,190], in good agreement with these thoughts about a functional interaction between ATXN2 and SMN1 at U-bodies. Altogether, the observed about 14-fold accumulation of the small nuclear *Rnu1b2* isoform seems a plausible specific effect of ATXN2 polyQ expansion, which was documented only in microglial fractions by gene-based analysis, and may have been diluted in previous global transcriptome surveys of complex nervous tissues that were focused on mRNAs. While it is tempting to interpret the increased levels of *Rnu1b2* as a consequence of impaired processing in U-bodies due to altered conformation of the LSm domain in ATXN2 upon polyQ expansion, of course it should also be considered that this 14-fold increase may represent an active transcriptional upregulation in response to stress, possibly secondary to microglial activation, in analogy to the 2082% induction of *Gpnmb*. Thus, at present is remains completely unclear whether the *Rnu1b2* is part of the pathology, or part of compensatory neuroprotective efforts in the brain tissue. It is important to know that U1 snRNPs act as potent triggers of innate immunity in monocytes, having established relevance for autoimmune pathology and (for the neuroinflammatory process, or for stress-triggered splice adaptations?) in Alzheimer’s disease [191,192,193,194,195]. Furthermore, it is noteworthy that U1 snRNAs were previously proposed as useful biomarkers [196] and that their manipulation has therapeutic value [197,198,199,200,201,202,203,204]. Despite the novelty and the plausible mechanistic consequences of the *Rnu1b2* increase, this preliminary, single-fraction observation strongly requires independent validation before it can be considered a relevant molecular event within SCA2/ALS13 pathogenesis. The scenario of ATXN2-LSm interaction with *Rnu1b2* at U-bodies is worthwhile considering, but at this stage purely speculative.

In this context, another isolated observation without consistency in the current RNA-seq dataset becomes noteworthy. Only in the neuronal fraction, where the quantification may be reliable only for very abundant transcripts, the levels of *Eftud2* showed a significant accumulation (log2FC 4.37). As a component of the minor spliceosome, the putative GTPase protein EFTUD2 (also known as U5 snRNP Specific Protein with 116 KD, or SNU114 Homolog) is integrated in the U5 snRNP and the U4/U6-U5 tri-snRNP complexes, serving to enable the splicing of U12-type introns in pre-mRNAs [205,206,207]. *Eftud2* dysregulation is particularly deleterious for cerebellar Purkinje neurons [208,209] and has a prominent role in antiviral splice adaptations [210,211]. However, this RNA-seq observation is clearly exploratory and cannot provide mechanistic insights at present without further confirmation.

Thirdly, there was a specific dysregulation that relates to ATXN2 function, regarding the highly dynamic actin cytoskeleton at the nuclear envelope, in addition to the synaptic terminals. The finding of reduced *Syne1* levels was previously observed together with a reduction in SYNE1 protein levels and a co-accumulation of SYNE1 protein with the ATXN2 aggregates in aged *Atxn2*-CAG100-KIN mouse brain [67], as mentioned above. This previous report analyzed knockout mice of ATXN2L as a nucleus–cytoplasm shuttling paralog of ATXN2 [67], observing a prominent impact on the actin modulator SYNE2 (also known as Nesprin-2) at the nuclear envelope [212], and on the nuclear protein NUFIP2 as a target of an O-GlcNAcylation-mediated condensation in the context of actin-binding and focal adhesion factors [213]. Parallel investigations of conditional ATXN2L-depletion at adult age documented a clear alteration of the nuclear splicing pathway [68]. This knowledge is now complemented by novel RNA-seq evidence that the polyQ expansion in the completely cytoplasmic paralog ATXN2 causes reductions in *Syne1* and *Cyfip2* levels. As functional similarities, both giant SYNE/Nesprin proteins have paired N-terminal calponin-homology (CH) domains for actin binding and C-terminal KASH domains for membrane anchoring. These domains are encoded in multiple isoforms that are subject to alternate splicing, getting positioned in overlapping localizations at nuclear and other membranes (where they ensure actin-dependent proximity towards synaptic signals or other stimuli). These domains also regulate cell polarity and filopodia [214,215,216,217,218]. As a functional difference, SYNE2 was found to be critical for cortical and hippocampal neurons, while SYNE1 is more relevant for cerebellar neurons, with its mutations resulting in spinocerebellar ataxia type SCAR8 and motor neuron degeneration [219,220,221]. Regarding the other gene family with dysregulated expression upon ATXN2/ATXN2L mutations, both the NUFIP2 and CYFIP2 proteins are interactors of the Fragile X Mental Retardation protein (FMRP), as members of a family of regulators of neuronal translation, of activity-dependent bulk endocytosis, and of synaptic function, acting as adaptors of ribonucleoprotein complexes and actin [222,223,224,225,226,227]. NUFIP isoforms are thought to connect FMRP with nuclear export, RNA processing, the control of protein synthesis at synaptic sites, and the autophagolysosomal degradation pathway [228,229,230,231,232,233,234], while CYFIP isoforms connect FMRP with translation suppression (CYFIP2 acting at the 3′ untranslated region of mRNAs), actin polymerization, and cytoskeleton remodeling [157,235,236,237,238,239,240,241,242,243,244,245,246,247]. The impact of Ataxin-2 family members on the NUFIP/CYFIP family might explain previous findings in the nematode *Caenorhabditis elegans* that deletion of its single-copy Ataxin-2 ortholog regulates centrosomes at the nuclear envelope and cytoskeletal dynamics [248]. These observations are also in good agreement with observations in the fly *Drosophila melanogaster* that its single-copy Ataxin-2 ortholog interacts with FMRP [98,249] and impacts the actin cytoskeleton [56]. Again, these data are novel and are complementary to previously published knowledge on ATXN2, but independent validation in patient tissue or other SCA2 models, with additional techniques, will be needed to establish their value.

Fourth, there were very consistent downregulations of *Sptbn2* in both glial and neuronal fractions (Tables S3A and S5A). This is remarkable in view of the roles of ATXN2 and SPTBN2 as disease proteins responsible for spinocerebellar ataxia. Mutations in SPTBN2 cause autosomal dominant spinocerebellar ataxia type 5 (SCA5) and autosomal recessive SCAR14/SPARCA1 [250,251,252,253]. SPTBN2 is a structural constituent of the postsynapse, essential for anchoring glutamate receptors and transporters (such as EAAT4 and mGluR1) at the plasma membrane [254,255]. It helps shape the dendritic spine neck, regulating synaptic transmission to prevent excessive excitation [256,257]. SPTBN2 plays a vital role in trafficking proteins, maintaining the structural integrity of the Golgi apparatus, and managing vesicle transport within neurons [258,259,260]. In particular, it supports cerebellar Purkinje cells [261,262]. The impact of ATXN2 polyQ expansion on the expression of beta-spectrin isoforms in general, and on their roles to connect the cortical actin cytoskeleton with synaptic membranes via ankyrin-binding and pleckstrin-homology domains [263,264], was substantiated by the significant decreased expression (Table S3A) of *Sptb* (log2FC −2.36 in astroglial and −3.78 in microglial fraction), *Sptbn4* (log2FC −1.4 in astroglial, −1.23 in microglial fraction) and *Sptbn1* (log2FC −0.72 in microglial fraction). Similar to the statements above, these data are novel and are complementary to previously published knowledge on ATXN2, but independent validation in patient tissue or other SCA2 models, with additional techniques, will be needed to establish their value.

There are clear limitations of this study. Unfortunately, not enough aged *Atxn2*-CAG100-KIN mice were available to obtain sufficient material in the neuronal fraction for standard RNA-seq(given that the progressive expansion in the polyQ domain of ATXN2 has an ever more profound impact on fertility due to its expression changes in peripheral tissues [69,100]), so the highly sensitive MACE technique was employed instead across all fractions and the data therefore reflect only the 3′-ends of mRNA. Thus, only a small part of all isoforms is represented in the expression profiles. While all the RNA profiles reported here are preliminary (given that we were unable to perform validation experiments in additional samples with complementary techniques), we endeavored to ensure that neuronal data above the detection threshold are similarly powered and of comparable quality as the glial data. The advanced neurodegeneration in the aged mice under study causes a loss of neural tissue in parallel to reactive microgliosis and astrogliosis, so it is impossible to distinguish clearly whether lower neural transcript levels and higher glial transcript levels are due to altered cell mass/cell numbers, or indeed reflect active transcriptional regulation. Although we made efforts to compare crucial data with markers of microglia and astroglia mass/numbers and to visualize these background effects in a heatmap where massive transcriptional dysregulations are easily appreciated, validation experiments, e.g., regarding the promoter methylation pattern of key genes, should be conducted in the future. It will also be useful to assess crucial data such as the increased expression of *Gpnmb* and the decreased expression, e.g., of *Nefh* and of *Cyfip2* at the protein level, employing quantitative immunoblots, immunohistochemistry, and coimmunoprecipitation experiments. Our team discontinued breeding of the *Atxn2*-CAG100-KIN mouse line upon closure of the lab due to retirement of the principal investigator, but the sperm is cryoconserved at the company genOway (Lyon, France) and at the European Mouse Mutant Archive (EMMA ID EM:13598, see https://www.infrafrontier.eu/emma/strain-details/?q=13598, last accessed on 3 February 2026), so that independent teams can assess the present data. Finally, it is clearly possible that RNA-seq findings about SCA2/ALS13-unique dysregulations that are not reproduced by oligonucleotide microarray data, or RNA-seq data in a single fraction (such as the accumulation of *Rnu1b2*, or the deficiency of *Rian*) might be technical or analytical artifacts.

Potential next experiments might of course include (i) the analysis of frozen or fresh patient samples such as nervous tissues (very few are available, with their preservation state always being a cause of concern) or the analysis of patient peripheral blood macrophages, (ii) the analysis of independent SCA2/ALS13 models in mice, flies, nematodes, zebrafish, yeast, or plants, (iii) the analysis of independent SCA2/ALS13 models in vitro, such as microglial cell lines, cultures of primary microglia, extracts from microglial fractions. For cell culture experiments, it should be considered that the microglia-specific dysregulations described here may have arisen only after the exposure of these cells to stress and debris, while the effects may not be detectable in complex brain tissue and cell cultures during normal growth phases. Clearly, the maturation of uridine-rich snRNAs and their incorporation into spliceosome precursors by the SMN complex is concentrated in U-bodies only after cellular stress, while it occurs diffusely in the cytoplasm of unstressed cells [265,266,267]. Beyond microglia- and astroglia-enriched fractions, it would be desirable to breed and age the *Atxn2*-CAG100-KIN mouse line again, complementing the present data with the analysis of neuronal and oligodendroglial fractions, to understand more cell-type-specific disease mechanisms in view of the strong and early neurite retraction and demyelination in SCA2 [101,268,269,270,271,272,273]. If the evidence about non-coding small RNA dysregulations is substantiated, a systematic study about the processing of all uridine-rich snRNAs, snoRNAs, antisense RNAs, and pseudogenes should follow. Crucially, it is worthwhile considering the possibility that a lack of completely processed uridine-rich snRNAs would impair spliceosomal activity, and that synthetic substitutes with slow turnover due to pseudouridylation might provide a causal therapeutic opportunity [274,275,276,277,278,279]. As stated before, all these confirmatory studies with different biological material and varying technical approaches will have to be conducted by independent research teams. 

## 5. Conclusions

In conclusion, the transcriptome profiling from microglia- and astroglia-enriched fractions from 10-month-old *Atxn2*-CAG100-KIN brain documented a severe retraction of neural terminals from their glia-association, together with strong reactive microgliosis at this stage, while the astrogliosis was mild, as unspecific signatures of the neurodegenerative process. As potential SCA2/ALS13-specific dysregulations that relate to the known phenotype, functions, and interactomes of ATXN2, four new effects complement previous findings. (i) Altered levels were documented for two translation elongation factors in converse direction; (ii) reduced levels were found for a cytoplasmic member of the CYFIP family (in analogy, its nuclear homolog is affected by ATXN2L mutation); (iii) decreased levels were also observed for another member of the Nesprin/SYNE family that mediates membrane movement (in analogy, a second member is affected by ATXN2L mutation); and (iv) decreased transcript levels were observed for several members of the beta-spectrin family. The functional roles of all these molecules converge in the stimulus-dependent mRNA splicing and translation, particularly for actin-associated cytoskeletal factors, to enable plasticity of synaptic as well as nuclear membranes. The impact of ATXN2 polyQ expansion was not restricted to coding mRNAs, but among small non-coding RNAs, mild but consistent effects were documented for all antisense RNAs and all processed pseudogenes, which invariably showed downregulations, similar to the small nucleolar non-coding RNA *Rian*. As a single, exploratory, and currently unvalidated observation about possible mechanisms that mediate this impact upstream from cytoplasmic ribosomal translation, the 13.9-fold accumulation of the uridine-rich small nuclear RNA *Rnu1b2* stands out. As a plausible scenario, the mutation directly adjacent to the LSM domain of cytoplasmic ATXN2 could affect its binding and processing of uridine-rich RNAs as its known targets, when they are exported from the nucleus to cytoplasmic U-bodies for hypermethylation of their 5′ cap, and trimming of their 3′ ends. Subsequently, the processed U-rich snRNAs will interact with the SMN complex, so that they can assemble with the seven Sm proteins to form the spliceosome, before this ribonucleoprotein complex gets re-imported into the nucleus and assumes its activity there. Unfortunately, this microglia-specific effect could not be validated further by this team, and future assessment by independent groups is urgently needed.

## Data Availability

Complete analyses in EXCEL files supporting the conclusions of this article, as well as MultiQC reports, are available from the authors upon reasonable request. All raw RNA-seq data were deposited publicly at GEO with the accession numbers GSE319876 and GSE319875.

## Supplementary Materials

The following supporting information can be downloaded at: https://www.mdpi.com/article/10.3390/cells15080699/s1. Supplementary Material S1: Quality assessment of RNA-seq data for mRNA. Supplementary Material S2: Quality assessment of RNA-seq data for small RNA. Figure S1: RT-qPCR analyses of dissociation purity in CD11b-labeled microglial fraction, ACSA2-labeled astroglial fraction, and unlabeled neuronal/endothelial fraction. Table S1: RNA concentrations per sample, before RNA-seq. Table S2A,B: Consistency analysis between the new 10-month-old brain compound and cell-type-specific RNA-seq transcript profiles and previously reported 14-month-old spinal cord oligonucleotide microarray data. Table S3A,B: Consistency analysis between new RNA-seq profiles in brain compound, astroglial, and microglial fractions. Table S4A,B: Consistency analysis between new sRNA-seq profiles in astroglial and microglial fractions. Table S5A: Strongest dysregulations in the new RNA-seq gene profile of microglial fraction. Table S5B: Strongest dysregulations in the new RNA-seq profile of astroglial fraction. Table S6: Compilation of expression data for Rnu gene family.

## Abbreviations

The following abbreviations are used in this manuscript:

ACSA2	Astrocyte Cell Surface Antigen-2
Adcy1	Adenylate Cyclase 1
Agt	Angiotensinogen, Serine Proteinase Inhibitor A8
Aif1	Allograft Inflammatory Factor 1 = IBA1
Ald1l1	Aldehyde Dehydrogenase 1 Family Member L1
ALS13	Amyotrophic Lateral Sclerosis type 13
AMPA	Alpha-amino-3-hydroxy-5-methyl-4-isoxazolepropionic acid
Apoe	Apolipoprotein E
Atp1a3	ATPase Na+/K+ Transporting Subunit Alpha 3
Atx2	Ataxin-2 in flies
Atxn2	Ataxin-2
Atxn2l	Ataxin-2-like
Axl	AXL Receptor Tyrosine Kinase
Camk2a	Calcium/Calmodulin-Dependent Protein Kinase II Alpha
Camk2b	Calcium/Calmodulin-Dependent Protein Kinase II Beat
Cd32	Cluster of Differentiation 32
Cd63	Cluster of Differentiation 63
Cd68	Cluster of Differentiation 68
cDNA	Complementary DNA
Celsr3	Cadherin EGF LAG Seven-Pass G-Type Receptor 3
CH	Calponin-Homology
CID3/4	Chemical-Inducible Dimerization
Cldn5	Claudin 5
Clec7a	C-type lectin Domain-Containing Protein 7A
Cplx1	Complexin 1/2
Cst7	Cystatin-F
Cyfip2	Cytoplasmic FMR1 Interacting Protein 2
Ddx6	DEAD-Box Helicase 6
Dnm1	Dynamin 1
Eaat4	Excitatory Amino Acid Transporter 4, Solute Carrier Family 1 Member 6
EDTA	Ethylenediaminetetraacetic Acid
Eef1a1/2	Eukaryotic translation elongation factor 1 alpha 1/2
Eftud2	Elongation Factor Tu GTP Binding Domain Containing 2
Eno2	Enolase 2
Etnppl	Ethanolamine-phosphate phospho-lyase
Fam20c	Family With Sequence Similarity 20, Member C
Fbxl16	F-box and leucine-rich repeat protein 16
FC	Fold Change
Fcrls	Fc Receptor-Like S
FDR	False Discovery Rate
FMRP	Fragile X Mental Retardation Protein
Gad1/2	Glutamate Decarboxylase 1
GDP	Guanosine diphosphate
Gfap	Glial Fibrillary Acidic Protein
Gls	Glutaminase
GO	Gene Ontology
GO-BP	Gene Ontology—Biological Process
GO-CC	Gene Ontology—Cellular Component
GO-MF	Gene Ontology—Molecular Function
Gpnmb	Glycoprotein Nmb
Grin1	Glutamate Receptor, Ionotropic, N-Methyl D-Aspartate 1
GSEA	Gene Set Enrichment Analysis
Hexb	Hexosaminidase Subunit Beta
Hmgcr	3-Hydroxy-3-Methylglutaryl-CoA Reductase
Hmgcs1	3-Hydroxy-3-Methylglutaryl-CoA Synthase 1
IBA1	Ionized calcium-binding adapter molecule 1, = AIF1
Ifitm3	Interferon-induced transmembrane protein 3
Itgax	Integrin Subunit Alpha X
Kcnj8	Potassium Inwardly Rectifying Channel Subfamily J Member 8
Kcnq2	Potassium Voltage-Gated Channel Subfamily Q Member 2
Kif5a	Neuronal Kinesin Heavy Chain
KIN	Knockin
Lgals3	Lectin, galactoside-binding, soluble factor 3
Lgals3bp	Galectin-3 binding protein
lincRNA	Long intergenic non-coding RNA
Lpl	Lipoprotein Lipase
LSmAD	LSm-Associated Domain
MACE	Massive Analysis of cDNA Ends
MACS	Magnetic-Activated Cell Sorting
Map1b	Microtubule-Associated Protein 1B
Map2	Microtubule-Associated Protein 2
mGLUR1	Glutamate Metabotropic Receptor 1
Mical2	Microtubule-Associated Monooxygenase, Calponin and LIM Domain-Containing 2
miRNA	MicroRNA
Mpeg1	Macrophage Expressed 1
Mt-tRNA	Mitochondrial transfer RNA
Ndrg4	N-myc downstream-regulated gene 4
Nefh	Neurofilament Heavy Chain
Nefl	Neurofilament Light Chain
Neurod2	Neuronal Differentiation 2
NGS	Next-Generation Sequencing
Nptx1	Neuronal Pentraxin 1
Nufip2	Nuclear FMR1-interacting protein 2
Olfml3	Olfactomedin-Like 3
Olig2	Oligodendrocyte Transcription Factor 2
P2ry12	Purinergic Receptor P2Y12
PABP	Poly(A)-binding protein
Pabpc1	polyA-binding protein, cytoplasmic, type 1
PAR-CLIP	Photoactivatable Ribonucleoside-Enhanced Crosslinking and Immunoprecipitation
PCA	Principal component analysis
piRNA	Piwi-interacting RNA
Plca3	= Gpnmb, Glycoprotein Nonmetastatic Melanoma Protein B
Ppfia4	PPFI Scaffold Protein A4
Pvalb	Parvalbumin
Rab15	Ras-Related Protein Rab-15
Rbfox3	RNA Binding Fox-1 Homolog 3
Rian	RNA Imprinted and Accumulated in Nucleus
RNA	Ribonucleic Acid
Rnf213	Ring Finger Protein 213
Rnu1b2	RNA, U1 Small Nuclear 2
Rph3a	Rabphilin 3A
rRNA	Ribonucleic acid
RT-qPCR	Reverse Transcriptase Quantitative Real-Time Polymerase Chain Reaction
S100b	S100 Calcium Binding Protein B
Sall1	Spalt-Like Transcription Factor 1
SCA2	Spinocerebellar Ataxia type 2
SCA5	Spinocerebellar Ataxia Type 5
SCAR14	Spectrin Beta, Non-Erythrocytic 2
scaRNA	Small Cajal body-specific RNA
Scn4b	Sodium voltage-gated channel beta subunit 4
SEM	Standard error of the mean
Slc11a1	Solute carrier family 11, member 1
Slc12a5	Solute Carrier Family 12 Member 5
Slc17a7	Vesicular glutamate transporter 1
SMN1	Survival Of Motor Neuron 1, Telomeric
Snap25	Synaptosome-associated Protein 25
snoRNA	Small Nucleolar RNA
snRNA	Small nuclear RNA
snRNP	Small nuclear ribonucleoprotein
SPARCA1	Spectrin-Associated Autosomal Recessive Cerebellar Ataxia
Spi1	Spi-1 Proto-Oncogene
Spp1	Secreted Phosphoprotein 1
Spock2	secreted protein acidic and rich in cysteine
Sptbn1	Spectrin Beta, Non-Erythrocytic 1
Sptbn2	Spectrin Beta, Non-Erythrocytic 2
sRNA	Small RNA
STRING	Search Tool for Recurring Instances of Neighboring Genes
Syne1	Spectrin Repeat Containing Nuclear Envelope Protein 1
Syt1	Syntaxin 1
Tmem119	Transmembrane Protein 119
Trank1	Tetratricopeptide Repeat And Ankyrin Repeat Containing 1
Trem2	Triggering Receptor Expressed On Myeloid Cells 2
tRNA	Transfer RNA
Tyrobp	TYRO Protein Tyrosine Kinase-Binding Protein
Unc13a	Unc-13 Homolog A
Usp18	Ubiquitin-Specific Peptidase 18
Vim	Vimentin
WT	Wild-type
ZFE	Central Animal Facility
