# Supplementary material for: Astro-Versus Microglia-Enriched Transcriptomes from Aged Atxn2-CAG100-Knockin Mice Suggest Underlying Pathology of RNA Processing at Ribosomes, and Possibly at U-Bodies
Source: Cells. 2026 Apr 15;15(8):699. doi: 10.3390/cells15080699 (PMC13115098; doi:10.3390/cells15080699)
Supplement: Supplementary file 1 [file cells-15-00699-s001.zip › AuburgerKandi_Supplements.pdf]

Table S1

RNA concentrations of the samples used:

No	Sample	Type	c (ng/µl)
1	WT-1	Microglia	14.6
2	WT-2	Microglia	11.25
3	WT-3	Microglia	10.04
4	KIN-1	Microglia	31.05
5	KIN-2	Microglia	43.4
6	KIN-3	Microglia	44.3
7	WT-1	Astrocytes	13.15
8	WT-2	Astrocytes	25.65
9	WT-3	Astrocytes	15.25
10	KIN-1	Astrocytes	34.85
11	KIN-2	Astrocytes	21.1
12	KIN-3	Astrocytes	24.55
13	WT-1	Neurons	0.2
14	WT-2	Neurons	0.84
15	WT-3	Neurons	0.58
16	KIN-1	Neurons	1.24
17	KIN-2	Neurons	0.85
18	KIN-3	Neurons	1.18

Table S2A

id	gene symbol	log2FC brain RNAseq	p value brain RNAseq	log2FC brain astrocytes	p value brain astrocytes	log2FC brain microglia	p value brain microglia	log2FC brain neurons	p value brain neurons	log2FC spinal cord ClariomD microarray
**ENSMUST00000109815**	Camk2b	-4.07	3.89E-04	-4.39	2.51E-03					-1.6
**ENSMUST00000166358**	Ndrg4	-4.01	3.50E-05	-4.10	7.68E-04	-5.33	2.07E-04			-1.73
**ENSMUST00000125486**	Snap25	-4.00	2.56E-06	-4.43	1.54E-03					-2.31
**ENSMUST00000060125**	Scn4b	-3.91	2.06E-04	-4.37	2.61E-03					-4.72
**ENSMUST00000093369**	Nefh	-3.67	2.03E-05	-2.94	1.16E-03	-2.40	2.23E-02			-2.05
**ENSMUST00000129040**	Cplx1	-3.39	2.43E-02							-2.56
**ENSMUST00000021459**	Rab15	-3.08	2.53E-03	-2.94	3.92E-02					-1.43
**ENSMUST00000108951**	6430548M08Rik (KIAA0513)	-3.06	2.43E-04	-2.68	1.95E-02					-1.79
**ENSMUST00000028335**	Grin1	-2.95	4.61E-02	-3.68	1.03E-01					-1.67
**ENSMUST00000217895**	Kif5a	-2.90	2.12E-04	-2.16	9.10E-04					-3.59
**ENSMUST00000075245**	Unc13c	-2.87	7.60E-03							-4.21
**ENSMUST00000026357**	Jph3	-2.80	1.28E-04	-3.61	4.69E-03					-1.52
**ENSMUST00000036825**	Sncb	-2.73	1.30E-04	-2.12	2.89E-03	-6.01	1.91E-03			-1.49
**ENSMUST00000170287**	Hmgcr	-2.71	1.38E-02	-2.38	3.81E-02					-2.11
**ENSMUST00000202326**	Rph3a	-2.70	3.41E-03	-3.03	2.44E-02					-3.14
**ENSMUST00000026670**	Nptx1	-2.63	6.24E-04	-3.31	3.27E-05	-2.54	1.52E-02			-2.39
**ENSMUST00000231669**	Pacsin1	-2.52	3.67E-03	-2.68	7.45E-03					-1.49
**ENSMUST00000066791**	Tmem179	-2.44	4.41E-03							-1.6
**ENSMUST00000132013**	Msmo1	-2.35	2.38E-02	-2.30	8.97E-03					-5.25
**ENSMUST00000082365**	Sult4a1	-2.35	3.52E-03	-2.71	3.23E-03	-3.94	8.93E-03			-2.09
**ENSMUST00000160433**	Kcnc1	-2.28	6.80E-03	-4.39	2.51E-03					-1.64
**ENSMUST00000113222**	Stxbp1	-2.28	3.29E-03	-3.14	2.74E-03					-2.49
**ENSMUST00000065668**	Nrgn	-2.27	2.26E-03	-2.03	1.02E-03	-3.64	3.94E-06			-1.39
**ENSMUST00000008297**	Clstn3	-2.21	1.92E-03	-2.38	1.25E-02					-1.86
**ENSMUST00000202089**	Hsph1	-2.20	1.70E-02	-1.14	2.63E-01	-4.77	2.15E-03			-1.75
**ENSMUST00000045262**	Ak5	-2.12	3.55E-02	-2.32	6.52E-02					-1.72
**ENSMUST00000030643**	Extl1	-2.12	3.43E-02	-2.33	2.76E-03					-1.68
**ENSMUST00000111089**	Nefm	-2.09	4.74E-03	-3.71	4.05E-04	-2.09	3.22E-02			-1.52
**ENSMUST00000066708**	Dmp1	-2.00	4.15E-02	-2.35	4.79E-02					-1.92
**ENSMUST00000130481**	Plcxd2	-1.97	1.57E-02	-3.15	1.70E-01					-2.67
**ENSMUST00000061620**	Unc80	-1.95	1.24E-03	-1.98	6.94E-03					-2.71
**ENSMUST00000183482**	Htr1b	-1.94	2.00E-02							-1.78
**ENSMUST00000045738**	Slc32a1	-1.89	2.70E-02	-3.59	1.98E-02	-4.52	1.75E-01	-1.12	2.64E-01	-1.6
**ENSMUST00000076968**	Syt7	-1.79	7.57E-03	-2.04	1.48E-02	-3.24	1.54E-02			-1.38
**ENSMUST00000113728**	Klc2	-1.78	4.48E-02	-1.13	3.48E-01					-1.99
**ENSMUST00000120291**	Glns-ps1	-1.76	3.92E-02	-2.22	9.23E-03					-2.34
**ENSMUST00000107906**	Kcnc3	-1.75	2.50E-03	-1.98	2.15E-02	-2.27	3.23E-02			-3.19
**ENSMUST00000222185**	Rapgef5	-1.73	3.01E-02			-2.09	5.27E-02			-2.13
**ENSMUST00000170959**	Kif5b	-1.67	2.42E-02							-2.51
**ENSMUST00000114256**	Lingo1	-1.58	2.22E-02	-1.47	7.48E-02					-1.48
**ENSMUST00000225430**	Cables1	-1.56	2.30E-02							-1.92
**ENSMUST00000111288**	Caln1	-1.54	3.80E-04	-2.68	2.95E-04	-1.74	1.59E-02			-2.51
**ENSMUST00000121820**	Spock2	-1.53	4.01E-03	-1.56	1.09E-04	-2.41	8.63E-04	-0.62	5.36E-01	-1.91
**ENSMUST00000103075**	Nsf	-1.52	1.63E-02	-1.25	4.43E-02					-1.82

Table S2B

id	gene symbol	log2FC brain RNAseq	p value brain RNAseq	log2FC brain astrocytes	p value brain astrocytes	log2FC brain microglia	p value brain microglia	log2FC brain neurons	p value brain neurons	log2FC spinal cord ClariomD microarray
**ENSMUST00000031840**	Gpnmb	3.54	2.27E-04			4.38	4.00E-12			261.25
**ENSMUST00000089200**	Cst7	2.99	1.51E-02			4.03	1.62E-08			14.85
**ENSMUST00000195589**	Clec7a	2.74	3.65E-02			2.91	2.50E-11			13.78
**ENSMUST00000147233**	Slc11a1	2.33	3.71E-02			2.46	2.96E-04			1.9
**ENSMUST00000150290**	Lgals3	2.07	1.07E-02			2.50	1.18E-02			1.83
**ENSMUST00000043722**	Lgals3bp	2.05	1.62E-03	0.81	1.72E-01	2.68	2.01E-03			2.22
**ENSMUST00000045756**	S100a10	2.01	1.45E-02					3.65	1.32E-02	1.35
**ENSMUST00000057795**	Rsrc2	1.85	1.36E-02							1.54
**ENSMUST00000079294**	Ifi27	1.82	1.97E-02	2.58	4.13E-03					1.59
**ENSMUST00000201456**	Wdr1	1.81	4.27E-02							1.32
**ENSMUST00000154169**	Rell1	1.75	5.05E-03	2.42	1.84E-02	1.43	1.97E-01			1.23
**ENSMUST00000138037**	Irf9	1.70	1.99E-02							1.24
**ENSMUST00000048010**	Dse	1.64	3.79E-02			1.64	8.79E-02			1.79
**ENSMUST00000186693**	Anapc13	1.60	1.23E-02	2.13	4.98E-02					1.28
**ENSMUST00000092620**	Gm10275	1.59	2.42E-03			1.37	1.01E-01			1.8
**ENSMUST00000006764**	Aprt	1.57	2.97E-02			1.54	1.80E-01			1.45
**ENSMUST00000002677**	Axl	1.54	4.58E-02	0.04	9.45E-01	2.67	7.82E-08			1.77
**ENSMUST00000116034**	Rpl31-ps10	1.53	2.40E-02							1.55
**ENSMUST00000142111**	Fuca1	1.50	1.20E-02	1.58	1.00E-01	1.97	1.34E-02			1.32
**ENSMUST00000200645**	Anapc5	1.49	1.03E-02	1.16	1.44E-01					1.3
**ENSMUST00000199689**	Mrpl52	1.47	1.27E-02							1.23
**ENSMUST00000027108**	Eef1b2	1.46	2.86E-02			1.82	7.23E-02			1.45
**ENSMUST00000032198**	Usp18	1.43	2.06E-02	2.99	2.76E-02	2.37	8.67E-03			1.5
**ENSMUST00000026565**	Ifitm3	1.39	4.96E-02	1.90	1.92E-04	1.04	1.88E-02			1.69
**ENSMUST00000000284**	Trim25	1.39	7.26E-03	1.37	1.42E-01	1.40	9.57E-02			1.87
**ENSMUST00000139165**	Rps12-ps9	1.39	2.67E-02			0.82	2.17E-01	3.16	3.96E-02	1.88
**ENSMUST00000028062**	Vim	1.37	2.53E-02	2.66	2.07E-04	-0.47	6.24E-01			2.88
**ENSMUST00000169905**	Mtdh	1.35	3.55E-02			1.18	1.47E-01			1.22
**ENSMUST00000119571**	Gm14586	1.33	4.31E-03	1.01	2.21E-01	1.00	2.31E-01	1.95	1.04E-01	1.74
**ENSMUST00000212412**	Myo9b	1.33	3.43E-02			1.84	3.31E-02			1.42
**ENSMUST00000093902**	Rnf213	1.31	4.83E-04	1.19	4.21E-02	1.37	1.01E-02	2.21	1.20E-01	1.91
**ENSMUST00000218127**	Myl6	1.30	1.16E-02	1.73	5.89E-04	0.59	4.84E-01	0.79	4.26E-01	1.32
**ENSMUST00000031625**	Arpc1a	1.30	2.54E-02	1.80	6.09E-03					1.32
**ENSMUST00000129380**	Eef1a1	1.29	8.60E-03	2.61	4.70E-03	1.40	1.95E-02			1.48
**ENSMUST00000034264**	Pgls	1.27	5.45E-04	1.35	4.16E-02	0.94	1.25E-01	1.52	9.22E-02	1.28
**ENSMUST00000233937**	Rpl10a	1.25	3.23E-03	0.94	2.84E-01	1.09	1.03E-01	1.19	1.48E-01	1.54
**ENSMUST00000202370**	Gm9794	1.25	8.99E-03	1.50	4.54E-02	2.17	7.83E-04	0.21	7.50E-01	1.53
**ENSMUST00000030051**	Txn1	1.25	2.35E-02	1.67	4.39E-02	0.85	4.53E-01			1.63
**ENSMUST00000072566**	Nme2	1.24	1.75E-02	1.98	6.54E-02	1.23	1.31E-01	0.28	7.91E-01	1.51
**ENSMUST00000057740**	Rpsa-ps10	1.22	1.78E-02			0.56	4.87E-01	2.52	6.96E-02	1.49
**ENSMUST00000118499**	Rps6-ps4	1.22	7.24E-03	0.42	6.31E-01	1.58	4.16E-02	1.16	2.26E-01	1.39
**ENSMUST00000085720**	Rpl36-ps3	1.21	1.62E-02	2.19	7.31E-03	1.42	1.02E-01	0.12	8.97E-01	1.59

Table S3A

id	gene symbol	log2FC brain RNAseq	p value brain RNAseq	log2FC brain astrocytes	p value brain astrocytes	log2FC brain microglia	p value brain microglia	log2FC brain neurons	p value brain neurons
ENSMUST00000166358	Ndrg4	-4.01	3.50E-05	-4.10	7.68E-04	-5.33	2.07E-04		
ENSMUST00000093369	Nefh	-3.67	2.03E-05	-2.94	1.16E-03	-2.40	2.23E-02		
ENSMUST00000209634	Slc17a7	-3.58	1.19E-04	-2.64	2.67E-02	-4.82	1.81E-03		
ENSMUST00000142017	Cyfip2	-3.29	2.69E-04	-2.71	1.87E-02	-4.44	6.04E-03		
ENSMUST00000046892	Cplx1	-3.20	1.76E-07	-3.37	2.72E-06	-5.08	2.21E-04	-2.52	6.32E-03
ENSMUST00000064054	Syt1	-2.81	5.02E-04	-2.17	1.12E-02	-4.34	2.95E-03		
ENSMUST00000036825	Sncb	-2.73	1.30E-04	-2.12	2.89E-03	-6.01	1.91E-03		
ENSMUST00000022639	Nefl	-2.72	6.49E-05	-2.26	8.01E-03	-3.71	3.21E-03		
ENSMUST00000026670	Nptx1	-2.63	6.24E-04	-3.31	3.27E-05	-2.54	1.52E-02		
ENSMUST00000055990	Eef1a2	-2.55	1.92E-04	-2.57	6.98E-06	-3.20	3.89E-05		
ENSMUST00000008991	Sptbn2	-2.46	2.92E-06	-2.94	1.31E-06	-3.82	1.15E-06	-0.46	6.20E-01
ENSMUST00000099172	Kif5a	-2.44	3.07E-04	-2.07	2.32E-05	-2.44	4.22E-03	-2.71	3.61E-02
ENSMUST00000057442	Diras2	-2.38	1.01E-03	-2.59	5.11E-04	-2.82	4.42E-03		
ENSMUST00000045896	Pacsin1	-2.37	3.84E-03	-2.21	2.89E-02	-2.49	3.99E-02		
ENSMUST00000082365	Sult4a1	-2.35	3.52E-03	-2.71	3.23E-03	-3.94	8.93E-03		
ENSMUST00000106116	Ncdn	-2.27	2.57E-04	-2.23	2.15E-03	-3.59	2.03E-04		
ENSMUST00000065668	Nrgn	-2.27	2.26E-03	-2.03	1.02E-03	-3.64	3.94E-06		
ENSMUST00000035208	Bsn	-2.26	8.40E-07	-2.89	3.50E-04	-2.62	8.70E-06	-0.98	1.92E-01
ENSMUST00000041318	Ndrg4	-2.20	1.59E-02	-2.97	1.05E-03	-3.83	1.51E-02		
ENSMUST00000202406	Rph3a	-2.17	6.17E-04	-2.41	1.30E-02	-3.48	6.68E-03	-1.80	3.37E-02
ENSMUST00000111089	Nefm	-2.09	4.74E-03	-3.71	4.05E-04	-2.09	3.22E-02		
ENSMUST00000075444	Ddn	-2.02	4.59E-03	-2.79	2.74E-02	-1.33	3.80E-02		
ENSMUST00000152831	Atp2b2	-2.00	8.61E-04	-2.66	7.13E-04	-3.25	1.33E-02		
ENSMUST00000102858	Atp1a3	-1.90	4.54E-03	-2.75	1.71E-04	-1.72	4.90E-02		
ENSMUST00000196684	Atp1a3	-1.90	1.41E-04	-2.16	2.91E-03	-1.75	2.01E-02		
ENSMUST00000003561	Phyhip	-1.90	1.22E-02	-1.62	1.03E-02	-4.28	1.20E-02		
ENSMUST00000107749	Gabbr2	-1.82	4.74E-03	-1.90	5.12E-05	-1.80	1.60E-02		
ENSMUST00000076968	Syt7	-1.79	7.57E-03	-2.04	1.48E-02	-3.24	1.54E-02		
ENSMUST00000107906	Kcnc3	-1.75	2.50E-03	-1.98	2.15E-02	-2.27	3.23E-02		
ENSMUST00000053880	Grin2b	-1.68	6.60E-04	-2.15	2.90E-04	-2.07	2.79E-03	-0.29	8.03E-01
ENSMUST00000077458	Stxbp1	-1.66	1.98E-03	-1.49	3.45E-02	-2.10	3.35E-02		
ENSMUST00000009727	Syngr1	-1.57	9.20E-04	-1.92	1.60E-03	-1.28	1.95E-02		
ENSMUST00000055808	Ywhag	-1.55	1.54E-04	-2.25	1.09E-05	-1.35	9.43E-03	-0.65	4.40E-01
ENSMUST00000111288	Caln1	-1.54	3.80E-04	-2.68	2.95E-04	-1.74	1.59E-02		
ENSMUST00000121820	Spock2	-1.53	4.01E-03	-1.56	1.09E-04	-2.41	8.63E-04	-0.62	5.36E-01
ENSMUST00000045692	Fbxl16	-1.52	3.41E-03	-2.72	1.95E-04	-2.47	1.89E-03	0.08	9.31E-01
ENSMUST00000050000	Stxbp1	-1.51	7.03E-04	-1.79	1.89E-02	-2.21	7.99E-03		
ENSMUST00000069520	Syp	-1.48	8.93E-03	-1.29	4.15E-02	-1.95	2.75E-02		
ENSMUST00000102888	Camk2a	-1.43	5.22E-03	-2.41	5.01E-04	-1.53	3.18E-02		
ENSMUST00000166101	Sptb	-1.43	3.96E-02	-2.36	6.65E-03	-3.78	1.36E-02		
ENSMUST00000114840	Thy1	-1.33	2.54E-02	-1.97	6.78E-03	-2.76	5.32E-03	-0.70	3.64E-01
ENSMUST00000064762	Map1b	-1.28	2.44E-03	-1.95	1.64E-06	-2.16	1.12E-04	-0.30	5.02E-01
ENSMUST00000035155	Rab6b	-1.23	4.54E-03	-1.73	1.16E-03	-1.02	3.01E-02	-0.38	7.10E-01
ENSMUST00000023057	Nptxr	-1.16	1.83E-02	-2.49	2.65E-04	-1.81	1.03E-02	1.19	2.52E-01
ENSMUST00000042614	Hectd4	-1.16	5.70E-03	-1.25	3.92E-03	-1.52	3.44E-03	-0.59	4.19E-01
ENSMUST00000026985	Cplx2	-1.16	2.37E-03	-1.90	4.36E-05	-0.97	3.56E-02	-0.23	7.51E-01
ENSMUST00000027863	Atp1b1	-1.13	1.46E-02	-1.09	2.43E-02	-2.46	2.13E-03	-0.69	3.35E-01
ENSMUST00000050918	Camk2n1	-1.02	3.99E-02	-0.97	5.94E-03	-1.31	1.05E-03		
ENSMUST00000107938	Shank1	-1.01	6.57E-03	-1.70	4.40E-04	-1.28	2.48E-02	-0.07	9.19E-01
ENSMUST00000038863	Lars2	-0.61	7.96E-03	-0.78	2.01E-03	-0.78	4.65E-03	-0.40	3.22E-02
ENSMUST00000217116	Lars2	-0.57	1.82E-02	-0.63	1.01E-02	-0.81	1.96E-03	-0.46	3.59E-02

Table S3B

id	gene symbol	log2FC brain RNAseq	p value brain RNAseq	log2FC brain astrocytes	p value brain astrocytes	log2FC brain microglia	p value brain microglia	log2FC brain neurons	p value brain neurons
**ENSMUST00000032198**	Usp18	1.43	2.06E-02	2.99	2.76E-02	2.37	8.67E-03		
**ENSMUST00000129380**	Eef1a1	1.29	8.60E-03	2.61	4.70E-03	1.40	1.95E-02		
**ENSMUST00000026565**	Ifitm3	1.39	4.96E-02	1.90	1.92E-04	1.04	1.88E-02		
**ENSMUST00000219591**	Rps26	1.02	2.17E-02	1.61	3.16E-02	0.99	3.02E-02	0.63	3.51E-01
**ENSMUST00000202370**	Gm9794	1.25	8.99E-03	1.50	4.54E-02	2.17	7.83E-04	0.21	7.50E-01
**ENSMUST00000152162**	Rpl27	1.01	9.96E-04	1.45	8.67E-03	1.26	1.11E-02	-0.01	9.84E-01
**ENSMUST00000151118**	Rpl23	0.97	1.76E-02	1.40	1.46E-02	1.15	6.38E-03	0.42	4.38E-01
**ENSMUST00000080300**	Rps25	0.81	9.44E-03	1.38	5.46E-03	0.97	1.98E-02	0.05	9.15E-01
**ENSMUST00000227635**	Rpl37	1.02	6.40E-03	1.36	1.08E-02	1.35	5.44E-03	0.33	5.09E-01
**ENSMUST00000108539**	Rps5	0.68	4.54E-02	1.31	1.17E-02	0.85	4.00E-02	-0.01	9.89E-01
**ENSMUST00000188025**	Rpl3	0.88	6.64E-03	1.30	1.36E-02	1.00	1.52E-02	0.24	6.62E-01
**ENSMUST00000172503**	H2-D1	1.08	3.70E-02	1.22	3.90E-02	1.16	1.98E-03	0.74	2.79E-01
**ENSMUST00000177163**	Rpl41	1.00	1.01E-03	1.20	2.09E-02	1.10	1.79E-02	0.44	5.51E-01
**ENSMUST00000093902**	Rnf213	1.31	4.83E-04	1.19	4.21E-02	1.37	1.01E-02	2.21	1.20E-01
**ENSMUST00000176010**	Rpl41	1.00	3.99E-04	1.19	1.37E-02	1.33	5.37E-03	0.21	7.04E-01
**ENSMUST00000059080**	Rps21	0.84	9.13E-03	1.18	2.78E-03	0.95	4.71E-03	0.24	6.02E-01
**ENSMUST00000176906**	Rpl41	0.94	1.10E-02	1.15	4.00E-02	1.40	1.46E-02	0.20	7.45E-01
**ENSMUST00000217317**	Rpsa	0.91	5.46E-03	1.15	9.74E-03	1.07	1.82E-03	0.43	3.20E-01
**ENSMUST00000106599**	Rpl38	1.08	1.75E-03	1.12	4.15E-02	1.09	1.11E-02	0.79	1.74E-01
**ENSMUST00000092425**	Rpl19	0.69	1.96E-02	1.08	4.86E-03	0.82	2.28E-02	0.15	7.00E-01
**ENSMUST00000134040**	Tpt1	1.03	3.40E-03	1.04	2.51E-02	1.24	6.71E-04	0.51	3.63E-01
**ENSMUST00000032998**	Rps3	0.86	5.75E-03	1.03	8.44E-03	1.06	1.07E-02	0.41	2.65E-01
**ENSMUST00000115231**	Rpl39	0.85	2.26E-02	1.02	3.29E-02	1.08	3.85E-03	0.26	6.17E-01
**ENSMUST00000137423**	Rps8	0.62	4.69E-02	1.00	1.98E-02	0.93	1.79E-02	-0.11	8.01E-01
**ENSMUST00000110894**	Tpt1	0.90	1.61E-02	0.99	4.50E-02	1.33	2.49E-04	0.19	6.85E-01
**ENSMUST00000033683**	Rps4x	0.75	4.09E-02	0.91	1.78E-02	0.98	7.89E-03	0.26	4.79E-01
**ENSMUST00000138621**	Rpl6	0.73	2.54E-02	0.89	4.95E-02	1.00	1.14E-02	0.18	6.80E-01

Table S4A

id	gene symbol	type	log2FC brain astrocytes	p value brain astrocytes	log2FC brain microglia	p value brain microglia	log2FC brain neurons	p value brain neurons
ENSMUST00000236682	Gm36043-201	antisense	-3.77	1.93E-02	-4.48	6.45E-03		
ENSMUST00000238303	Rian-229	antisense	-1.30	9.62E-04	-4.20	2.81E-09	0.14	7.16E-01
ENSMUST00000238384	Rian-235	antisense	-0.86	4.90E-02	-3.41	8.26E-04	0.00	9.98E-01
ENSMUST00000180876	Rian-201	antisense	-0.85	1.81E-02	-3.29	6.97E-06	-0.33	4.62E-01
ENSMUST00000238689	Rian-259	antisense	-1.25	8.85E-04	-3.23	8.24E-07	0.32	4.20E-01
ENSMUST00000156275	4933427J07Rik-201	antisense	-2.86	1.37E-04	-3.10	3.56E-03	-0.78	3.59E-01
ENSMUST00000182406	Rian-209	antisense	-1.36	5.09E-04	-3.09	1.91E-07	-0.30	4.88E-01
ENSMUST00000238736	Rian-265	antisense	-0.94	7.48E-03	-2.99	1.65E-03	-0.53	4.04E-01
ENSMUST00000220250	Gm47628-201	processed_pseudogene	-2.67	7.37E-03	-2.99	5.71E-03		
ENSMUST00000182737	Rian-211	antisense	-0.72	3.52E-02	-2.77	1.56E-03	-0.80	2.09E-01
ENSMUST00000222638	Gm48541-201	processed_pseudogene	-1.52	9.62E-03	-2.73	1.84E-03	0.50	6.09E-01
ENSMUST00000223414	Gm48260-201	processed_pseudogene	-1.83	1.25E-02	-2.61	1.58E-02		
ENSMUST00000202316	Gm42785-201	processed_pseudogene	-2.41	5.61E-04	-2.58	4.34E-03	0.24	7.86E-01
ENSMUST00000221304	Gm47406-201	processed_pseudogene	-2.67	6.25E-03	-2.57	3.29E-02		
ENSMUST00000238357	Rian-233	antisense	-0.86	3.64E-02	-2.54	1.20E-02	-1.18	6.93E-02
ENSMUST00000219963	Gm47056-201	processed_pseudogene	-1.41	1.11E-02	-2.51	9.19E-03		
ENSMUST00000218052	Gm48793-201	processed_pseudogene	-1.78	3.63E-03	-2.51	3.76E-03		
ENSMUST00000221482	Gm48111-201	processed_pseudogene	-2.74	9.90E-06	-2.46	4.16E-03	-0.90	3.57E-01
ENSMUST00000222020	Gm48501-201	processed_pseudogene	-2.91	2.81E-06	-2.37	6.94E-03	-1.06	2.58E-01
ENSMUST00000219916	Gm47626-201	processed_pseudogene	-2.26	5.22E-05	-2.35	6.59E-03	-1.02	1.88E-01
ENSMUST00000213426	Gm47103-201	processed_pseudogene	-2.77	2.33E-04	-2.28	4.16E-02	0.11	9.05E-01
ENSMUST00000209049	Gm44715-201	processed_pseudogene	-2.01	6.24E-03	-2.24	4.09E-03		
ENSMUST00000222999	Gm48416-201	processed_pseudogene	-1.90	1.64E-03	-2.19	1.75E-02	-0.58	5.97E-01
ENSMUST00000149830	E130307A14Rik-209	antisense	-2.26	8.04E-05	-2.12	6.98E-03	-0.67	4.57E-01
ENSMUST00000222959	Gm47578-201	antisense	-2.43	7.33E-09	-2.12	7.12E-05	-0.21	5.99E-01
ENSMUST00000220994	Gm48119-201	processed_pseudogene	-2.56	1.30E-04	-2.11	2.73E-02	-0.29	7.40E-01
ENSMUST00000219753	Gm48132-201	processed_pseudogene	-1.93	5.06E-04	-2.06	5.55E-03	1.02	2.91E-01
ENSMUST00000200624	Gm42927-201	processed_pseudogene	-2.18	3.80E-04	-2.06	2.37E-03	0.00	9.95E-01
ENSMUST00000214349	Gm47356-201	processed_pseudogene	-1.81	5.78E-03	-2.04	1.51E-02	0.27	7.42E-01
ENSMUST00000219080	Gm47611-201	processed_pseudogene	-2.04	4.11E-04	-2.03	1.10E-02	-0.49	6.03E-01
ENSMUST00000218660	Gm48270-201	processed_pseudogene	-2.29	3.84E-04	-2.03	3.25E-02		
ENSMUST00000222978	Gm48493-201	processed_pseudogene	-2.13	6.04E-05	-2.02	1.01E-02	-0.20	8.07E-01
ENSMUST00000220734	Gm48365-201	processed_pseudogene	-1.63	8.99E-03	-1.98	1.51E-02	-0.54	5.29E-01
ENSMUST00000215114	Gm47102-201	processed_pseudogene	-1.72	6.52E-03	-1.96	1.12E-02	-0.13	8.72E-01
ENSMUST00000220577	Gm48498-201	processed_pseudogene	-2.05	2.79E-03	-1.95	1.79E-02	0.00	9.98E-01
ENSMUST00000196320	Gm43540-201	processed_pseudogene	-2.47	7.10E-04	-1.93	1.23E-02		
ENSMUST00000207537	Gm45038-201	processed_pseudogene	-2.60	1.07E-05	-1.93	1.85E-02	-0.37	6.85E-01
ENSMUST00000223888	Gm48765-201	antisense	-2.23	1.91E-07	-1.90	1.43E-03	-0.02	9.67E-01
ENSMUST00000215904	Gm48401-201	processed_pseudogene	-2.41	8.89E-05	-1.86	1.15E-02	-0.53	5.62E-01
ENSMUST00000218126	Gm48269-201	processed_pseudogene	-2.48	1.61E-03	-1.86	3.88E-02	1.17	2.42E-01
ENSMUST00000213142	Gm47106-201	antisense	-1.87	1.37E-05	-1.83	9.51E-03	-1.11	6.76E-02
ENSMUST00000228191	Gm49273-201	processed_pseudogene	-1.99	2.92E-03	-1.83	4.90E-02	-0.23	8.09E-01
ENSMUST00000218505	Gm47632-201	processed_pseudogene	-2.27	9.84E-04	-1.83	2.57E-02	-0.50	6.14E-01
ENSMUST00000216568	Gm48443-201	processed_pseudogene	-2.80	1.85E-04	-1.82	1.48E-02	0.18	8.51E-01
ENSMUST00000193321	Gm37515-201	processed_pseudogene	-1.90	1.28E-03	-1.80	7.48E-03		
ENSMUST00000044500	Gm4117-201	antisense	-1.77	3.30E-06	-1.63	1.71E-04	-0.48	3.11E-01
ENSMUST00000137603	4930511M06Rik-203	antisense	-2.17	1.83E-03	-1.53	4.12E-02	-0.62	5.10E-01
ENSMUST00000215393	Gm48128-201	processed_pseudogene	-2.04	1.57E-04	-1.47	4.12E-02	-0.08	9.21E-01
ENSMUST00000207795	Gm45792-201	antisense	-1.92	8.72E-05	-1.42	2.90E-02	-0.09	9.06E-01

Table S4B

id	log2FC brain astrocytes	p value brain astrocytes	log2FC brain microglia	p value brain microglia
Mus_musculus_tRNA-Ala-AGC-10-1	1.03	2.411E-08	0.91	0.001039
Mus_musculus_tRNA-Ala-AGC-2-2	0.42	0.027124	0.82	0.0280327
Mus_musculus_tRNA-Ala-AGC-5-1	0.85	0.0003674	0.95	0.0005043
Mus_musculus_tRNA-Ala-AGC-5-2	0.75	0.0029394	0.89	0.0001789
Mus_musculus_tRNA-Ala-AGC-5-3	0.73	0.0015867	0.93	0.0007649
Mus_musculus_tRNA-Ala-AGC-7-1	0.65	0.0005712	0.80	0.0091572
Mus_musculus_tRNA-Arg-ACG-3-1	0.40	0.0488495	1.13	0.0088205
Mus_musculus_tRNA-Arg-ACG-3-2	0.42	0.033223	1.04	0.0209114
Mus_musculus_tRNA-Arg-CCG-1-1	0.64	0.004102	1.11	0.0026875
Mus_musculus_tRNA-Arg-CCG-2-1	0.61	0.0034402	1.04	0.0266396
Mus_musculus_tRNA-Arg-CCG-3-1	0.90	8.888E-12	1.03	0.0242049
Mus_musculus_tRNA-Arg-CCT-1-1	0.53	0.0040011	1.05	0.0121269
Mus_musculus_tRNA-Arg-CCT-2-1	0.47	0.0211314	0.93	0.0329211
Mus_musculus_tRNA-Arg-CCT-2-2	0.48	0.0134195	0.93	0.0368435
Mus_musculus_tRNA-Arg-CCT-4-1	0.70	1.234E-07	1.10	0.0188261
Mus_musculus_tRNA-Arg-TCG-1-1	0.64	0.0024888	0.97	0.0496991
Mus_musculus_tRNA-Arg-TCG-2-1	0.49	0.014714	1.01	0.0401273
Mus_musculus_tRNA-Arg-TCG-3-2	0.53	0.0085892	0.90	0.0346345
Mus_musculus_tRNA-Arg-TCT-1-1	0.52	0.0015162	1.00	0.0210675
Mus_musculus_tRNA-Arg-TCT-2-1	0.87	0.0062088	0.99	0.0127326
Mus_musculus_tRNA-Arg-TCT-3-1	0.92	2.99E-06	0.98	0.0182948
Mus_musculus_tRNA-Asn-GTT-3-1	0.55	0.0013384	1.07	0.0195241
Mus_musculus_tRNA-Asn-GTT-3-2	0.34	0.0472405	1.23	0.0044805
Mus_musculus_tRNA-Asn-GTT-3-3	0.42	0.037483	1.08	0.012592
Mus_musculus_tRNA-Asn-GTT-3-4	0.55	0.0159117	1.15	0.0071226
Mus_musculus_tRNA-Asn-GTT-3-5	0.58	0.0038215	1.14	0.0105368
Mus_musculus_tRNA-Asn-GTT-3-6	0.51	0.0032007	1.10	0.0145453
Mus_musculus_tRNA-Asn-GTT-3-7	0.54	0.0050336	1.04	0.0109743
Mus_musculus_tRNA-Asn-GTT-3-8	0.56	0.004281	1.14	0.0079783
Mus_musculus_tRNA-Asn-GTT-3-9	0.51	0.0034049	1.08	0.0110947
Mus_musculus_tRNA-Asp-GTC-1-1	0.32	0.0220293	1.00	0.0127113
Mus_musculus_tRNA-Asp-GTC-1-11	0.33	0.0269528	0.99	0.0111385
Mus_musculus_tRNA-Asp-GTC-1-12	0.30	0.0227813	1.01	0.01075
Mus_musculus_tRNA-Asp-GTC-1-13	0.31	0.0384566	1.03	0.0095399
Mus_musculus_tRNA-Asp-GTC-1-2	0.31	0.027046	1.02	0.0098384
Mus_musculus_tRNA-Asp-GTC-1-3	0.29	0.0417782	0.98	0.0123366
Mus_musculus_tRNA-Asp-GTC-1-4	0.34	0.0129838	0.99	0.013356
Mus_musculus_tRNA-Asp-GTC-1-5	0.29	0.0404633	1.00	0.0108294
Mus_musculus_tRNA-Asp-GTC-2-1	0.48	0.0007895	0.91	0.0279327
Mus_musculus_tRNA-Gln-CTG-4-1	0.61	0.0052039	1.32	0.006339
Mus_musculus_tRNA-Gln-TTG-3-2	0.53	0.0021239	1.20	0.0329279
Mus_musculus_tRNA-Glu-CTC-1-1	0.53	0.0050034	0.67	0.0224666
Mus_musculus_tRNA-Glu-CTC-1-2	0.55	0.0043696	0.69	0.0179312
Mus_musculus_tRNA-Glu-CTC-1-3	0.54	0.0033434	0.70	0.0181159
Mus_musculus_tRNA-Glu-CTC-1-4	0.56	0.0021174	0.67	0.0216678
Mus_musculus_tRNA-Glu-CTC-1-5	0.58	0.0020032	0.70	0.0155478
Mus_musculus_tRNA-Glu-CTC-1-6	0.55	0.0037964	0.67	0.0212789
Mus_musculus_tRNA-Glu-CTC-1-7	0.56	0.0021633	0.70	0.0150743
Mus_musculus_tRNA-Glu-CTC-1-8	0.58	0.0029873	0.67	0.0259373

id	log2FC brain astrocytes	p value brain astrocytes	log2FC brain microglia	p value brain microglia
Mus_musculus_tRNA-Glu-CTC-1-9	0.59	0.0017285	0.66	0.0229196
Mus_musculus_tRNA-Glu-TTC-1-1	0.33	0.0422164	0.54	0.0343054
Mus_musculus_tRNA-Glu-TTC-3-2	1.16	1.091E-10	0.96	0.0268135
Mus_musculus_tRNA-Ile-AAT-1-1	0.89	5.439E-06	0.88	0.0147309
Mus_musculus_tRNA-Ile-AAT-1-2	0.83	0.000407	1.00	0.0087905
Mus_musculus_tRNA-Ile-AAT-1-3	0.93	6.981E-06	0.97	0.0045454
Mus_musculus_tRNA-Ile-AAT-1-4	0.90	8.747E-06	0.82	0.0304855
Mus_musculus_tRNA-Ile-AAT-1-5	0.90	7.233E-06	0.91	0.0092935
Mus_musculus_tRNA-Ile-AAT-1-6	0.88	2.229E-05	0.92	0.0075638
Mus_musculus_tRNA-Ile-AAT-1-7	0.99	4.718E-07	0.93	0.0051844
Mus_musculus_tRNA-Ile-AAT-1-8	0.97	0.0002543	0.93	0.0057392
Mus_musculus_tRNA-Ile-AAT-2-1	0.70	0.0005787	1.04	0.0004148
Mus_musculus_tRNA-Ile-AAT-3-1	1.12	2.992E-05	1.27	0.0104593
Mus_musculus_tRNA-Ile-AAT-4-1	1.29	7.274E-08	1.43	6.699E-05
Mus_musculus_tRNA-Leu-AAG-2-1	0.53	0.0037461	0.99	0.0323406
Mus_musculus_tRNA-Leu-AAG-3-1	0.62	0.0009199	1.02	0.0290566
Mus_musculus_tRNA-Leu-TAG-3-1	0.36	0.0185379	0.80	0.0063725
Mus_musculus_tRNA-Lys-CTT-2-1	0.80	1.414E-07	1.08	0.031782
Mus_musculus_tRNA-Lys-CTT-2-2	0.81	4.15E-08	1.08	0.0307445
Mus_musculus_tRNA-Lys-CTT-3-1	0.63	2.214E-06	0.98	0.0322216
Mus_musculus_tRNA-Lys-CTT-3-2	0.70	3.701E-07	0.95	0.0399091
Mus_musculus_tRNA-Lys-CTT-3-3	0.65	5.915E-06	1.04	0.0243016
Mus_musculus_tRNA-Lys-CTT-3-4	0.65	1.101E-06	1.00	0.0305654
Mus_musculus_tRNA-Lys-CTT-3-5	0.65	3.052E-06	0.98	0.0351305
Mus_musculus_tRNA-Lys-CTT-3-6	0.67	3.05E-07	1.00	0.0342803
Mus_musculus_tRNA-Lys-CTT-3-7	0.70	2.363E-07	1.00	0.0310031
Mus_musculus_tRNA-Lys-TTT-1-1	0.74	0.0001412	0.48	0.0408234
Mus_musculus_tRNA-Lys-TTT-1-4	0.79	0.0001324	0.49	0.0421978
Mus_musculus_tRNA-Lys-TTT-1-6	0.80	0.0001745	0.46	0.0473374
Mus_musculus_tRNA-Met-CAT-1-1	0.60	0.0064072	1.06	0.0021259
Mus_musculus_tRNA-Met-CAT-1-2	0.65	0.0020914	1.07	0.0020441
Mus_musculus_tRNA-Met-CAT-2-1	0.54	0.0095566	0.87	0.008732
Mus_musculus_tRNA-Met-CAT-2-2	0.61	0.0026377	0.98	0.0044415
Mus_musculus_tRNA-Pro-AGG-1-5	0.34	0.0265007	0.87	0.0355721
Mus_musculus_tRNA-Pro-CGG-1-2	0.31	0.032938	0.90	0.0442809
Mus_musculus_tRNA-Ser-CGA-1-1	0.50	0.0014438	1.04	0.0200069
Mus_musculus_tRNA-Ser-CGA-2-1	0.39	0.0331196	1.06	0.0048253
Mus_musculus_tRNA-Ser-GCT-1-1	0.38	0.0257437	1.12	0.0056916
Mus_musculus_tRNA-Ser-GCT-4-1	0.33	0.0383137	1.15	0.0021673
Mus_musculus_tRNA-Thr-AGT-1-1	1.13	2.373E-11	1.29	0.0170938
Mus_musculus_tRNA-Thr-AGT-1-2	1.10	6.88E-12	1.21	0.0122424
Mus_musculus_tRNA-Thr-AGT-2-1	1.14	1.238E-15	1.48	0.006952
Mus_musculus_tRNA-Thr-AGT-3-1	1.36	4.402E-12	1.44	0.0044427
Mus_musculus_tRNA-Thr-AGT-4-1	0.92	2.079E-06	1.41	0.0025291
Mus_musculus_tRNA-Thr-CGT-1-1	0.83	1.032E-07	1.42	0.0071638
Mus_musculus_tRNA-Thr-TGT-2-1	1.07	1.239E-10	1.18	0.0057898
Mus_musculus_tRNA-Thr-TGT-3-1	0.93	4.486E-08	0.93	0.0361311
Mus_musculus_tRNA-Thr-TGT-3-2	0.87	7.52E-07	0.97	0.0296215
Mus_musculus_tRNA-Trp-CCA-5-1	0.50	0.0147281	0.94	0.0183402

Table S5A

id	gene symbol	log2FC brain RNAseq	p value brain RNAseq	log2FC brain astrocytes	p value brain astrocytes	log2FC brain microglia	p value brain microglia	log2FC brain neurons	p value brain neurons
ENSMUSG00000029816	Gpnmb	1.16	8.2015E-05			4.81	5.1275E-10		
ENSMUSG00000068129	Cst7	0.05	0.020068			3.64	1.0878E-07		
ENSMUSG00000119030	Rnu1b2	0.00	0.65345285			3.40	1.3719E-05	0.00	0.42042664
ENSMUSG00000030789	Itgax	0.04	0.02789338			3.32	5.7914E-09		
ENSMUSG00000029304	Spp1	0.03	0.05751935			3.21	3.1757E-06		
ENSMUSG00000002985	Apoe	0.03	0.06690812	-0.14	0.43082006	2.99	2.2604E-16	0.00	0.89246436
ENSMUSG00000079293	Clec7a	0.01	0.16699636			2.75	2.3677E-10		
ENSMUSG00000002602	Axl	0.03	0.06465426	-0.16	0.45820477	2.51	5.6869E-09		
ENSMUSG00000014599	Csf1	0.02	0.1343783	-0.12	0.57421907	1.82	1.1389E-06		
ENSMUSG00000025351	Cd63	0.02	0.14954943	0.12	0.5456425	1.61	3.4446E-06	0.00	0.76373585
ENSMUSG00000025854	Fam20c	0.02	0.28982543	-0.34	0.1136666	1.57	0.00011096		
ENSMUSG00000015568	Lpl	0.03	0.05815777			1.55	0.00020539		
ENSMUSG00000046805	Mpeg1	0.01	0.41907847			1.52	2.5956E-05		
ENSMUSG00000026825	Dnm1	-2.50	1.6294E-06	-2.59	3.3764E-08	-1.57	0.0002435		
ENSMUSG00000052727	Map1b	-0.17	0.0025592	-1.70	4.0261E-07	-1.59	0.00018426	0.00	0.25447664
ENSMUSG00000017390	Aldoc	-0.01	0.52842589	-0.90	3.3646E-06	-1.60	0.00019204		
ENSMUSG00000096054	Syne1	-0.01	0.23239481	-0.71	0.00022228	-1.74	9.6448E-05	0.00	0.7818642
ENSMUST00000083637	Mir326	-0.59	0.00025825	-1.99	6.6141E-08	-1.77	2.8742E-05	0.00	0.63068611
ENSMUSG00000022565	Plec	-0.02	0.24985857	-0.28	0.1685346	-1.77	5.7877E-05	0.00	0.7908705
ENSMUSG00000053310	Nrgn	-0.65	0.00043403	-1.06	0.00050302	-1.79	0.00019615		
ENSMUSG00000072214	Septin5	-0.03	0.05914792	-1.12	0.00014447	-1.80	0.00015558	0.00	0.54717314
ENSMUST00000083511	Mir143	-0.04	0.04479214	-0.14	0.43461938	-1.89	0.00011676		
ENSMUSG00000034730	Adgrb1	-0.02	0.20198473	-0.74	0.00031832	-1.97	0.00015476	0.00	0.87947925
ENSMUSG00000030302	Atp2b2	-0.12	0.00518674	-1.32	3.611E-06	-2.07	9.2401E-05	0.00	0.78495942
ENSMUST00000238689	Rian	-0.02	0.11234544	-0.41	0.05311974	-2.08	0.00012091	0.00	0.96355139
ENSMUST00000083815	AF357428	-0.04	0.03318791	-0.55	0.01091435	-2.16	0.00010756	0.00	0.62940967
ENSMUSG00000005089	Slc1a2	-0.01	0.21983712	-1.27	2.4259E-10	-2.18	0.00010591		
ENSMUSG00000057897	Camk2b	-0.96	0.00021332	-2.40	3.979E-07	-2.25	8.3875E-05		
ENSMUST00000082553	Gm23736	0.00	0.88168465	-0.54	0.01497952	-2.33	8.7192E-05	0.00	0.95788018
ENSMUST00000082651	Gm25129	-0.01	0.23867131	-0.45	0.03199195	-2.36	2.6744E-05	0.00	0.13333355
ENSMUSG00000027254	Map1a	-0.02	0.15237382	-0.53	0.01036048	-2.50	1.5953E-05	0.00	0.49889408
ENSMUST00000182406	Rian	-0.02	0.10394942	-0.45	0.04125228	-2.55	5.5232E-07	0.00	0.44471351
ENSMUSG00000032589	Bsn	-2.02	1.5944E-06	-1.28	0.00020451	-2.67	3.4678E-06	0.00	0.28112481
ENSMUST00000180876	Rian	-0.02	0.18365675	-0.35	0.09760828	-2.67	1.3291E-05	0.00	0.53067915
ENSMUST00020183598	AF357425	-0.02	0.11334146	-0.42	0.05507834	-3.07	2.2246E-08	0.00	0.68429085
ENSMUSG00000067889	Sptbn2	-2.38	4.0164E-07	-2.52	1.5121E-07	-3.12	5.3959E-06	0.00	0.54203521
ENSMUST00000082687	Gm26922	-0.02	0.13704364	-0.42	0.05101131	-3.28	1.3526E-05	0.00	0.8731486
ENSMUSG00000036564	Ndrg4	-0.83	0.00028306	-1.95	3.7696E-07	-3.91	1.7194E-06		

id<br>Table S5B	gene symbol	log2FC brain RNAseq	p value brain RNAseq	log2FC brain astrocytes	p value brain astrocytes	log2FC brain microglia	p value brain microglia	log2FC brain neurons	p value brain neurons
ENSMUST00000243949	Gm54109	0.01	1.96E-01	1.47	1.97E-06				
ENSMUSG00000021136	Smoc1	0.00	9.45E-01	1.26	1.39E-04				
ENSMUSG00000033595	Lgi3	0.01	2.98E-01	1.23	2.76E-09				
ENSMUSG00000026051	Ecrg4	0.03	8.81E-02	1.05	3.93E-04			0.00	9.96E-01
ENSMUSG00000026728	Vim	0.16	3.02E-03	1.05	2.31E-04	0.00	3.58E-01	0.00	4.05E-02
ENSMUSG00000064215	Ifi27	0.03	9.26E-02	1.04	4.21E-05	0.00	3.67E-01	0.00	5.45E-01
ENSMUST00000159669	C4a	0.02	1.06E-01	1.03	3.09E-04	0.00	6.31E-01		
ENSMUST00000157683	Gm24507	0.08	1.20E-02	1.03	4.22E-08	0.00	6.38E-01	0.00	8.54E-01
ENSMUSG00000001025	S100a6	0.04	5.23E-02	1.00	8.44E-05	0.00	6.46E-01	0.00	7.84E-01
ENSMUSG00000020734	Grin2c	-0.02	9.57E-02	-1.51	2.54E-08	-0.11	1.74E-02		
ENSMUST00000246023	Gm11789	-0.02	2.32E-01	-1.52	8.76E-05	0.00	2.44E-01	0.00	9.06E-01
ENSMUSG00000006205	Htra1	-0.01	4.19E-01	-1.52	6.77E-11				
ENSMUST00000142374	Gm14236	-0.19	2.21E-03	-1.52	4.85E-08	-0.01	8.34E-02	0.00	4.10E-01
ENSMUST00000201142	1700028E10Rik	-0.01	4.32E-01	-1.52	2.56E-05	0.00	7.38E-01	0.00	2.62E-01
ENSMUSG00000029608	Rph3a	-2.70	2.34E-06	-1.54	6.50E-05	-0.28	4.96E-03		
ENSMUST00000093527	Gm47289	-0.01	4.51E-01	-1.54	7.87E-05	0.00	2.94E-01	0.00	7.03E-01
ENSMUST00000132470	Gm11261	-0.03	1.67E-01	-1.56	3.44E-05	0.00	3.43E-01	0.00	7.07E-01
ENSMUSG00000039809	Gabbr2	-0.06	1.66E-02	-1.57	3.73E-06	-1.17	3.89E-04		
ENSMUSG00000025582	Nptx1	-0.28	1.65E-03	-1.59	6.43E-05	-0.10	1.94E-02		
ENSMUST00000140645	Gm11789	-0.02	2.23E-01	-1.62	6.10E-05	0.00	4.40E-01	0.00	8.43E-01
ENSMUSG00000023033	Scn8a	-0.04	3.46E-02	-1.64	1.91E-05	-0.16	1.07E-02	0.00	4.27E-01
ENSMUSG00000052727	Map1b	-0.17	2.56E-03	-1.70	4.03E-07	-1.59	1.84E-04	0.00	2.54E-01
ENSMUSG00000019194	Scn1b	-0.03	8.50E-02	-1.71	1.95E-05	0.00	4.28E-01		
ENSMUSG00000019232	Etnppl	-0.03	4.17E-02	-1.76	3.01E-05				
ENSMUST00000181667	Gm805	-0.04	4.73E-02	-1.76	2.17E-06	0.00	8.91E-01	0.00	9.20E-01
ENSMUSG00000086411	Gm14236	-0.10	5.84E-03	-1.76	2.41E-16	-0.03	2.06E-02	0.00	7.47E-01
ENSMUSG00000016349	Eef1a2	-1.94	2.24E-05	-1.80	7.11E-06	-1.42	3.28E-04		
ENSMUSG00000040907	Atp1a3	-0.61	3.30E-04	-1.82	1.34E-06	-0.08	1.40E-02	0.00	5.24E-01
ENSMUSG00000074657	Kif5a	-1.43	8.74E-05	-1.84	3.00E-07	-1.34	3.96E-04	-0.01	1.83E-02
ENSMUSG00000070570	Slc17a7	-1.52	7.02E-05	-1.85	1.56E-05	-0.65	1.46E-03	0.00	2.17E-01
ENSMUSG00000024617	Camk2a	-1.80	2.58E-05	-1.86	9.37E-06	-0.39	2.46E-03		
ENSMUST00000138928	1110018N20Rik	-0.03	9.28E-02	-1.88	2.95E-07	0.00	8.65E-01	0.00	6.74E-01
ENSMUST00000181762	Gm26638	-0.01	3.40E-01	-1.89	1.30E-05	0.00	7.42E-01	0.00	6.24E-01
ENSMUSG00000036564	Ndrg4	-0.83	2.83E-04	-1.95	3.77E-07	-3.91	1.72E-06		
ENSMUSG00000062296	Trank1	-0.17	3.22E-03	-1.96	1.18E-05	-0.08	2.89E-02		
ENSMUST00000181420	0610038B21Rik	-0.03	1.17E-01	-1.96	7.04E-08	0.00	4.64E-01	0.00	9.97E-01
ENSMUST00000211689	Gm6145	-0.03	1.41E-01	-1.96	6.36E-06	0.00	5.59E-01	0.00	5.20E-01
ENSMUSG00000034799	Unc13a	-0.74	2.26E-04	-1.97	6.47E-06	-0.06	2.39E-02	0.00	4.29E-01
ENSMUST00000083637	Mir326	-0.59	2.58E-04	-1.99	6.61E-08	-1.77	2.87E-05	0.00	6.31E-01
ENSMUST00000246482	Gm14207	-0.06	1.46E-02	-2.02	7.01E-06	0.00	6.89E-01		
ENSMUSG00000027273	Snap25	-1.79	2.61E-05	-2.06	1.05E-06	-0.75	1.10E-03		
ENSMUSG00000026458	Ppfia4	0.00	7.50E-01	-2.07	1.14E-05	0.00	8.35E-01		
ENSMUSG00000038244	Mical2	-0.35	1.15E-03	-2.07	8.76E-08	-0.11	1.49E-02		
ENSMUSG00000025867	Cplx2	-0.65	3.66E-04	-2.08	1.80E-06	-1.40	3.17E-04	0.00	6.64E-01
ENSMUST00000147446	1110018N20Rik	-0.02	2.18E-01	-2.12	5.16E-08	0.00	3.74E-01	0.00	5.27E-01
ENSMUSG00000023473	Celsr3	-0.04	3.15E-02	-2.12	7.55E-06	-0.06	3.44E-02	0.00	8.95E-01
ENSMUSG00000017740	Slc12a5	-2.01	2.99E-05	-2.14	3.20E-06	-1.50	3.15E-04		
ENSMUSG00000031980	Agt	-0.01	2.47E-01	-2.15	3.93E-14				
ENSMUST00000216190	Gm36799	-0.03	1.50E-01	-2.16	1.50E-07	0.00	4.88E-01	0.00	3.52E-01
ENSMUSG00000016346	Kcnq2	-2.13	4.46E-06	-2.18	6.44E-06	-0.41	2.88E-03	0.00	2.08E-01
ENSMUSG00000057897	Camk2b	-0.96	2.13E-04	-2.40	3.98E-07	-2.25	8.39E-05		
ENSMUSG00000033615	Cplx1	-2.93	1.49E-07	-2.46	1.08E-06	-1.49	2.16E-04	-0.02	4.98E-03
ENSMUSG00000020431	Adcy1	-0.48	7.29E-04	-2.50	2.45E-07	-0.75	1.17E-03		
ENSMUSG00000067889	Sptbn2	-2.38	4.02E-07	-2.52	1.51E-07	-3.12	5.40E-06	0.00	5.42E-01
ENSMUSG00000026825	Dnm1	-2.50	1.63E-06	-2.59	3.376E-08	-1.573387	2.43E-04		

Table S6

id	gene symbol	log2FC brain RNAseq	p value brain RNAseq	log2FC brain astrocytes	p value brain astrocytes
**ENSMUST00000240501**	Rnu1a1	0.02	6.32E-01	0.42	1.30E-02
**ENSMUSG00000119476**	Rnu1a1	0.00	7.70E-01	0.42	5.42E-02
**ENSMUST00001239531**	Rnu1b1	0.00	9.07E-01	0.32	5.38E-02
**ENSMUSG00000118876**	Rnu1b1				
**ENSMUST00000240438**	Rnu1b2	-0.01	8.83E-01	0.32	5.49E-02
**ENSMUSG00000119030**	Rnu1b2	0.00	6.53E-01		
**ENSMUST00000240510**	Rnu1b6	0.01	6.48E-01	0.39	3.28E-02
**ENSMUSG00000118677**	Rnu1b6	0.00	9.89E-01	0.14	5.03E-01
**ENSMUST00000240231**	Rnu2-10	0.06	1.66E-01	0.43	9.72E-03
**ENSMUSG00000118837**	Rnu2-10	-0.01	4.26E-01	-0.34	2.50E-02
**ENSMUST00000116892**	Rnu4atac	0.01	7.13E-01	0.29	9.69E-02
**ENSMUSG00000080542**	Rnu4atac	0.00	8.71E-01	-0.06	7.87E-01
**ENSMUST00000101817**	Rnu5g	-0.01	6.98E-01	-0.16	3.76E-01
**ENSMUSG00000119286**	Rnu5g	-0.04	1.67E-01	-0.37	6.65E-02
**ENSMUST00000240167**	Rnu6	0.02	5.04E-01	0.56	1.33E-03
**ENSMUSG00000119272**	Rnu6				
**ENSMUST00000082808**	Rnu6-ps1	0.02	3.84E-01	0.14	4.91E-01
**ENSMUSG00000064742**	Rnu6-ps1				
**ENSMUSG00000119323**	Rnu6-ps2				
**ENSMUST00000178059**	Rnu6-ps2				
**ENSMUST00000158458**	Rnu7	0.00	8.68E-01	0.13	4.86E-01
**ENSMUSG00000089083**	Rnu7	0.00	9.60E-01		
**ENSMUSG00000089468**	Rnu7-ps1				
**ENSMUST00000158843**	Rnu7-ps1				
**ENSMUSG00000088970**	Rnu7-ps3				
**ENSMUST00000158345**	Rnu7-ps3				
**ENSMUST00000104135**	Rnu11	0.05	2.62E-01	0.33	5.53E-02
**ENSMUSG00000077323**	Rnu11	0.01	7.92E-01	0.16	4.18E-01
**ENSMUST00000083242**	Rnu12	0.07	1.01E-01	0.44	1.00E-02
**ENSMUSG00000065176**	Rnu12	0.00	9.02E-01	0.22	2.30E-01

log2FC brain microglia	p value brain microglia	log2FC brain neurons	p value brain neurons
0.00	3.53E-01	0.00	9.11E-01
0.00	6.71E-01		
0.00	2.14E-01	0.00	9.48E-01
0.00	2.15E-01	0.00	8.90E-01
3.40	1.37E-05	0.00	4.20E-01
0.00	7.41E-01	0.00	8.32E-01
-0.01	1.53E-01	0.00	4.47E-01
0.00	5.60E-01	0.00	3.72E-01
0.00	7.72E-01	0.00	
0.00	9.71E-01	0.00	8.82E-01
0.00	7.88E-01	0.00	6.45E-01
0.00	5.96E-01	0.00	1.09E-01
0.00	3.40E-01	0.00	6.82E-01
0.00	5.14E-01	0.00	8.54E-01
0.00	9.28E-01	0.00	5.55E-01
0.00	6.98E-01	0.00	4.20E-01
0.00	4.24E-01		
0.00	6.72E-01	0.00	9.95E-01
0.00	9.99E-01	0.00	7.02E-01
0.00	9.07E-01	0.00	9.21E-01
0.00	4.85E-01	0.00	5.60E-01

MouseBrain

GenXPro GmbH

2026-03-23

Table of Contents

1 Materials and Methods

2 Results

2.1 Quality Control

2.2 General Results

2.2.1 Expression Quantification

2.3 KIN vs. WT

2.3.1 Differential Expression Analysis (DEA)

2.3.2 Gene Set Enrichment Analysis (GSEA)

2.3.2.1 Reactome

2.3.2.2 GO-MF

2.3.2.3 GO-BP

2.3.2.4 GO-CC

2.4 Astrocytes_KIN vs. Astrocytes_WT

2.4.1 Differential Expression Analysis (DEA)

2.4.2 Gene Set Enrichment Analysis (GSEA)

2.4.2.1 Reactome

2.4.2.2 GO-MF

2.4.2.3 GO-BP

2.4.2.4 GO-CC

2.5 Microglia_KIN vs. Microglia_WT

2.5.1 Differential Expression Analysis (DEA)

2.5.2 Gene Set Enrichment Analysis (GSEA)

2.5.2.1 GO-MF

2.5.2.2 GO-BP

2.5.2.3 GO-CC

2.6 Neurons_KIN vs. Neurons_WT

2.6.1 Gene Set Enrichment Analysis (GSEA)

2.6.1.1 Reactome

2.6.1.2 GO-MF

2.6.1.3 GO-BP

2.6.1.4 GO-CC

2.7 References

Materials and Methods

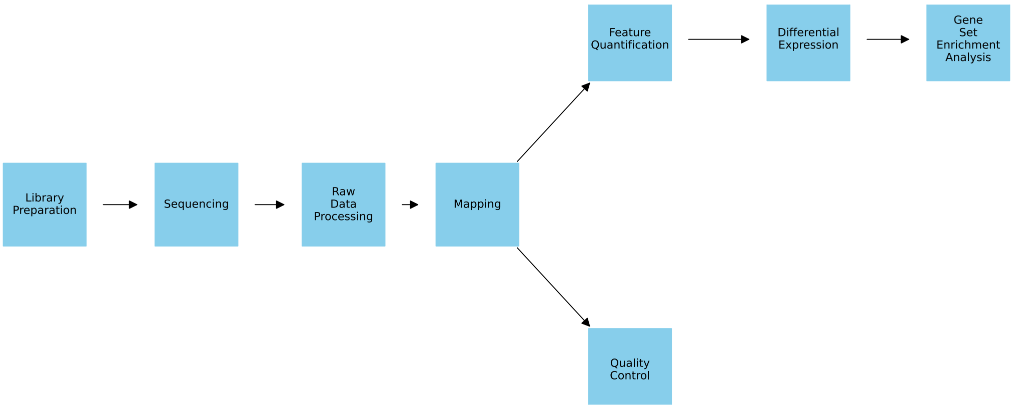

Figure 1. Bioinformatics Pipeline

Library Preparation and Sequencing

Library Preparation

PATS__A, panRNA-Seq

Details of the library preparation have not been provided.

Sequencing

PATS__A, panRNA-Seq

Sequencing was performed on an Illumina NextSeq 500 instrument with 1x76 bps.

Data Processing

Raw Data Processing

Raw sequencing reads were processed by removing adapter sequences, low-quality bases, and technical artifacts through trimming. For UMI-containing libraries, PCR duplicates were eliminated by collapsing reads with identical UMIs and insert sequences. The resulting high-quality, deduplicated reads were used for downstream analyses. Unprocessed sequencing reads were adapter-trimmed and quality-trimmed using *Cutadapt* 4.6 [1]. UMI deduplication was done using in-house tools collapsing PCR duplicates using UMIs into a single read. Also using *Cutadapt*, additional sequencing artifacts were removed from the reads in an extra cleaning step. *FastQC* 0.11.9 [2] was used to assess the quality of sequencing reads.

PATS__A, panRNA-Seq

Cutadapt arguments for trimming:

`-m 14 -q 20 -n 8`

UMI deduplication, allowed mismatches per *N* bases UMI:

`8`

Cutadapt arguments for cleaning:

`-m 10 -u 4 -a 'A{300}X' -a 'A{10};o=10' -a 'T{10};o=10' -a 'C{10};o=10' -a 'G{10};o=10' -n 5`

Mapping

The processed sequencing reads were mapped using *Bowtie2* 2.4.4 [3].

mm39 Mus musculus

We used the following arguments for the specified reference:

mirna:

Sequences (FASTA):

mirna.fa.gz

Bowtie2 args:

`--sensitive --local`

pirna:

Sequences (FASTA):

pirna.fa.gz

Bowtie2 args:

`--sensitive --local`

trna:

Sequences (FASTA):

trna.fa.gz

Bowtie2 args:

`--sensitive --local`

ENSEMBL_ncrna:

Sequences (FASTA):

ENSEMBL_ncrna.fa.gz

Bowtie2 args:

`--sensitive --local`

ENSEMBL_dna:

Sequences (FASTA):

ENSEMBL_dna.fa.gz

Bowtie2 args:

`--sensitive --local`

Quality Control

MultiQC 1.32 [10] was used to create a single report visualising output from multiple tools across many samples, enabling global trends and biases to be quickly identified.

Data Analysis

Expression Quantification

The processd and mapped reads were used for gene expression quantification. Expression quantification was performed using *HTSeq* 2.0.2 [6]. Raw counts were geometric-mean-normalized using *DESeq2* 1.38 [5] and TPM-normalized by dividing each count with the sum of all counts for each sample multiplied by one million.

mm39, Mus musculus

Genes

We used the following arguments for the specified reference:

mirna:

Annotation (GTF/GFF):

mirna.gtf.gz

htseq-count args:

`-i transcript_id -r name -a 0 -m union -s no`

pirna:

Annotation (GTF/GFF):

pirna.gtf.gz

htseq-count args:

`-i transcript_id -r name -a 0 -m union -s no`

trna:

Annotation (GTF/GFF):

trna.gtf.gz

htseq-count args:

`-i transcript_id -r name -a 0 -m union -s no`

ENSEMBL_ncrna:

Annotation (GTF/GFF):

ENSEMBL_ncrna.gtf.gz

htseq-count args:

`-i transcript_id -r name -a 0 -m union -s no`

ENSEMBL_dna:

Annotation (GTF/GFF):

ENSEMBL_dna.gtf.gz

htseq-count args:

`-i gene_id -r pos -a 0 -s no`

Differential Expression Analysis (DEA)

In order to identify significant differences in the transcript- or gene abundance in the different comparisons, differential expression analysis (DEA) was performed using *DESeq2* 1.38 [5].

mm39, Mus musculus

Genes

Only entries having at least a raw read count of 5 in at least 2 samples were used in the DEA. Log2FoldChange values were shrinked using *ashr* [7]. DEA results with an FDR-adjusted p-value lesser or equal than 0.05 and an absolute log2Fold-Change greater or equal than 1.0 were called significant.

Gene Set Enrichment Analysis (GSEA)

Gene set enrichment analysis (GSEA) is a computational method used to determine whether predefined groups of genes, such as those involved in specific biological pathways or functions, show statistically significant differences in expression between two conditions. Instead of focusing on individual genes, GSEA evaluates gene sets collectively, increasing sensitivity and biological interpretability, especially when changes in single genes are modest but coordinated. This approach uses ranked gene expression data to assess whether members of a gene set are overrepresented at the extremes of the list. GSEA is widely used to identify affected pathways, infer functional mechanisms, and interpret results from high-throughput experiments like RNA-seq or microarrays. We used R and *clusterProfiler* [8] for gene set enrichment analysis and visualization.

mm39, Mus musculus

Genes

Using the reference database *org.Mm.eg.db* we analyzed gene sets from the following databases:

- Reactome
- GO-MF
- GO-BP
- GO-CC

Only gene sets having a minimum size of 5 and a maximum size of 500 were used for the analysis. P-values and FDR-values are based on 1000 permutations and we used a p-value cutoff of 0.05. WikiPathways images were colored and rendered using *CanvasXpress* [9].

Results

Quality Control

Table 1. QC results for 18 samples sequenced with panRNA-Seq.

Sample	Condition	Tissue	# Reads in total	% Reads mapped to pirna	% Reads mapped to trna	% Reads mapped to ENSEMBL_ncrna	% Reads mapped to ENSEMBL_dna	% mRNA alignments to ENSEMBL_dna	# Different genes counted
Microglia_WT_1	WT	Microglia_WT	14.002.145	10,86	3,33	33,01	5,90	1,86	25.151
Microglia_WT_2	WT	Microglia_WT	16.175.286	10,85	11,37	24,01	3,99	1,22	20.860
Microglia_WT_3	WT	Microglia_WT	11.182.080	11,61	3,71	30,78	5,59	1,60	23.132
Microglia_KIN_1	KIN	Microglia_KIN	16.103.916	9,65	14,46	21,81	4,51	1,41	22.770
Microglia_KIN_2	KIN	Microglia_KIN	12.878.630	10,23	7,10	28,32	5,59	1,88	22.997
Microglia_KIN_3	KIN	Microglia_KIN	13.787.093	10,10	6,43	28,93	5,60	1,73	23.624
Astrocytes_WT_1	WT	Astrocytes_WT	13.031.858	9,71	14,39	23,08	5,22	1,63	25.381
Astrocytes_WT_2	WT	Astrocytes_WT	15.286.790	8,77	16,61	21,64	4,76	1,64	25.032
Astrocytes_WT_3	WT	Astrocytes_WT	13.529.054	8,69	15,13	22,79	5,47	1,73	26.169
Astrocytes_KIN_1	KIN	Astrocytes_KIN	15.784.207	9,65	18,61	25,15	4,62	1,77	24.699
Astrocytes_KIN_2	KIN	Astrocytes_KIN	15.274.157	9,52	17,98	23,07	3,42	1,14	20.846
Astrocytes_KIN_3	KIN	Astrocytes_KIN	15.132.444	9,43	15,81	24,94	4,03	1,45	22.899
Neurons_WT_1	WT	Neurons_WT	11.613.218	3,74	7,11	9,37	1,92	0,47	15.290
Neurons_WT_2	WT	Neurons_WT	12.032.715	4,38	11,14	8,79	2,15	0,55	16.303
Neurons_WT_3	WT	Neurons_WT	10.943.546	3,81	6,10	7,15	1,47	0,37	12.575
Neurons_KIN_1	KIN	Neurons_KIN	14.125.002	7,12	12,48	13,87	2,95	0,73	20.094
Neurons_KIN_2	KIN	Neurons_KIN	12.863.002	6,22	11,45	11,95	2,75	0,65	18.498
Neurons_KIN_3	KIN	Neurons_KIN	14.641.280	7,54	13,18	14,61	2,77	0,80	19.757

As shown in Table 1, we studied 18 samples using panRNA-Seq. The samples had a median of 13894619 total reads. , and 1.26% of reads mapping to the reference genome. Additionally, on average 21448 genes detected per sample.

General Results

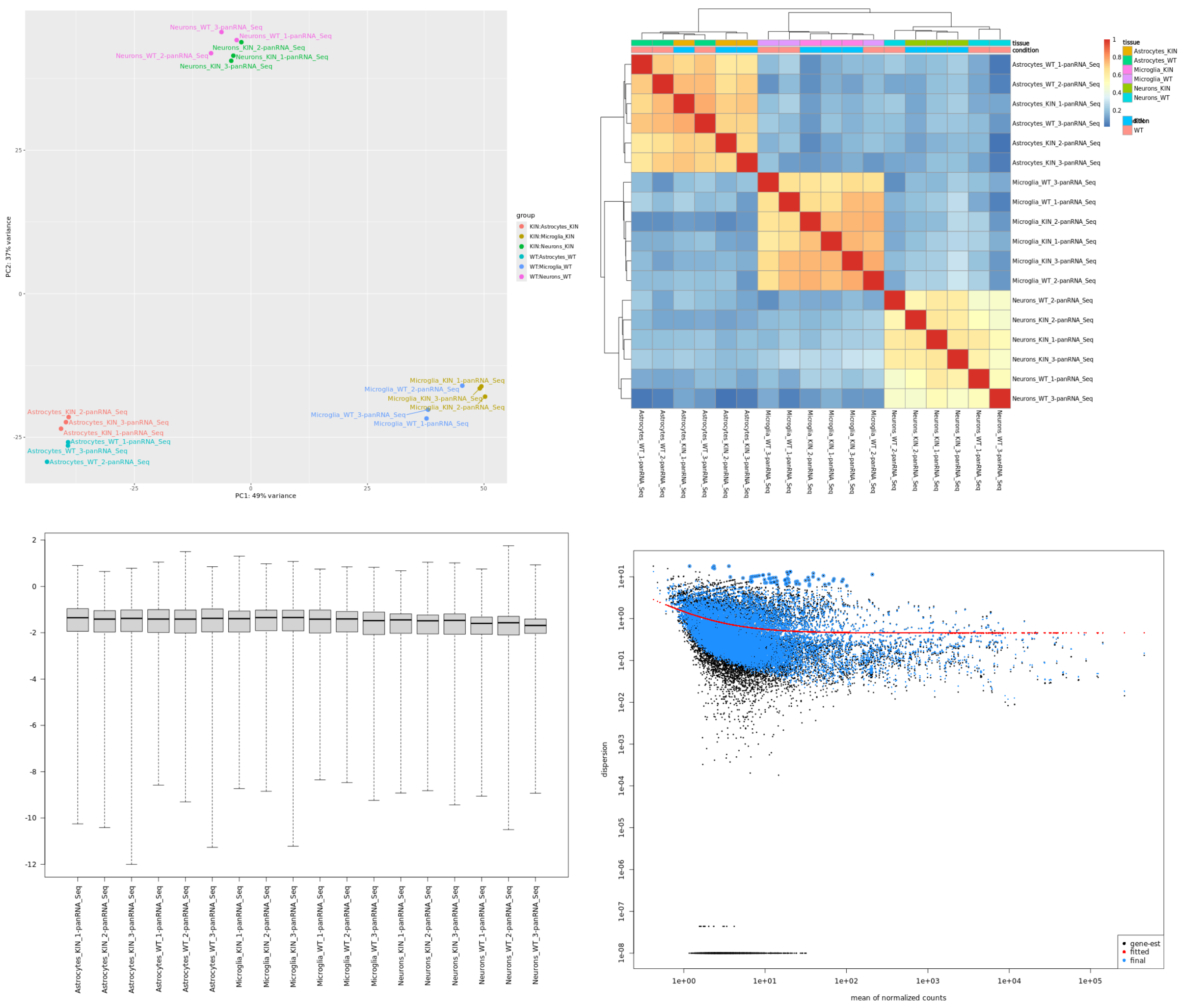

Figure 2. A) Principal Component Analysis (PCA) plot of the top 500 gene with the highest variance across all samples. This plot shows potential clusters based on gene expression. **B)** Heatmap showing the Pearson Correlation Coefficient based on expression of the top 500 gene between all samples. **C)** Boxplot of Cook's distances from DESeq2 analysis, illustrating the distribution of values across samples. A sample with consistently higher Cook's distances may indicate an outlier or a potential issue with model fit. **D)** Dispersion plot from DESeq2 analysis, showing gene-wise, fitted, and final dispersion estimates. Outlier gene-wise estimates are flagged and remain unshrunk, while others are shrunk towards the fitted values for improved model stability.

Expression Quantification

mm39 (Mus musculus)

Genes

We found **11141** genes having an expression of at least 5 in at least 2 samples. The top 10 genes with the highest mean expression in all samples are **Gm12896** (TPM: 49392), **Gm12895** (TPM: 47446), **ENSMUSG00002076138** (TPM: 46289), **mmu-piR-30570** (TPM: 43429), **ENSMUSG00002075676** (TPM: 29613), **mmu-piR-59304** (TPM: 25479), **ENSMUSG00000119892** (TPM: 15162), **BC018473-204** (TPM: 13382), **ENSMUSG00002076655** (TPM: 12572), **ENSMUST00000240065** (TPM: 12192). We identified 2 clusters within the samples based on their expression profiles using hierarchical clustering. These clusters match the variable Tissue with a normalized mutation information (NMI) value of 0.76 (Figure 2).

KIN vs. WT

Table 2. Top 4 differentially expressed gene in KIN compared to WT based on log2 fold change standard error.

Gene	Description	Mean	Log2 Fold Change	Standard Error	P-Value	Adj. P-Value
Bsn	bassoon [Source:MGI Symbol;Acc:MGI:1277955]	18.08	-1.88	0.55	0.00	0.01
Sptbn2	spectrin beta, non-erythrocytic 2 [Source:MGI Symbol;Acc:MGI:1313261]	11.40	-2.20	0.55	0.00	0.01
Dnm1	dynamin 1 [Source:MGI Symbol;Acc:MGI:107384]	12.73	-2.33	0.67	0.00	0.01
Stxbp1	syntaxin binding protein 1 [Source:MGI Symbol;Acc:MGI:107363]	12.64	-1.50	0.68	0.00	0.02

Differential Expression Analysis (DEA)

mm39 (Mus musculus)

Genes

In total, we identified **0 over-expressed** genes and **4 under-expressed** genes in group "KIN" compared to group "WT".

Differentially expressed genes were strictly filtered by mean, log2 fold change and standard error to further increase specificity of down-stream analyses. The most significantly differentially expressed genes were **Bsn, Sptbn2, Dnm1, Stxbp1** (Table 2).

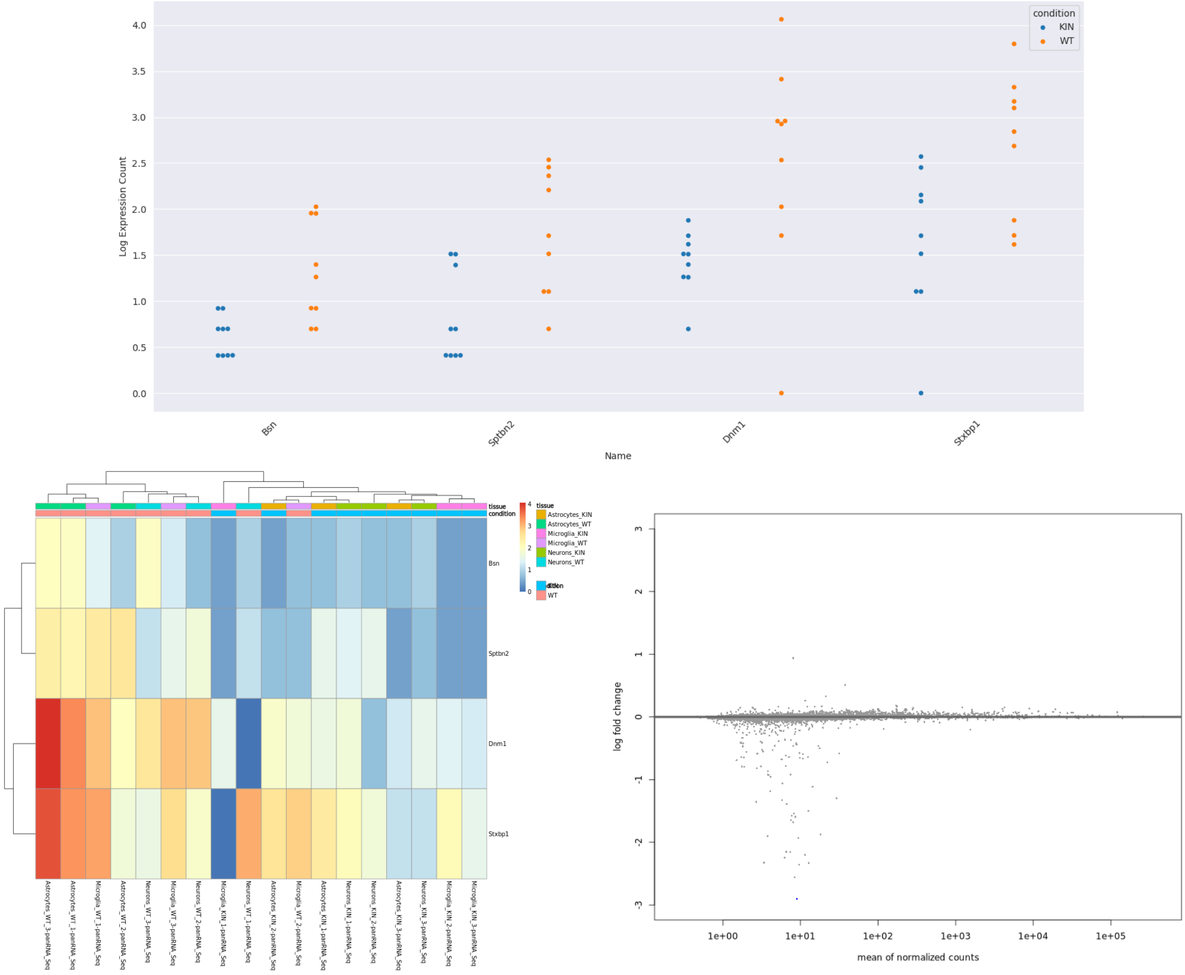

Figure 3. A) Swarm plot showing the log-TPM expression of the genes with the lowest log2 fold change standard error (lfcSE) from the list of significantly differentially expressed genes. **B)** Heatmap showing the log-TPM expression of the genes with the lowest log2 fold change standard error (lfcSE) from the list of significantly differentially expressed genes. **C)** MA plot of differentially expressed genes. The MA plot displays the log2 fold change (y-axis) versus the mean expression (x-axis) for all genes. Significantly differentially expressed genes are highlighted, with points above or below the centerline indicating up-regulated or down-regulated genes. This plot provides insights into the global expression changes and the extent of variability across the feature.dataset.

We created a swarm plot with the standardized and log-transformed expression counts to visually identify false-positive calls (Figure 3A). Using hierarchical clustering on the counts of the differentially expressed genes we **identified clusters** matching the variable Condition with a normalized mutual information (NMI) value of *0.59* (Figure 3B). The global distribution of log2FoldChanges in relation to mean expression is shown in (Figure 3C).

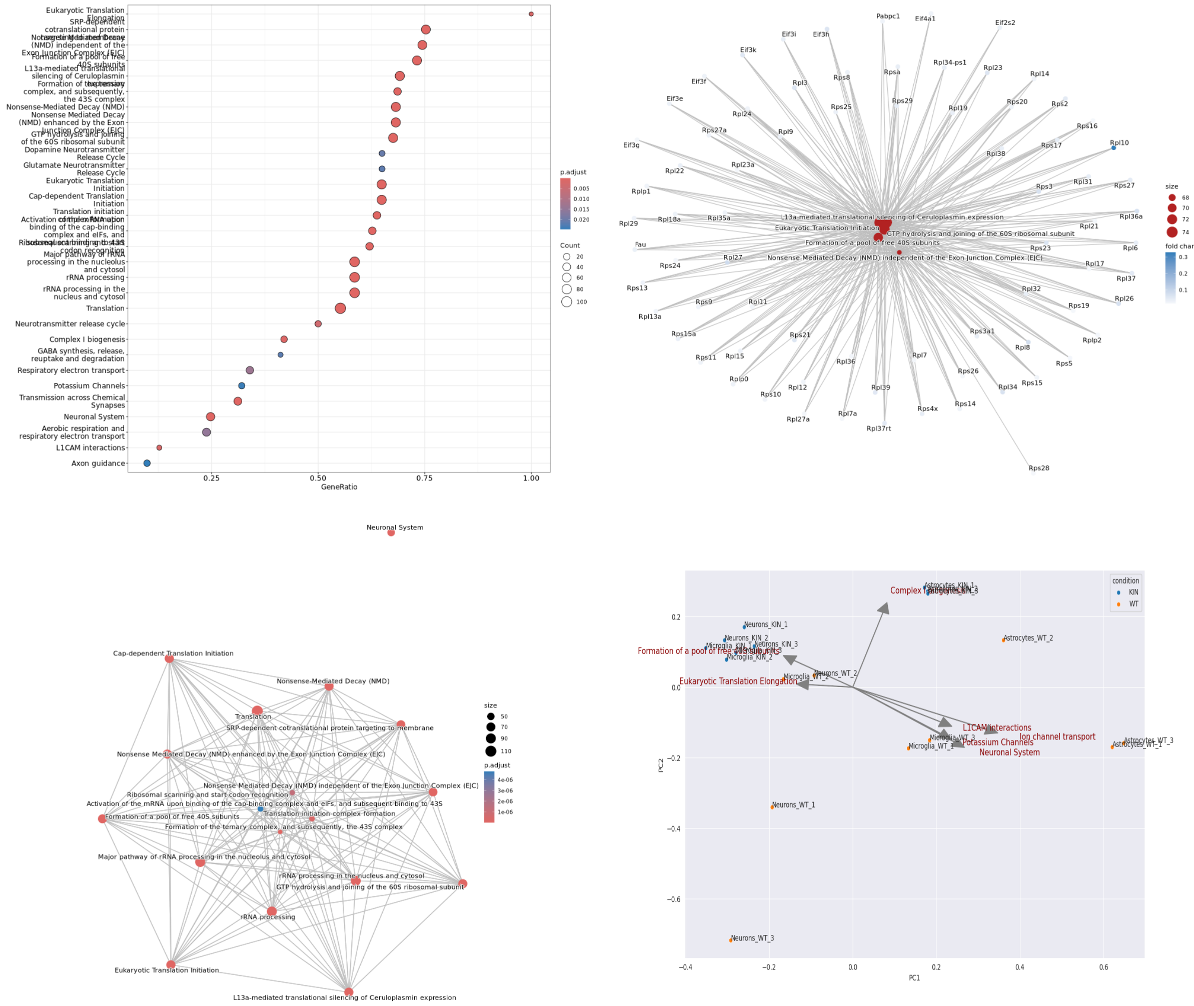

Figure 4. A) Dotplot of enriched pathways. Dots represent enriched pathways, with size indicating gene count and color showing significance (e.g., adjusted p-value). **B)** Cnetplot of gene-pathway relationships. Pathways and their associated genes are connected, with node size and edge thickness indicating enrichment and shared genes. **C)** Enrichment map plot showing overlapping gene sets making it easier to identify functional modules. **D)** PCA biplot showing the average loading of high-variance genes of up to 10 functional modules amongst the enriched terms.

Table 3. Top 15 enriched terms from Reactome in KIN compared to WT. based on gene expression.

ID	Term	NES	Adj. P-Value	Genes
R-MMU-72689	Formation of a pool of free 40S subunits	3.38	0.00	Rpl10, Rpl27, Rpl8, Rpl36a, Rpl26, Rps3, Rpl34, Rps23, Eif3h, Rpl3, Rpl23, Rps21, Eif3k, Rpl39, Rps2, Rpl37rt, Rpl37, Rps27, Rps17, Rps29, Rpl31, Rps4x, Rpl6, Rpl34-ps1, Rpl32, ...
R-MMU-156827	L13a-mediated translational silencing of Ceruloplasmin expression	3.38	0.00	Rpl10, Rpl27, Rpl8, Rpl36a, Rpl26, Rps3, Rpl34, Rps23, Eif3h, Rpl3, Rpl23, Rps21, Eif3k, Rpl39, Rps2, Rpl37rt, Rpl37, Rps27, Rps17, Rps29, Rpl31, Rps4x, Rpl6, Rpl34-ps1, Rpl32, ...
R-MMU-72706	GTP hydrolysis and joining of the 60S ribosomal subunit	3.32	0.00	Rpl10, Rpl27, Rpl8, Rpl36a, Rpl26, Rps3, Rpl34, Rps23, Eif3h, Rpl3, Rpl23, Rps21, Eif3k, Rpl39, Rps2, Rpl37rt, Rpl37, Rps27, Rps17, Rps29, Rpl31, Rps4x, Rpl6, Rpl34-ps1, Rpl32, ...
R-MMU-975956	Nonsense Mediated Decay (NMD) independent of the Exon Junction Complex (EJC)	3.31	0.00	Rpl10, Rpl27, Rpl8, Rpl36a, Rpl26, Rps3, Rpl34, Rps23, Rpl3, Rpl23, Rps21, Rpl39, Rps2, Rpl37rt, Rpl37, Rps27, Rps17, Rps29, Rpl31, Rps4x, Rpl6, Rpl34-ps1, Rpl32, Rps3a1, Rpsa, ...
R-MMU-72613	Eukaryotic Translation Initiation	3.31	0.00	Rpl10, Rpl27, Rpl8, Rpl36a, Rpl26, Rps3, Rpl34, Rps23, Eif3h, Rpl3, Rpl23, Rps21, Eif3k, Rpl39, Rps2, Rpl37rt, Rpl37, Rps27, Rps17, Rps29, Rpl31, Rps4x, Rpl6, Rpl34-ps1, Rpl32, ...
R-MMU-72737	Cap-dependent Translation Initiation	3.31	0.00	Rpl10, Rpl27, Rpl8, Rpl36a, Rpl26, Rps3, Rpl34, Rps23, Eif3h, Rpl3, Rpl23, Rps21, Eif3k, Rpl39, Rps2, Rpl37rt, Rpl37, Rps27, Rps17, Rps29, Rpl31, Rps4x, Rpl6, Rpl34-ps1, Rpl32, ...
R-MMU-1799339	SRP-dependent cotranslational protein targeting to membrane	3.29	0.00	Rpl10, Rpl27, Rpl8, Rpl36a, Rpl26, Rps3, Rpl34, Rps23, Rpl3, Rpl23, Rps21, Rpl39, Rps2, Rpl37rt, Rpl37, Rps27, Rps17, Rps29, Rpl31, Rps4x, Rpl6, Rpl34-ps1, Rpl32, Rps3a1, Rpsa, ...
R-MMU-927802	Nonsense-Mediated Decay (NMD)	3.21	0.00	Rpl10, Rpl27, Rpl8, Rpl36a, Rpl26, Rps3, Rpl34, Rps23, Rpl3, Rpl23, Rps21, Rpl39, Rps2, Rpl37rt, Rpl37, Rps27, Rps17, Rps29, Rpl31, Rps4x, Rpl6, Rpl34-ps1, Rpl32, Rps3a1, Rpsa, ...
R-MMU-975957	Nonsense Mediated Decay (NMD) enhanced by the Exon Junction Complex (EJC)	3.21	0.00	Rpl10, Rpl27, Rpl8, Rpl36a, Rpl26, Rps3, Rpl34, Rps23, Rpl3, Rpl23, Rps21, Rpl39, Rps2, Rpl37rt, Rpl37, Rps27, Rps17, Rps29, Rpl31, Rps4x, Rpl6, Rpl34-ps1, Rpl32, Rps3a1, Rpsa, ...
R-MMU-72766	Translation	3.04	0.00	Rpl10, Rpl27, Rpl8, Rpl36a, Rpl26, Rps3, Rpl34, Rps23, Eif3h, Rpl3, Rpl23, Rps21, Eif3k, Rpl39, Rps2, Eef2, Rpl37rt, Rpl37, Rps27, Eef1b2, Rps17, Rps29, Rpl31, Eef1g, Rps4x, ...
R-MMU-6791226	Major pathway of rRNA processing in the nucleolus and cytosol	3.00	0.00	Rpl10, Rpl27, Rpl8, Rpl36a, Rpl26, Rps3, Rpl34, Rps23, Rpl3, Rpl23, Rps21, Rpl39, Rps2, Rpl37rt, Rpl37, Rps27, Rps17, Rps29, Rpl31, Rps4x, Rpl6, Rpl34-ps1, Rpl32, Rps3a1, Rpsa, ...
R-MMU-72312	rRNA processing	3.00	0.00	Rpl10, Rpl27, Rpl8, Rpl36a, Rpl26, Rps3, Rpl34, Rps23, Rpl3, Rpl23, Rps21, Rpl39, Rps2, Rpl37rt, Rpl37, Rps27, Rps17, Rps29, Rpl31, Rps4x, Rpl6, Rpl34-ps1, Rpl32, Rps3a1, Rpsa, ...
R-MMU-8868773	rRNA processing in the nucleus and cytosol	3.00	0.00	Rpl10, Rpl27, Rpl8, Rpl36a, Rpl26, Rps3, Rpl34, Rps23, Rpl3, Rpl23, Rps21, Rpl39, Rps2, Rpl37rt, Rpl37, Rps27, Rps17, Rps29, Rpl31, Rps4x, Rpl6, Rpl34-ps1, Rpl32, Rps3a1, Rpsa, ...
R-MMU-112316	Neuronal System	-2.08	0.00	Slc38a2, Grin1, Vamp2, Kcnh7, Gls, Rims1, Kcns1, Dnajc5, Cask, Ppfia2, Homer1, Apba1, Nsf, Tspoap1, Nlgn2, Grin2d, Stx1a, Ntrk3, Gabbr2, Gnb1, Kcnj11, Kcnk9, Kcnj3, Syn1, Lrrc7, ...
R-MMU-72695	Formation of the ternary complex, and subsequently, the 43S complex	2.91	0.00	Rps3, Rps23, Eif3h, Rps21, Eif3k, Rps2, Rps27, Rps17, Rps29, Rps4x, Eif3i, Rps3a1, Rpsa, Fau, Eif2s2, Rps25, Rps15a, Rps8, Eif3e, Rps27a, Eif3f, Rps13, Rps20, Rps11, Rps14, ...
...	...	...	...	...

Gene Set Enrichment Analysis (GSEA) for Database "Reactome"

mm39 (Mus musculus)

Genes

We identified **23 up-regulated** and **14 down-regulated** terms from Reactome in group "KIN" compared to group "WT" (Figure 4). The most significantly up-regulated terms were **Formation of a pool of free 40S subunits, L13a-mediated translational silencing of Ceruloplasmin expression, GTP hydrolysis and joining of the 60S ribosomal subunit** . The most significantly down-regulated terms were **Neuronal System, Transmission across Chemical Synapses, L1CAM interactions** . The most significantly terms are listed in Table 3.

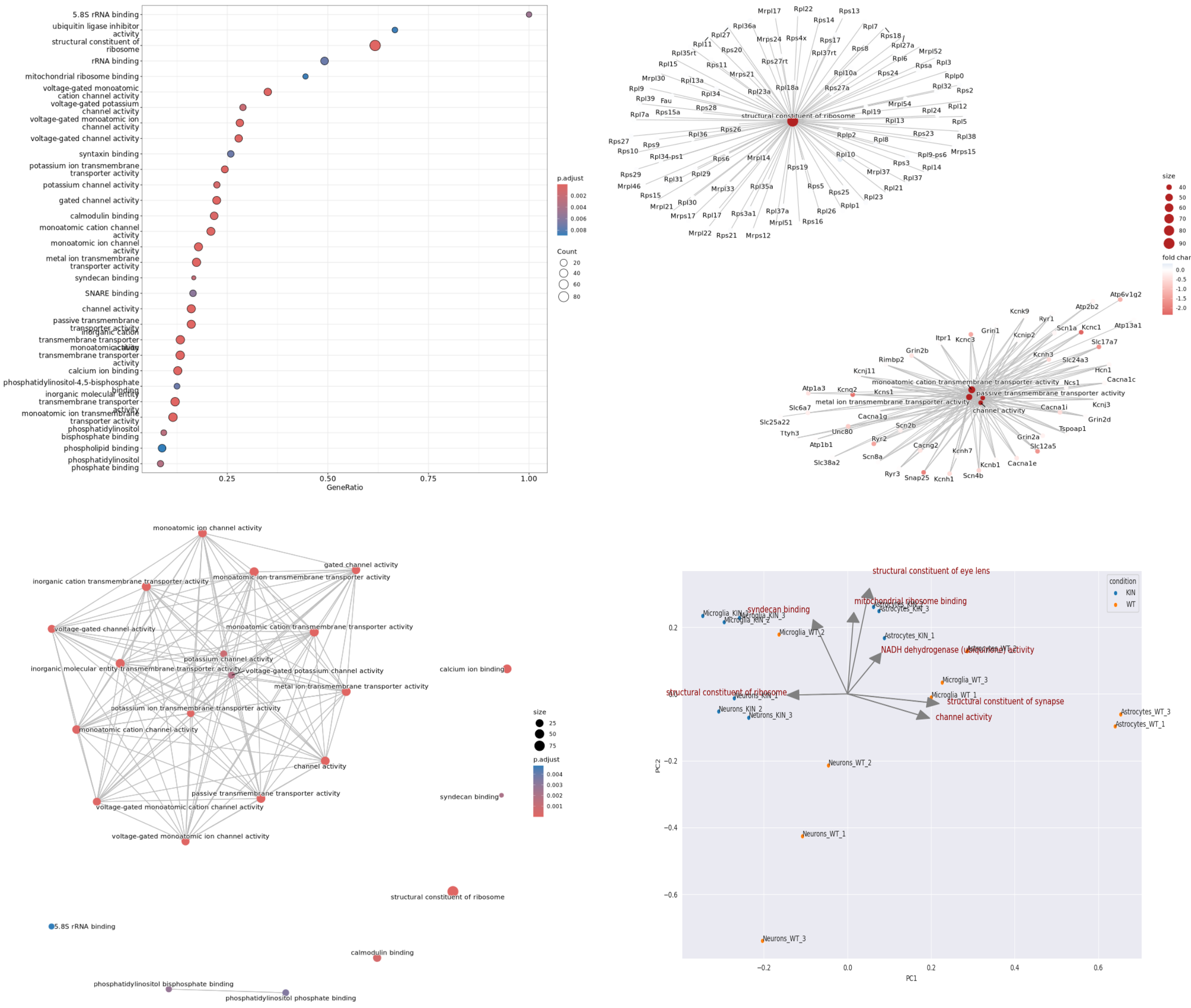

Figure 5. A) Dotplot of enriched pathways. Dots represent enriched pathways, with size indicating gene count and color showing significance (e.g., adjusted p-value). **B)** Cnetplot of gene-pathway relationships. Pathways and their associated genes are connected, with node size and edge thickness indicating enrichment and shared genes. **C)** Enrichment map plot showing overlapping gene sets making it easier to identify functional modules. **D)** PCA biplot showing the average loading of high-variance genes of up to 10 functional modules amongst the enriched terms.

Table 4. Top 15 enriched terms from GO-MF in KIN compared to WT. based on gene expression.

ID	Term	NES	Adj. P-Value	Genes
GO:0003735	structural constituent of ribosome	2.91	0.00	Rpl10, Rpl27, Rpl8, Rpl36a, Rpl26, Rps3, Rpl34, Rps23, Rpl3, Rpl23, Rps21, Rpl39, Rps2, Rpl37rt, Rpl37, Rps27, Rps17, Rps29, Rpl31, Rps4x, Rpl6, Rpl34-ps1, Rpl32, Rps3a1, Rpsa, ...
GO:0015267	channel activity	-1.99	0.00	Ttyh3, Grin1, Kcnh7, Slc24a3, Itpr1, Kcns1, Ryr1, Scn8a, Ryr3, Tspoap1, Grin2d, Kcnip2, Kcnj11, Kcnk9, Kcnj3, Cacna1g, Rimbp2, Kcnb1, Ncs1, Cacna1c, Hcn1, Scn1a, Grin2a, Scn2b, Cacna1e, ...
GO:0022803	passive transmembrane transporter activity	-1.99	0.00	Ttyh3, Grin1, Kcnh7, Slc24a3, Itpr1, Kcns1, Ryr1, Scn8a, Ryr3, Tspoap1, Grin2d, Kcnip2, Kcnj11, Kcnk9, Kcnj3, Cacna1g, Rimbp2, Kcnb1, Ncs1, Cacna1c, Hcn1, Scn1a, Grin2a, Scn2b, Cacna1e, ...
GO:0046873	metal ion transmembrane transporter activity	-1.99	0.00	Slc38a2, Grin1, Kcnh7, Slc24a3, Itpr1, Kcns1, Ryr1, Atp1b1, Scn8a, Ryr3, Tspoap1, Grin2d, Kcnip2, Kcnj11, Kcnk9, Kcnj3, Cacna1g, Rimbp2, Kcnb1, Ncs1, Cacna1c, Hcn1, Scn1a, Atp2b2, Grin2a, ...
GO:0008324	monoatomic cation transmembrane transporter activity	-1.90	0.00	Atp13a1, Slc38a2, Grin1, Slc25a22, Kcnh7, Slc24a3, Itpr1, Kcns1, Ryr1, Atp1b1, Scn8a, Ryr3, Tspoap1, Grin2d, Kcnip2, Kcnj11, Kcnk9, Kcnj3, Cacna1g, Rimbp2, Kcnb1, Ncs1, Cacna1c, Hcn1, Scn1a, ...
GO:0015075	monoatomic ion transmembrane transporter activity	-1.86	0.00	Slc38a2, Ttyh3, Grin1, Slc25a22, Kcnh7, Slc24a3, Itpr1, Kcns1, Ryr1, Atp1b1, Scn8a, Ryr3, Tspoap1, Grin2d, Kcnip2, Kcnj11, Kcnk9, Kcnj3, Cacna1g, Rimbp2, Kcnb1, Ncs1, Cacna1c, Hcn1, Scn1a, ...
GO:0022836	gated channel activity	-2.06	0.00	Ttyh3, Grin1, Kcnh7, Itpr1, Kcns1, Ryr1, Ryr3, Tspoap1, Grin2d, Kcnip2, Kcnj11, Kcnk9, Kcnj3, Cacna1g, Rimbp2, Kcnb1, Ncs1, Cacna1c, Hcn1, Scn1a, Grin2a, Scn2b, Cacna1e, Cacng2, Grin2b, ...
GO:0005216	monoatomic ion channel activity	-2.00	0.00	Ttyh3, Grin1, Kcnh7, Slc24a3, Itpr1, Kcns1, Ryr1, Scn8a, Ryr3, Tspoap1, Grin2d, Kcnip2, Kcnj11, Kcnk9, Kcnj3, Cacna1g, Rimbp2, Kcnb1, Ncs1, Cacna1c, Hcn1, Scn1a, Grin2a, Scn2b, Cacna1e, ...
GO:0022890	inorganic cation transmembrane transporter activity	-1.91	0.00	Slc38a2, Grin1, Slc25a22, Kcnh7, Slc24a3, Itpr1, Kcns1, Ryr1, Atp1b1, Scn8a, Ryr3, Tspoap1, Grin2d, Kcnip2, Kcnj11, Kcnk9, Kcnj3, Cacna1g, Rimbp2, Kcnb1, Ncs1, Cacna1c, Hcn1, Scn1a, Atp2b2, ...
GO:0005509	calcium ion binding	-1.91	0.00	Otof, Ttyh3, Myo5a, Grin1, Cabp1, Mctp1, Cadps, Syt2, Itpr1, Ryr1, Celsr3, Plcg1, Ryr3, Pclo, Kcnip2, Vsnl1, Nell2, Ncs1, Pitpnm3, Dgkg, Spock2, Rasgrp1, Pvalb, Pcp4, Atp2b2, ...
GO:0015318	inorganic molecular entity transmembrane transporter activity	-1.88	0.00	Slc38a2, Ttyh3, Grin1, Slc25a22, Kcnh7, Slc24a3, Itpr1, Kcns1, Ryr1, Atp1b1, Scn8a, Ryr3, Tspoap1, Grin2d, Kcnip2, Kcnj11, Kcnk9, Kcnj3, Cacna1g, Rimbp2, Kcnb1, Ncs1, Cacna1c, Hcn1, Scn1a, ...
GO:0005244	voltage-gated monoatomic ion channel activity	-2.08	0.00	Kcns1, Ryr1, Tspoap1, Grin2d, Kcnip2, Kcnj11, Kcnk9, Kcnj3, Cacna1g, Rimbp2, Kcnb1, Ncs1, Cacna1c, Hcn1, Scn1a, Grin2a, Scn2b, Cacna1e, Cacng2, Grin2b, Cacna1i, Kcnh1, Kcnh3, Kcnc3, Snap25, ...
GO:0022832	voltage-gated channel activity	-2.08	0.00	Kcns1, Ryr1, Tspoap1, Grin2d, Kcnip2, Kcnj11, Kcnk9, Kcnj3, Cacna1g, Rimbp2, Kcnb1, Ncs1, Cacna1c, Hcn1, Scn1a, Grin2a, Scn2b, Cacna1e, Cacng2, Grin2b, Cacna1i, Kcnh1, Kcnh3, Kcnc3, Snap25, ...
GO:0015079	potassium ion transmembrane transporter activity	-2.10	0.00	Kcnh7, Slc24a3, Kcns1, Atp1b1, Kcnip2, Kcnj11, Kcnk9, Kcnj3, Kcnb1, Hcn1, Scn2b, Atp1a3, Kcnh1, Kcnh3, Kcnc3, Slc12a5, Slc17a7, Snap25, Kcnq2, Kcnc1
GO:0022843	voltage-gated monoatomic cation channel activity	-2.10	0.00	Grin1, Kcnh7, Kcns1, Ryr1, Tspoap1, Grin2d, Kcnip2, Kcnj11, Kcnk9, Kcnj3, Cacna1g, Rimbp2, Kcnb1, Ncs1, Cacna1c, Hcn1, Grin2a, Scn2b, Cacna1e, Cacng2, Cacna1i, Kcnh1, Kcnh3, Kcnc3, Snap25, ...
...	...	...	...	...

Gene Set Enrichment Analysis (GSEA) for Database "GO-MF"

mm39 (Mus musculus)

Genes

We identified **12 up-regulated** and **35 down-regulated** terms from GO-MF in group "KIN" compared to group "WT" (Figure 5). The most significantly up-regulated terms were **structural constituent of ribosome, syndecan binding, 5.8S rRNA binding** . The most significantly down-regulated terms were **channel activity, passive transmembrane transporter activity, metal ion transmembrane transporter activity** . The most significantly terms are listed in Table 4.

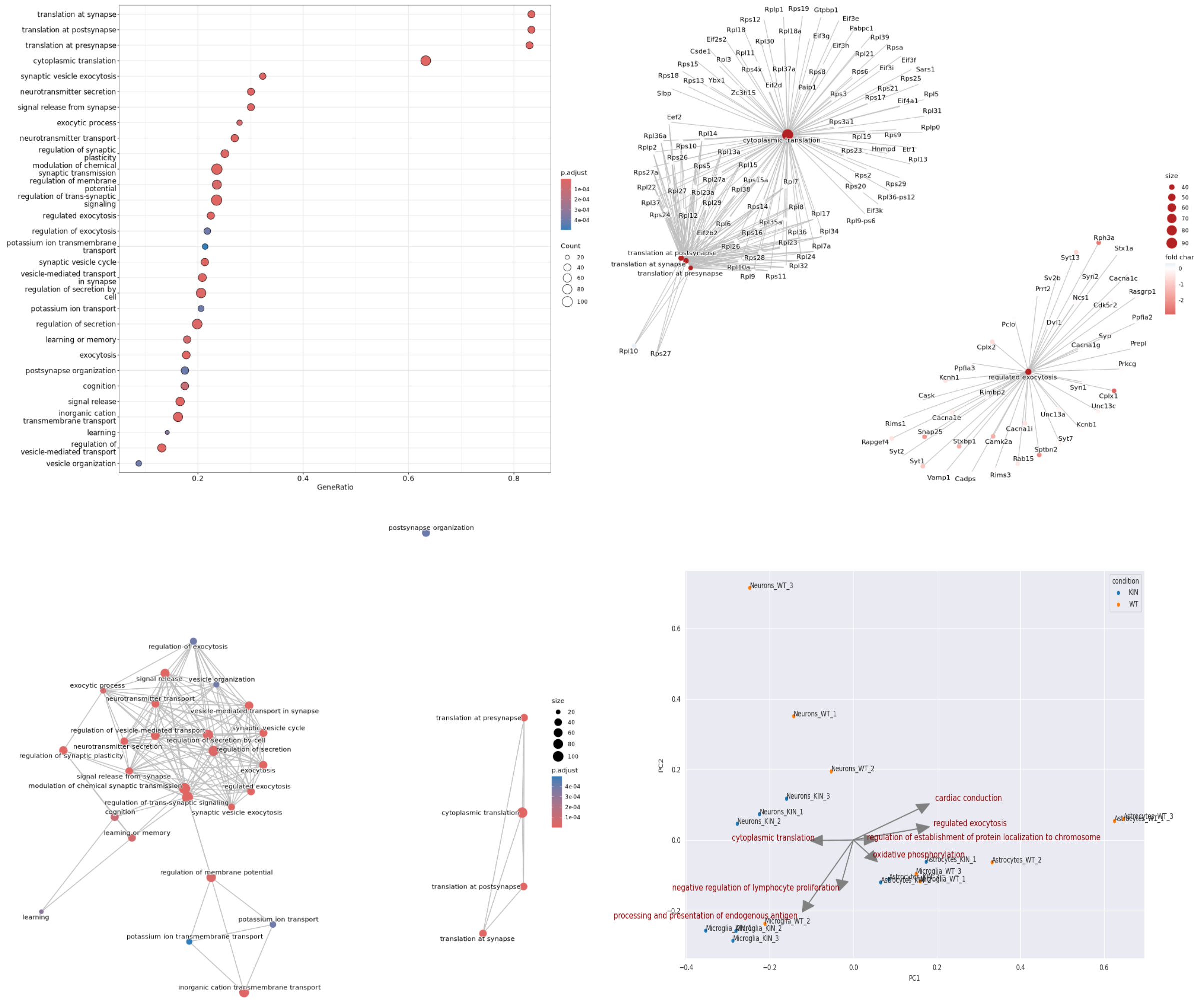

Figure 6. A) Dotplot of enriched pathways. Dots represent enriched pathways, with size indicating gene count and color showing significance (e.g., adjusted p-value). **B)** Cnetplot of gene-pathway relationships. Pathways and their associated genes are connected, with node size and edge thickness indicating enrichment and shared genes. **C)** Enrichment map plot showing overlapping gene sets making it easier to identify functional modules. **D)** PCA biplot showing the average loading of high-variance genes of up to 10 functional modules amongst the enriched terms.

Table 5. Top 15 enriched terms from GO-BP in KIN compared to WT. based on gene expression.

ID	Term	NES	Adj. P-Value	Genes
GO:0002181	cytoplasmic translation	3.02	0.00	Rpl27, Rpl8, Ybx1, Rpl36a, Rpl26, Rps3, Rpl34, Rps23, Eif3h, Rpl3, Rpl23, Rps21, Eif3k, Rpl39, Rps2, Eef2, Rpl37, Rps17, Rps29, Rpl31, Rps4x, Rpl6, Rpl32, Eif3i, Rps3a1, ...
GO:0140241	translation at synapse	3.00	0.00	Rpl10, Rpl27, Rpl8, Rpl36a, Rpl26, Rpl34, Rpl23, Eef2, Rpl37, Rps27, Rpl6, Rpl32, Rpl38, Rpl22, Rpl9, Rps15a, Rps27a, Rpl15, Rpl35a, Rpl10a, Rpl29, Rps11, Rps14, Rpl13a, Rpl24, ...
GO:0140242	translation at postsynapse	3.00	0.00	Rpl10, Rpl27, Rpl8, Rpl36a, Rpl26, Rpl34, Rpl23, Eef2, Rpl37, Rps27, Rpl6, Rpl32, Rpl38, Rpl22, Rpl9, Rps15a, Rps27a, Rpl15, Rpl35a, Rpl10a, Rpl29, Rps11, Rps14, Rpl13a, Rpl24, ...
GO:0140236	translation at presynapse	2.98	0.00	Rpl10, Rpl27, Rpl8, Rpl36a, Rpl26, Rpl34, Rpl23, Rpl37, Rps27, Rpl6, Rpl32, Rpl38, Rpl22, Rpl9, Rps15a, Rps27a, Rpl15, Rpl35a, Rpl10a, Rpl29, Rps11, Rps14, Rpl13a, Rpl24, Rps10, ...
GO:0045055	regulated exocytosis	-2.11	0.00	Cadps, Syt2, Rims1, Cask, Prepl, Ppfia2, Pclo, Dvl1, Prrt2, Stx1a, Syp, Cacna1g, Syn1, Cdk5r2, Rimbp2, Rims3, Kcnb1, Prkcg, Ppfia3, Syn2, Ncs1, Cacna1c, Sv2b, Rasgrp1, Vamp1, ...
GO:0099504	synaptic vesicle cycle	-2.11	0.00	Pten, Cadps, Syt2, Rims1, Dnajc5, Cask, Prepl, Ppfia2, Pclo, Dvl1, Nlgn2, Prrt2, Stx1a, Syp, Syn1, Rimbp2, Pacsin1, Rims3, Prkcg, Ppfia3, Syn2, Ncs1, Sv2b, Vamp1, Syt7, ...
GO:0099003	vesicle-mediated transport in synapse	-2.06	0.00	Otof, Vamp2, Ap3d1, Necap1, Pten, Cadps, Syt2, Rims1, Rabep1, Dnajc5, Cask, Prepl, Ppfia2, Aak1, Pclo, Dvl1, Nlgn2, Prrt2, Stx1a, Usp46, Syp, Syn1, Rimbp2, Pacsin1, Rims3, ...
GO:0006887	exocytosis	-2.00	0.00	Otof, Myo5a, Vamp2, Cadps, Syt2, Rims1, Cask, Prepl, Ppfia2, Pclo, Dvl1, Smpd3, Nsf, Prrt2, Stx1a, Vsnl1, Syp, Cacna1g, Syn1, Cdk5r2, Rimbp2, Rims3, Kcnb1, Prkcg, Ppfia3, ...
GO:0099177	regulation of trans-synaptic signaling	-1.96	0.00	Afdn, Ina, Shank2, Cacna1a, Grip2, Shisa9, Ppp3r1, Grm7, Atp2a2, Slc8a2, Gabbr1, Kat2a, Hap1, Rasgrf1, Hras, Mapk1, Eif2ak4, Sipa1l1, Gria2, Arhgap44, Cntnap2, Akap7, Shisa7, Vgf, Slc38a2, ...
GO:0050804	modulation of chemical synaptic transmission	-1.96	0.00	Afdn, Ina, Shank2, Cacna1a, Grip2, Shisa9, Ppp3r1, Grm7, Atp2a2, Slc8a2, Gabbr1, Kat2a, Hap1, Rasgrf1, Hras, Mapk1, Eif2ak4, Sipa1l1, Gria2, Arhgap44, Cntnap2, Akap7, Shisa7, Vgf, Slc38a2, ...
GO:0006836	neurotransmitter transport	-2.09	0.00	Slc38a2, Otof, Vamp2, Mctp1, Cadps, Syt2, Rims1, Cask, Prepl, Ppfia2, Ppp1r9a, Pclo, Dvl1, Tspoap1, Prrt2, Stx1a, Syp, Syn1, Rimbp2, Rims3, Prkcg, Ppfia3, Syn2, Ncs1, Sv2b, ...
GO:0023061	signal release	-1.88	0.00	Vgf, Slc38a2, Otof, Myo5a, Vamp2, Cxcl12, Mctp1, Slc25a22, Kif5b, Cadps, Syt2, Rims1, Itpr1, Cask, Prepl, Ppfia2, Ppp1r9a, Pclo, Dvl1, Smpd3, Tspoap1, Nlgn2, Prrt2, Stx1a, Oga, ...
GO:0060627	regulation of vesicle-mediated transport	-1.84	0.00	Myo5a, Vamp2, Mctp1, Kif5b, Pten, Cadps, Ank3, Syt2, Rims1, Rabep1, Dnajc5, Cask, Prepl, Ppfia2, Aak1, Pclo, Src, Dvl1, Smpd3, Nsf, Tub, Prrt2, Stx1a, Vsnl1, Usp46, ...
GO:0007269	neurotransmitter secretion	-2.10	0.00	Slc38a2, Otof, Vamp2, Mctp1, Cadps, Syt2, Rims1, Cask, Prepl, Ppfia2, Ppp1r9a, Pclo, Dvl1, Tspoap1, Prrt2, Stx1a, Syp, Syn1, Rimbp2, Rims3, Prkcg, Ppfia3, Syn2, Ncs1, Sv2b, ...
GO:0099643	signal release from synapse	-2.10	0.00	Slc38a2, Otof, Vamp2, Mctp1, Cadps, Syt2, Rims1, Cask, Prepl, Ppfia2, Ppp1r9a, Pclo, Dvl1, Tspoap1, Prrt2, Stx1a, Syp, Syn1, Rimbp2, Rims3, Prkcg, Ppfia3, Syn2, Ncs1, Sv2b, ...
...	...	...	...	...

Gene Set Enrichment Analysis (GSEA) for Database "GO-BP"

mm39 (Mus musculus)

Genes

We identified **27 up-regulated** and **128 down-regulated** terms from GO-BP in group "KIN" compared to group "WT" (Figure 6). The most significantly up-regulated terms were **cytoplasmic translation, translation at synapse, translation at postsynapse** . The most significantly down-regulated terms were **regulated exocytosis, synaptic vesicle cycle, vesicle-mediated transport in synapse** . The most significantly terms are listed in Table 5.

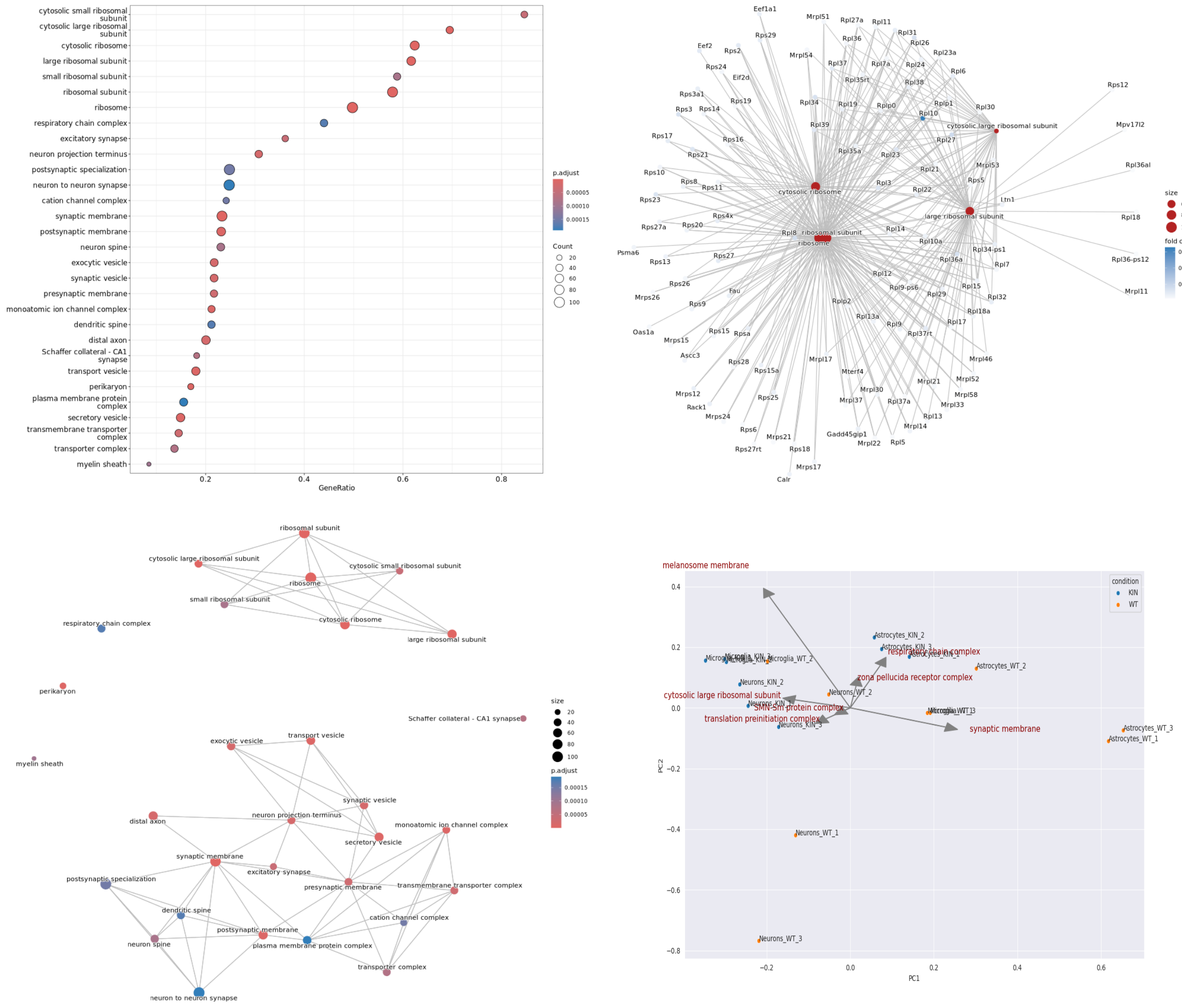

Figure 7. A) Dotplot of enriched pathways. Dots represent enriched pathways, with size indicating gene count and color showing significance (e.g., adjusted p-value). **B)** Cnetplot of gene-pathway relationships. Pathways and their associated genes are connected, with node size and edge thickness indicating enrichment and shared genes. **C)** Enrichment map plot showing overlapping gene sets making it easier to identify functional modules. **D)** PCA biplot showing the average loading of high-variance genes of up to 10 functional modules amongst the enriched terms.

Table 6. Top 15 enriched terms from GO-CC in KIN compared to WT. based on gene expression.

ID	Term	NES	Adj. P-Value	Genes
GO:0022625	cytosolic large ribosomal subunit	2.97	0.00	Rpl10, Rpl27, Rpl8, Rpl36a, Rpl26, Rpl34, Rpl3, Rpl23, Rpl39, Rpl37rt, Rpl37, Rpl31, Rpl6, Rpl34-ps1, Rpl32, Rpl38, Rpl22, Rpl19, Rpl9, Rpl15, Rpl18a, Rpl35a, Rpl10a, Rplp0, Rpl29, ...
GO:0022626	cytosolic ribosome	2.97	0.00	Rpl10, Rpl27, Rpl8, Rpl36a, Rpl26, Rps3, Rpl34, Rps23, Rpl3, Rpl23, Rps21, Rpl39, Rps2, Rpl37rt, Rpl37, Rps27, Rps17, Rps29, Rpl31, Rps4x, Rpl6, Rpl34-ps1, Rpl32, Rps3a1, Rpsa, ...
GO:0044391	ribosomal subunit	2.92	0.00	Rpl10, Rpl27, Rpl8, Rpl36a, Rpl26, Rps3, Rpl34, Rps23, Rpl3, Rpl23, Rps21, Rpl39, Rps2, Rpl37rt, Rpl37, Rps27, Rps17, Rps29, Rpl31, Rps4x, Rpl6, Rpl34-ps1, Rpl32, Rps3a1, Rpsa, ...
GO:0015934	large ribosomal subunit	2.75	0.00	Rpl10, Rpl27, Rpl8, Rpl36a, Rpl26, Rpl34, Rpl3, Rpl23, Rpl39, Rpl37rt, Rpl37, Rpl31, Rpl6, Rpl34-ps1, Rpl32, Rpl38, Rpl22, Rpl19, Gadd45gip1, Rpl9, Mrpl17, Rpl15, Rpl18a, Rpl35a, Rpl10a, ...
GO:0005840	ribosome	2.73	0.00	Rpl10, Rpl27, Rpl8, Rpl36a, Rpl26, Rps3, Rpl34, Rps23, Rpl3, Rpl23, Rps21, Rpl39, Rps2, Eef2, Rpl37rt, Rpl37, Rps27, Rps17, Rps29, Rpl31, Rps4x, Rpl6, Rpl34-ps1, Rpl32, Rps3a1, ...
GO:0097060	synaptic membrane	-1.97	0.00	Rab3gap2, Dlg3, Cacna2d1, Adgrl2, Sez6l, Afdn, Shank2, Kcna1, Cacna1a, Grip2, Shisa9, Grm7, Slc8a2, Kcnj9, Gabbr1, Snap91, Ank2, Kcna2, Dagla, Synj1, Gria2, Cntnap2, Gabra4, Shisa7, Nsg2, ...
GO:0043204	perikaryon	-2.09	0.00	Nell2, Rnf112, Gap43, Kcnb1, Pcsk2, Cacna1c, Rgs8, Ncdn, Rgs7bp, Ckmt1, Map1b, Eno2, Pde10a, Cacna1e, Rbfox3, Ddn, Kcnh1, Nefm, Camk2b, Cplx2, Hpca, Kif5a, Kcnc3, Slc12a5, Nefh, ...
GO:0099503	secretory vesicle	-1.88	0.00	Gabbr1, Snap91, Calm2, Ptprn2, Hap1, Dennd4c, Dmxl2, Slc32a1, Gria2, Slc6a17, Znrf1, Slc2a8, Akap7, Kif3c, Otof, Myo5a, Grin1, Vamp2, Cabp1, Mctp1, Apc, Cadps, Kif1a, Syt2, Itpr1, ...
GO:0034702	monoatomic ion channel complex	-2.04	0.00	Ttyh3, Grin1, Vamp2, Kcnh7, Kcns1, Ryr1, Scn8a, Ryr3, Akap6, Grin2d, Kcnip2, Abhd6, Stx1a, Kcnj11, Kcnj3, Dpp6, Cacna1g, Kcnb1, Cacna1c, Hcn1, Scn1a, Dlg4, Grin2a, Pde4d, Scn2b, ...
GO:0030133	transport vesicle	-1.93	0.00	Gabbr1, Snap91, Calm2, Ptprn2, Hap1, Dmxl2, Slc32a1, Gria2, Sec24c, Slc6a17, Znrf1, Slc2a8, Akap7, Gnas, Kif3c, Vgf, Otof, Myo5a, Grin1, Vamp2, Mctp1, Apc, Kif1a, Syt2, Itpr1, ...
GO:0045211	postsynaptic membrane	-1.96	0.00	Dlg3, Cacna2d1, Adgrl2, Sez6l, Afdn, Shank2, Kcna1, Cacna1a, Grip2, Shisa9, Grm7, Slc8a2, Gabbr1, Snap91, Ank2, Kcna2, Dagla, Gria2, Gabra4, Shisa7, Nsg2, Grin1, Neto1, Pten, Ank3, ...
GO:0070382	exocytic vesicle	-1.99	0.00	Gabbr1, Snap91, Calm2, Ptprn2, Hap1, Dmxl2, Slc32a1, Gria2, Slc6a17, Znrf1, Slc2a8, Akap7, Kif3c, Otof, Myo5a, Grin1, Vamp2, Mctp1, Apc, Kif1a, Syt2, Dnajc5, Ppfia2, Prrt2, Stx1a, ...
GO:0008021	synaptic vesicle	-1.99	0.00	Gabbr1, Snap91, Calm2, Ptprn2, Hap1, Dmxl2, Slc32a1, Gria2, Slc6a17, Znrf1, Slc2a8, Kif3c, Otof, Myo5a, Grin1, Vamp2, Mctp1, Apc, Kif1a, Syt2, Dnajc5, Ppfia2, Prrt2, Stx1a, Kcnk9, ...
GO:0150034	distal axon	-1.92	0.00	Tsc2, Grm7, Slc8a2, Snap91, Calm2, Kcna2, Ptprn2, Hap1, Rasgrf1, Slc32a1, Setx, Synj1, Gria2, Kif3c, Klc1, Mapk8ip3, Grin1, Cables1, Ppp2ca, Ap3d1, Apc, Kif5b, Lmtk2, Ppp1r9a, Aak1, ...
GO:0044306	neuron projection terminus	-2.07	0.00	Grm7, Slc8a2, Snap91, Calm2, Kcna2, Ptprn2, Hap1, Slc32a1, Synj1, Gria2, Ptpn9, Grin1, Vamp2, Ppp2ca, Ap3d1, Apc, Aak1, Pclo, Tspoap1, Prrt2, Syp, Rimbp2, Pacsin1, Prkcg, Ncs1, ...
...	...	...	...	...

Gene Set Enrichment Analysis (GSEA) for Database "GO-CC"

mm39 (Mus musculus)

Genes

We identified **22 up-regulated** and **79 down-regulated** terms from GO-CC in group "KIN" compared to group "WT" (Figure 7). The most significantly up-regulated terms were **cytosolic large ribosomal subunit, cytosolic ribosome, ribosomal subunit** . The most significantly down-regulated terms were **synaptic membrane, perikaryon, secretory vesicle** . The most significantly terms are listed in Table 6.

Astrocytes_KIN vs. Astrocytes_WT

Table 7. Top 20 differentially expressed gene in Astrocytes_KIN compared to Astrocytes_WT based on log2 fold change standard error.

Gene	Description	Mean	Log2 Fold Change	Standard Error	P-Value	Adj. P-Value
Gm24507-201	predicted gene, 24507 [Source:MGI Symbol;Acc:MGI:5454284]	284.38	1.02	0.22	0.00	0.00
Kcnq1ot1-201	KCNQ1 overlapping transcript 1 [Source:MGI Symbol;Acc:MGI:1926855]	346.30	-1.13	0.24	0.00	0.00
Slc1a2	solute carrier family 1 (glial high affinity glutamate transporter), member 2 [Source:MGI Symbol;Acc:MGI:101931]	423.36	-1.23	0.25	0.00	0.00
Fgfr3	fibroblast growth factor receptor 3 [Source:MGI Symbol;Acc:MGI:95524]	154.15	-1.01	0.25	0.00	0.00
Glul	glutamate-ammonia ligase (glutamine synthetase) [Source:MGI Symbol;Acc:MGI:95739]	199.39	-1.08	0.25	0.00	0.00
Slc6a11	solute carrier family 6 (neurotransmitter transporter, GABA), member 11 [Source:MGI Symbol;Acc:MGI:95630]	251.50	-1.40	0.26	0.00	0.00
Lgi3	leucine-rich repeat LGI family, member 3 [Source:MGI Symbol;Acc:MGI:2182619]	153.36	1.22	0.26	0.00	0.00
Slc4a4	solute carrier family 4 (anion exchanger), member 4 [Source:MGI Symbol;Acc:MGI:1927555]	122.82	-1.17	0.27	0.00	0.00
Mfge8	milk fat globule-EGF factor 8 protein [Source:MGI Symbol;Acc:MGI:102768]	170.53	-1.24	0.27	0.00	0.00
Gja1	gap junction protein, alpha 1 [Source:MGI Symbol;Acc:MGI:95713]	178.45	-1.30	0.28	0.00	0.00
ENS-MUST00000243755	nan	178.45	-1.12	0.28	0.00	0.00
Gm47578-201	predicted gene, 47578 [Source:MGI Symbol;Acc:MGI:6096613]	542.14	-1.36	0.28	0.00	0.00
Plpp3	phospholipid phosphatase 3 [Source:MGI Symbol;Acc:MGI:1915166]	202.49	-1.34	0.28	0.00	0.00
AC139131.1-201	novel transcript	194.19	-1.36	0.29	0.00	0.00
Htra1	HtrA serine peptidase 1 [Source:MGI Symbol;Acc:MGI:1929076]	161.30	-1.43	0.30	0.00	0.00
Gm31392-201	predicted gene, 31392 [Source:MGI Symbol;Acc:MGI:5590551]	76.91	-1.04	0.32	0.00	0.00
Gm45792-201	predicted gene 45792 [Source:MGI Symbol;Acc:MGI:5804907]	81.58	-1.14	0.32	0.00	0.00
Agt	angiotensinogen (serpin peptidase inhibitor, clade A, member 8) [Source:MGI Symbol;Acc:MGI:87963]	80.68	-2.15	0.32	0.00	0.00
Gm48765-201	predicted gene, 48765 [Source:MGI Symbol;Acc:MGI:6098451]	141.34	-1.30	0.32	0.00	0.00
Gm49534-201	predicted gene, 49534 [Source:MGI Symbol;Acc:MGI:6155235]	175.22	-1.35	0.33	0.00	0.00
...	...	...	...	...	...	...

Differential Expression Analysis (DEA)

mm39 (Mus musculus)

Genes

In total, we identified **7 over-expressed** genes and **112 under-expressed** genes in group "Astrocytes_KIN" compared to group "Astrocytes_WT". Differentially expressed genes were strictly filtered by mean, log2 fold change and standard error to further increase specificity of down-stream analyses. The most significantly differentially expressed genes were **Gm24507-201, Kcnq1ot1-201, Slc1a2, Fgfr3, Glul, Slc6a11, Lgi3, Slc4a4, Mfge8, Gja1** (Table 7).

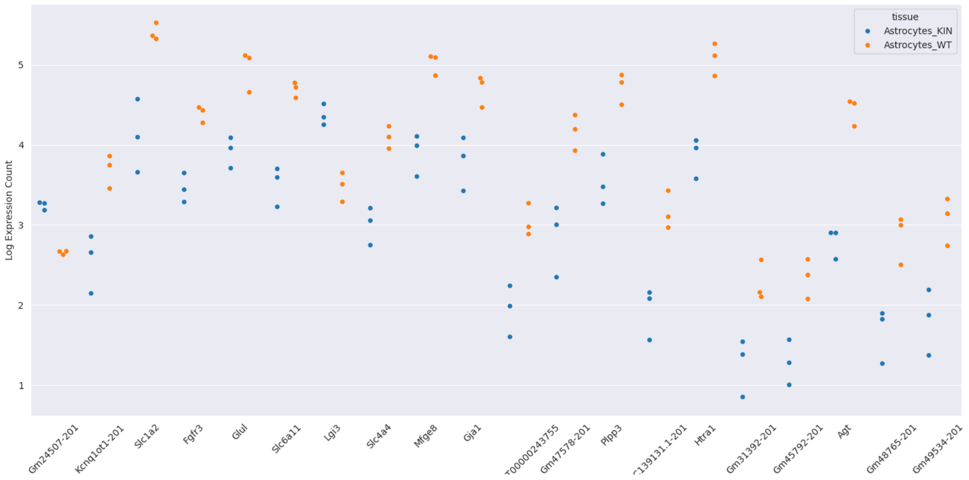

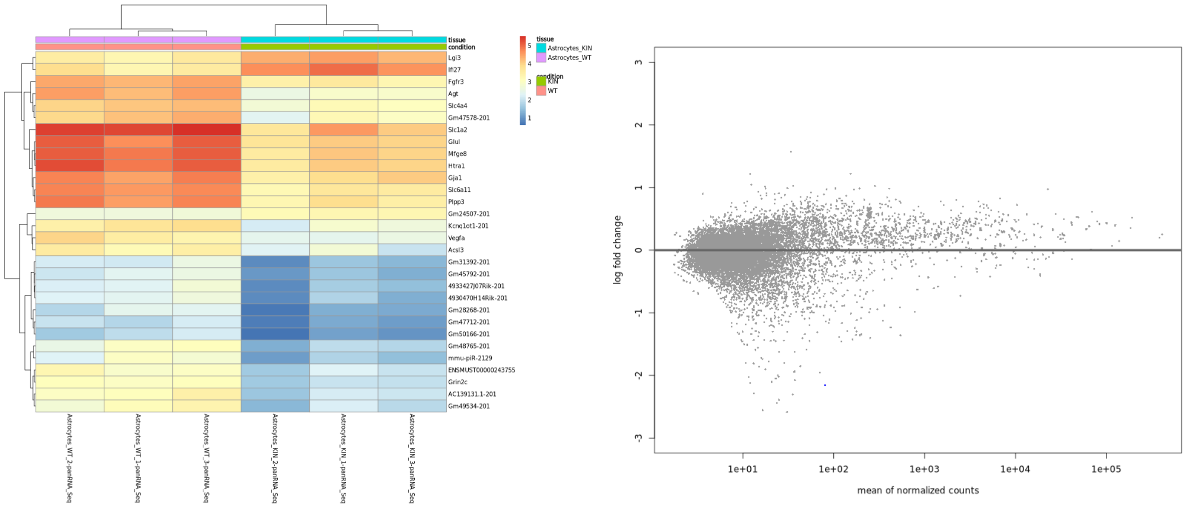

Figure 8. A) Swarm plot showing the log-TPM expression of the genes with the lowest log2 fold change standard error (lfcSE) from the list of significantly differentially expressed genes. **B)** Heatmap showing the log-TPM expression of the genes with the lowest log2 fold change standard error (lfcSE) from the list of significantly differentially expressed genes. **C)** MA plot of differentially expressed genes. The MA plot displays the log2 fold change (y-axis) versus the mean expression (x-axis) for all genes. Significantly differentially expressed genes are highlighted, with points above or below the centerline indicating up-regulated or down-regulated genes. This plot provides insights into the global expression changes and the extent of variability across the feature.dataset.

We created a swarm plot with the standardized and log-transformed expression counts to visually identify false-positive calls (Figure 8A). Using hierarchical clustering on the counts of the differentially expressed genes we **identified clusters** matching the variable Condition with a normalized mutual information (NMI) value of *1.0* (Figure 8B). and we **identified clusters** matching the variable Tissue with a normalized mutual information (NMI) value of *1.0* (Figure 8B). The global distribution of log2FoldChanges in relation to mean expression is shown in (Figure 8C).

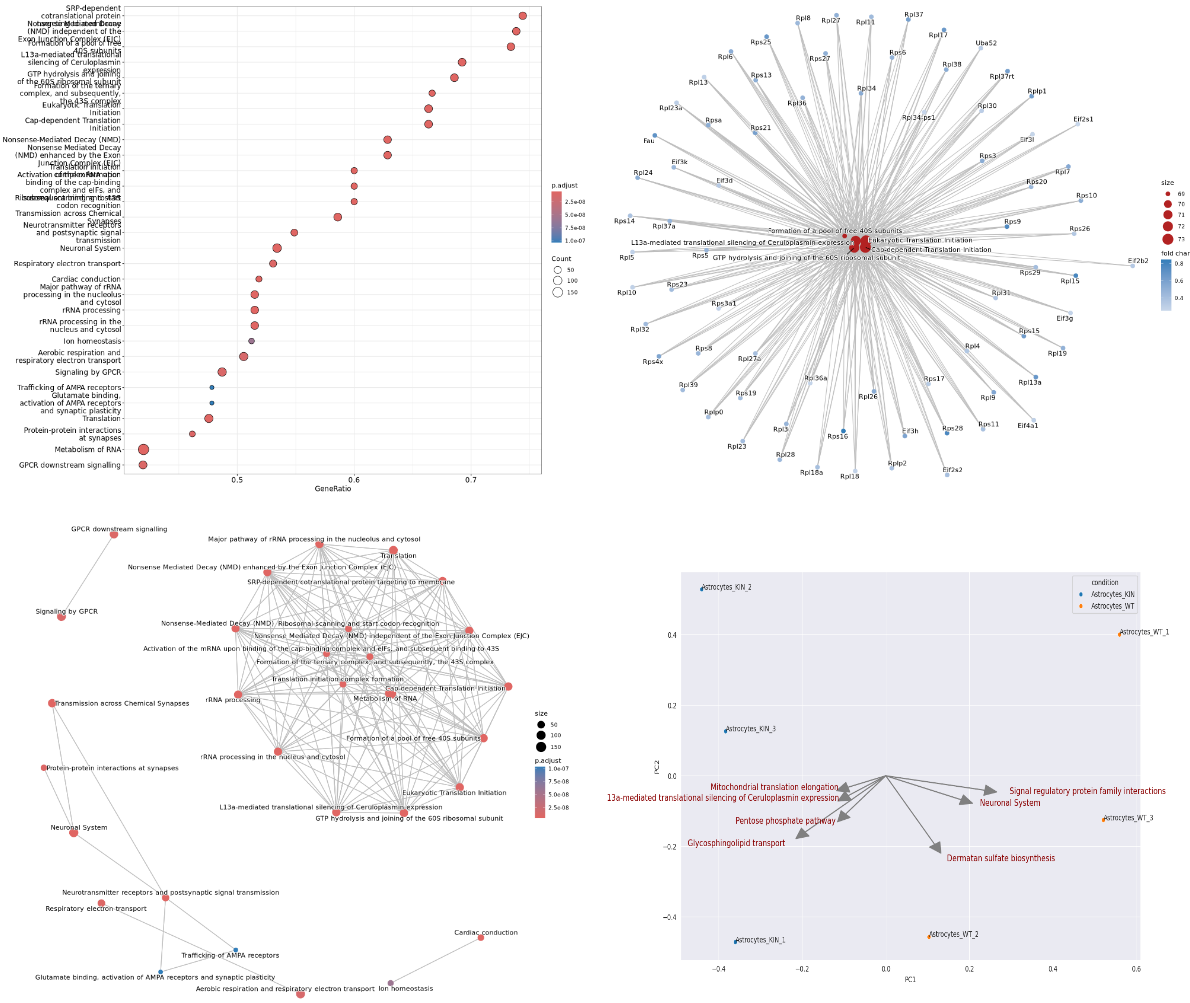

Figure 9. A) Dotplot of enriched pathways. Dots represent enriched pathways, with size indicating gene count and color showing significance (e.g., adjusted p-value). **B)** Cnetplot of gene-pathway relationships. Pathways and their associated genes are connected, with node size and edge thickness indicating enrichment and shared genes. **C)** Enrichment map plot showing overlapping gene sets making it easier to identify functional modules. **D)** PCA biplot showing the average loading of high-variance genes of up to 10 functional modules amongst the enriched terms.

Table 8. Top 15 enriched terms from Reactome in Astrocytes_KIN compared to Astrocytes_WT. based on gene expression.

ID	Term	NES	Adj. P-Value	Genes
R-MMU-156827	L13a-mediated translational silencing of Ceruloplasmin expression	3.50	0.00	Rps16, Rpl15, Rps28, Rps9, Fau, Rpl13a, Rps25, Rpl17, Rplp1, Rps15, Rpl37rt, Rpl37, Rps21, Eif3h, Rps4x, Rpl9, Rpl7, Rpl34, Rps3, Rps10, Rpl38, Rpl26, Rpsa, Rpl24, Rpl6, ...
R-MMU-72689	Formation of a pool of free 40S subunits	3.49	0.00	Rps16, Rpl15, Rps28, Rps9, Fau, Rpl13a, Rps25, Rpl17, Rplp1, Rps15, Rpl37rt, Rpl37, Rps21, Eif3h, Rps4x, Rpl9, Rpl7, Rpl34, Rps3, Rps10, Rpl38, Rpl26, Rpsa, Rpl24, Rpl6, ...
R-MMU-72706	GTP hydrolysis and joining of the 60S ribosomal subunit	3.49	0.00	Rps16, Rpl15, Rps28, Rps9, Fau, Rpl13a, Rps25, Rpl17, Rplp1, Rps15, Rpl37rt, Rpl37, Rps21, Eif3h, Rps4x, Rpl9, Rpl7, Rpl34, Rps3, Rps10, Rpl38, Rpl26, Rpsa, Rpl24, Rpl6, ...
R-MMU-72613	Eukaryotic Translation Initiation	3.48	0.00	Rps16, Rpl15, Rps28, Rps9, Fau, Rpl13a, Rps25, Rpl17, Rplp1, Rps15, Rpl37rt, Rpl37, Rps21, Eif3h, Rps4x, Rpl9, Rpl7, Rpl34, Rps3, Rps10, Rpl38, Rpl26, Rpsa, Rpl24, Rpl6, ...
R-MMU-72737	Cap-dependent Translation Initiation	3.48	0.00	Rps16, Rpl15, Rps28, Rps9, Fau, Rpl13a, Rps25, Rpl17, Rplp1, Rps15, Rpl37rt, Rpl37, Rps21, Eif3h, Rps4x, Rpl9, Rpl7, Rpl34, Rps3, Rps10, Rpl38, Rpl26, Rpsa, Rpl24, Rpl6, ...
R-MMU-975956	Nonsense Mediated Decay (NMD) independent of the Exon Junction Complex (EJC)	3.48	0.00	Rps16, Rpl15, Rps28, Rps9, Fau, Rpl13a, Rps25, Rpl17, Rplp1, Rps15, Rpl37rt, Rpl37, Rps21, Rps4x, Rpl9, Rpl7, Rpl34, Rps3, Rps10, Rpl38, Rpl26, Rpsa, Rpl24, Rpl6, Rps13, ...
R-MMU-1799339	SRP-dependent cotranslational protein targeting to membrane	3.47	0.00	Rps16, Rpl15, Rps28, Rps9, Fau, Rpl13a, Rps25, Rpl17, Rplp1, Rps15, Rpl37rt, Rpl37, Rps21, Rps4x, Rpl9, Rpl7, Rpl34, Rps3, Rps10, Rpl38, Rpl26, Rpsa, Rpl24, Rpl6, Rps13, ...
R-MMU-927802	Nonsense-Mediated Decay (NMD)	3.39	0.00	Rps16, Rpl15, Rps28, Rps9, Fau, Rpl13a, Rps25, Rpl17, Rplp1, Rps15, Rpl37rt, Rpl37, Rps21, Rps4x, Rpl9, Rpl7, Rpl34, Rps3, Rps10, Rpl38, Rpl26, Rpsa, Rpl24, Rpl6, Rps13, ...
R-MMU-975957	Nonsense Mediated Decay (NMD) enhanced by the Exon Junction Complex (EJC)	3.39	0.00	Rps16, Rpl15, Rps28, Rps9, Fau, Rpl13a, Rps25, Rpl17, Rplp1, Rps15, Rpl37rt, Rpl37, Rps21, Rps4x, Rpl9, Rpl7, Rpl34, Rps3, Rps10, Rpl38, Rpl26, Rpsa, Rpl24, Rpl6, Rps13, ...
R-MMU-72766	Translation	3.22	0.00	Rps16, Rpl15, Mrpl52, Rps28, Rps9, Fau, Rpl13a, Rps25, Rpl17, Rplp1, Rps15, Rpl37rt, Rpl37, Rps21, Eif3h, Rps4x, Rpl9, Rpl7, Rpl34, Rps3, Gadd45gip1, Rps10, Rpl38, Rpl26, Rpsa, ...
R-MMU-6791226	Major pathway of rRNA processing in the nucleolus and cytosol	3.13	0.00	Rps16, Rpl15, Rps28, Rps9, Fau, Rpl13a, Rps25, Rpl17, Rplp1, Rps15, Rpl37rt, Rpl37, Rps21, Rps4x, Rpl9, Rpl7, Rpl34, Rps3, Rps10, Rpl38, Rpl26, Rpsa, Rpl24, Rpl6, Rps13, ...
R-MMU-72312	rRNA processing	3.13	0.00	Rps16, Rpl15, Rps28, Rps9, Fau, Rpl13a, Rps25, Rpl17, Rplp1, Rps15, Rpl37rt, Rpl37, Rps21, Rps4x, Rpl9, Rpl7, Rpl34, Rps3, Rps10, Rpl38, Rpl26, Rpsa, Rpl24, Rpl6, Rps13, ...
R-MMU-8868773	rRNA processing in the nucleus and cytosol	3.13	0.00	Rps16, Rpl15, Rps28, Rps9, Fau, Rpl13a, Rps25, Rpl17, Rplp1, Rps15, Rpl37rt, Rpl37, Rps21, Rps4x, Rpl9, Rpl7, Rpl34, Rps3, Rps10, Rpl38, Rpl26, Rpsa, Rpl24, Rpl6, Rps13, ...
R-MMU-72695	Formation of the ternary complex, and subsequently, the 43S complex	2.97	0.00	Rps16, Rps28, Rps9, Fau, Rps25, Rps15, Rps21, Eif3h, Rps4x, Rps3, Rps10, Rpsa, Rps13, Rps27, Rps6, Rps5, Rps8, Rps20, Eif3k, Rps23, Rps19, Rps29, Eif2s2, Rps11, Rps14, ...
R-MMU-72649	Translation initiation complex formation	2.94	0.00	Rps16, Rps28, Rps9, Fau, Rps25, Rps15, Rps21, Eif3h, Rps4x, Rps3, Rps10, Rpsa, Rps13, Rps27, Rps6, Rps5, Rps8, Rps20, Eif3k, Rps23, Rps19, Rps29, Eif2s2, Rps11, Rps14, ...
...	...	...	...	...

Gene Set Enrichment Analysis (GSEA) for Database "Reactome"

mm39 (Mus musculus)

Genes

We identified **156 up-regulated** and **74 down-regulated** terms from Reactome in group "Astrocytes_KIN" compared to group "Astrocytes_WT" (Figure 9). The most significantly up-regulated terms were **L13a-mediated translational silencing of Ceruloplasmin expression, Formation of a pool of free 40S subunits, GTP hydrolysis and joining of the 60S ribosomal subunit** . The most significantly down-regulated terms were **Neuronal System, Transmission across Chemical Synapses, Cardiac conduction** . The most significantly terms are listed in Table 8.

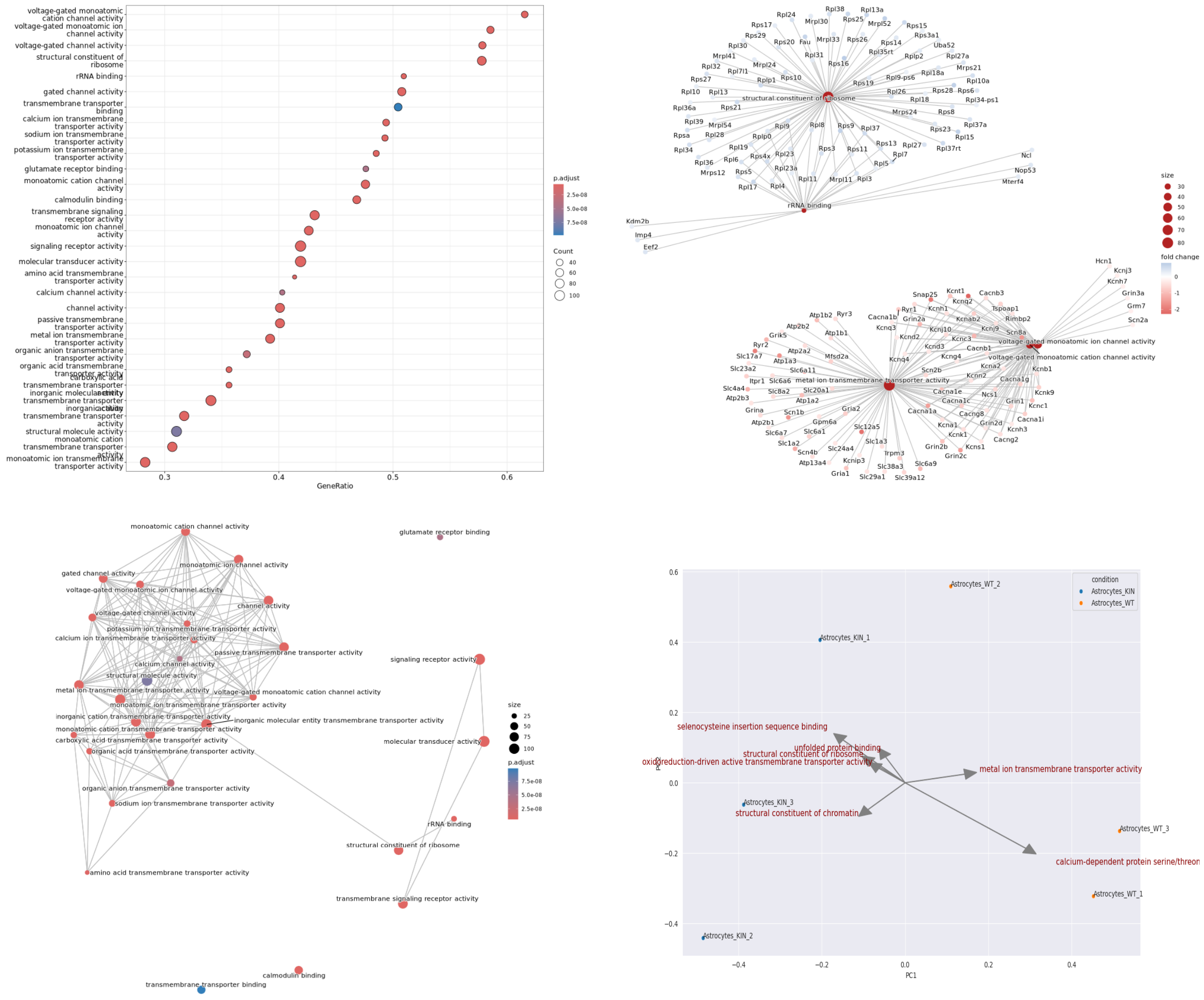

Figure 10. A) Dotplot of enriched pathways. Dots represent enriched pathways, with size indicating gene count and color showing significance (e.g., adjusted p-value). **B)** Cnetplot of gene-pathway relationships. Pathways and their associated genes are connected, with node size and edge thickness indicating enrichment and shared genes. **C)** Enrichment map plot showing overlapping gene sets making it easier to identify functional modules. **D)** PCA biplot showing the average loading of high-variance genes of up to 10 functional modules amongst the enriched terms.

Table 9. Top 15 enriched terms from GO-MF in Astrocytes_KIN compared to Astrocytes_WT. based on gene expression.

ID	Term	NES	Adj. P-Value	Genes
GO:0003735	structural constituent of ribosome	3.37	0.00	Rps16, Rpl15, Mrpl52, Rps28, Rps9, Fau, Rpl13a, Rps25, Rpl17, Rplp1, Rps15, Rpl37rt, Rpl37, Rps21, Rps4x, Rpl9, Rpl7, Rpl34, Rps3, Rpl10a, Rps10, Rpl38, Rpl26, Rpsa, Rpl24, ...
GO:0019843	rRNA binding	2.77	0.00	Rps9, Rpl17, Rpl37, Rps4x, Rpl9, Rpl7, Rps3, Rpl6, Rps13, Rpl19, Rpl3, Rps5, Rpl8, Rpl11, Eef2, Rpl23, Rplp0, Nop53, Rps11, Imp4, Ncl, Rpl4, Mrpl11, Mterf4, Rpl23a, ...
GO:0046873	metal ion transmembrane transporter activity	-2.68	0.00	Kcnq4, Kcnd2, Kcng4, Kcnh3, Atp2b1, Kcnq3, Kcnip3, Kcnd3, Atp13a4, Grik5, Kcns1, Grin2d, Slc24a4, Kcna2, Kcnn2, Atp2b3, Gpm6a, Slc29a1, Rimbp2, Slc6a7, Cacng8, Kcnj9, Kcna1, Slc38a3, Kcnh1, ...
GO:0022843	voltage-gated monoatomic cation channel activity	-2.64	0.00	Kcnq4, Kcnd2, Kcng4, Kcnh3, Kcnq3, Kcnd3, Kcns1, Grin2d, Kcna2, Kcnn2, Rimbp2, Cacng8, Kcnj9, Kcna1, Kcnh1, Kcnb1, Cacnb1, Cacng2, Kcnk1, Cacnb3, Kcnj10, Ryr1, Scn2b, Ncs1, Grin1, ...
GO:0005244	voltage-gated monoatomic ion channel activity	-2.63	0.00	Grin3a, Grm7, Kcnh7, Scn2a, Kcnj3, Hcn1, Kcnq4, Kcnd2, Kcng4, Kcnh3, Kcnq3, Kcnd3, Kcns1, Grin2d, Kcna2, Kcnn2, Rimbp2, Cacng8, Kcnj9, Kcna1, Kcnh1, Kcnb1, Cacnb1, Cacng2, Kcnk1, ...
GO:0022832	voltage-gated channel activity	-2.63	0.00	Grin3a, Grm7, Kcnh7, Scn2a, Kcnj3, Hcn1, Kcnq4, Kcnd2, Kcng4, Kcnh3, Kcnq3, Kcnd3, Kcns1, Grin2d, Kcna2, Kcnn2, Rimbp2, Cacng8, Kcnj9, Kcna1, Kcnh1, Kcnb1, Cacnb1, Cacng2, Kcnk1, ...
GO:0022836	gated channel activity	-2.56	0.00	Grid1, Kcnc2, Gabrb1, Piezo1, Itpr2, Grin3a, Grm7, Kcnh7, Scn2a, Kcnj3, Asic2, Gabrb3, Hcn1, Nalf1, Gabra4, Kcnq4, Kcnd2, Kcng4, Kcnh3, Kcnq3, Kcnd3, Grik5, Kcns1, Grin2d, Kcna2, ...
GO:0015079	potassium ion transmembrane transporter activity	-2.55	0.00	Kcnq4, Kcnd2, Kcng4, Kcnh3, Kcnq3, Kcnip3, Kcnd3, Grik5, Kcns1, Slc24a4, Kcna2, Kcnn2, Kcnj9, Kcna1, Kcnh1, Kcnb1, Kcnk1, Kcnj10, Scn2b, Atp1b1, Kcnk9, Gria2, Kcnab2, Kcnt1, Kcnc1, ...
GO:0015081	sodium ion transmembrane transporter activity	-2.45	0.00	Slc8b1, Asic2, Hcn1, Slc8a1, Slc13a3, Grik5, Slc24a4, Slc29a1, Slc6a7, Slc38a3, Slc23a2, Scn4b, Slc1a3, Kcnk1, Slc6a6, Slc8a2, Scn2b, Slc20a1, Atp1b1, Grin1, Cacna1g, Slc6a1, Mfsd2a, Cacna1i, Gria2, ...
GO:0005261	monoatomic cation channel activity	-2.44	0.00	Pkd1, Piezo1, Itpr2, Grin3a, Grm7, Kcnh7, Scn2a, Kcnj3, Asic2, Hcn1, Nalf1, Atp6v1a, Kcnq4, Kcnd2, Kcng4, Kcnh3, Kcnq3, Kcnip3, Kcnd3, Grik5, Kcns1, Grin2d, Slc24a4, Kcna2, Kcnn2, ...
GO:0015085	calcium ion transmembrane transporter activity	-2.43	0.00	Pkd1, Itpr2, Grin3a, Grm7, Slc8b1, Slc30a10, Slc8a1, Nalf1, Atp2b1, Atp13a4, Grin2d, Slc24a4, Atp2b3, Gpm6a, Rimbp2, Cacng8, Cacnb1, Cacng2, Grina, Atp2a2, Cacnb3, Ryr3, Slc8a2, Ryr1, Ncs1, ...
GO:0005516	calmodulin binding	-2.43	0.00	Sptbn1, Cask, Pde1c, Grm7, Scn2a, Map2, Epb41, Add3, Slc8a1, Pla2g6, Camsap1, Ppp3ca, Atp2b1, Kcnq3, Slc24a4, Ttn, Phkg1, Egfr, Sptan1, Adcy3, Akap12, Map6, Kcnn2, Camkk1, Phka1, ...
GO:0005342	organic acid transmembrane transporter activity	-2.40	0.00	Grik5, Slc1a4, Grin2d, Slc3a2, Nherf1, Slc16a11, Slc6a7, Slc38a3, Slc23a2, Slc7a11, Sfxn5, Slc1a3, Slc6a6, Slc27a1, Slc25a22, Slco1c1, Slc2a1, Slc25a18, Grin1, Slc6a1, Mfsd2a, Slc7a10, Gria2, Gria1, Grin2b, ...
GO:0046943	carboxylic acid transmembrane transporter activity	-2.40	0.00	Grik5, Slc1a4, Grin2d, Slc3a2, Nherf1, Slc16a11, Slc6a7, Slc38a3, Slc23a2, Slc7a11, Sfxn5, Slc1a3, Slc6a6, Slc27a1, Slc25a22, Slco1c1, Slc2a1, Slc25a18, Grin1, Slc6a1, Mfsd2a, Slc7a10, Gria2, Gria1, Grin2b, ...
GO:0005216	monoatomic ion channel activity	-2.38	0.00	Gabrb1, Pkd1, Piezo1, Itpr2, Grin3a, Grm7, Kcnh7, Scn2a, Kcnj3, Asic2, Gabrb3, Slc26a6, Hcn1, Nalf1, Gabra4, Atp6v1a, Kcnq4, Kcnd2, Kcng4, Kcnh3, Kcnq3, Kcnip3, Kcnd3, Grik5, Slc1a4, ...
...	...	...	...	...

Gene Set Enrichment Analysis (GSEA) for Database "GO-MF"

mm39 (Mus musculus)

Genes

We identified **27 up-regulated** and **143 down-regulated** terms from GO-MF in group "Astrocytes_KIN" compared to group "Astrocytes_WT" (Figure 10). The most significantly up-regulated terms were **structural constituent of ribosome, rRNA binding, structural molecule activity** . The most significantly down-regulated terms were **metal ion transmembrane transporter activity, voltage-gated monoatomic cation channel activity, voltage-gated monoatomic ion channel activity** . The most significantly terms are listed in Table 9.

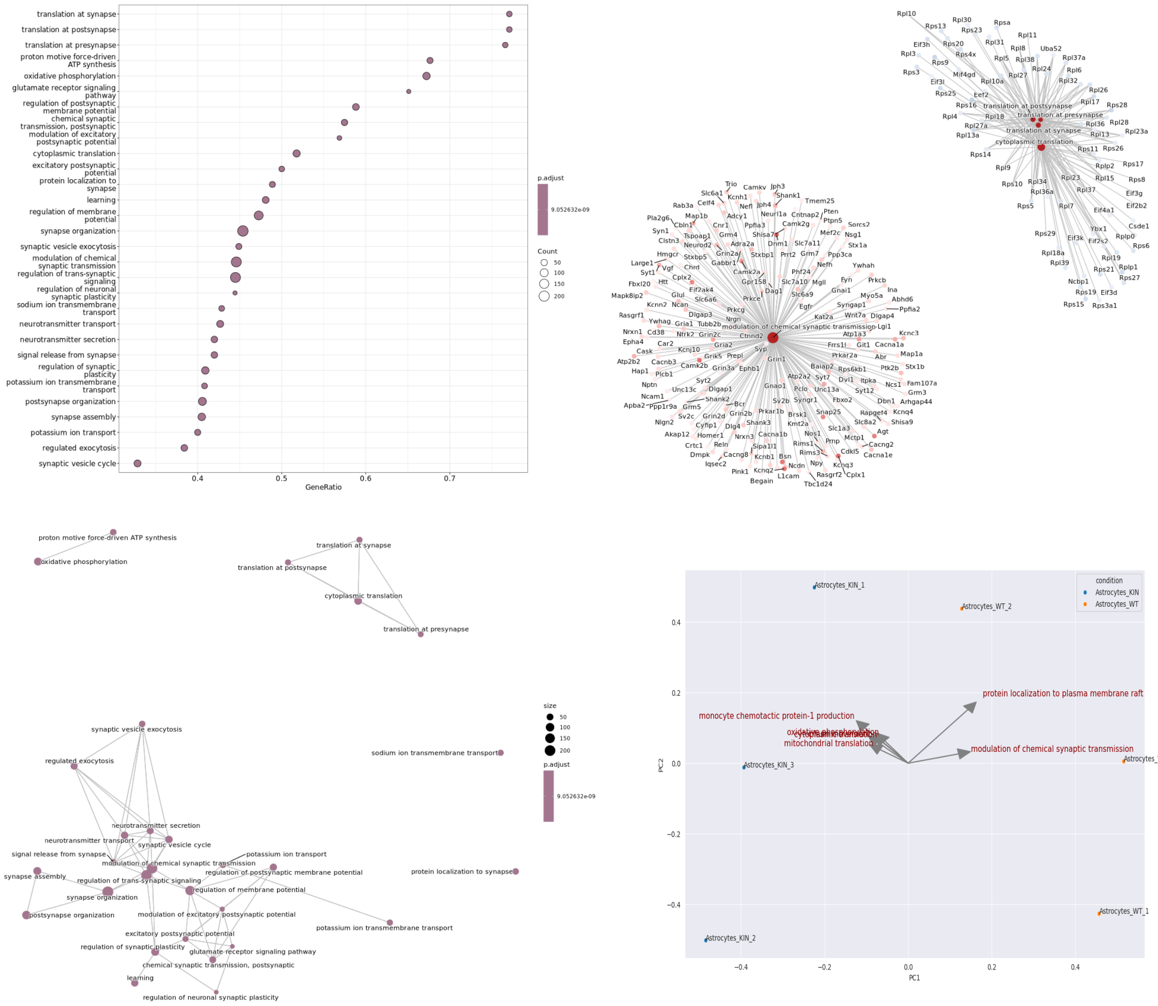

Figure 11. A) Dotplot of enriched pathways. Dots represent enriched pathways, with size indicating gene count and color showing significance (e.g., adjusted p-value). **B)** Cnetplot of gene-pathway relationships. Pathways and their associated genes are connected, with node size and edge thickness indicating enrichment and shared genes. **C)** Enrichment map plot showing overlapping gene sets making it easier to identify functional modules. **D)** PCA biplot showing the average loading of high-variance genes of up to 10 functional modules amongst the enriched terms.

Table 10. Top 15 enriched terms from GO-BP in Astrocytes_KIN compared to Astrocytes_WT. based on gene expression.

ID	Term	NES	Adj. P-Value	Genes
GO:0002181	cytoplasmic translation	3.15	0.00	Rps16, Rpl15, Rps28, Rps9, Rpl13a, Rps25, Rpl17, Rplp1, Rps15, Rpl37, Rps21, Eif3h, Rps4x, Rpl9, Rpl7, Rpl34, Rps3, Rpl10a, Rps10, Rpl38, Rpl26, Rpsa, Rpl24, Rpl6, Rps13, ...
GO:0140241	translation at synapse	3.11	0.00	Rps16, Rpl15, Rps28, Rpl13a, Rpl17, Rpl37, Rpl9, Rpl7, Rpl34, Rpl10a, Rps10, Rpl38, Rpl26, Rpl24, Rpl6, Rpl27, Rps27, Rpl36, Rpl37a, Rps5, Rpl8, Rpl28, Rpl32, Eef2, Rpl23, ...
GO:0140242	translation at postsynapse	3.11	0.00	Rps16, Rpl15, Rps28, Rpl13a, Rpl17, Rpl37, Rpl9, Rpl7, Rpl34, Rpl10a, Rps10, Rpl38, Rpl26, Rpl24, Rpl6, Rpl27, Rps27, Rpl36, Rpl37a, Rps5, Rpl8, Rpl28, Rpl32, Eef2, Rpl23, ...
GO:0140236	translation at presynapse	3.09	0.00	Rps16, Rpl15, Rps28, Rpl13a, Rpl17, Rpl37, Rpl9, Rpl7, Rpl34, Rpl10a, Rps10, Rpl38, Rpl26, Rpl24, Rpl6, Rpl27, Rps27, Rpl36, Rpl37a, Rps5, Rpl8, Rpl28, Rpl32, Rpl23, Rpl27a, ...
GO:0050804	modulation of chemical synaptic transmission	-2.73	0.00	Rab3a, Rps6kb1, Cd38, Fbxl20, Cntnap2, Cyfip1, Cask, Grin3a, Grm7, Stxbp5, Dag1, Crtc1, Eif2ak4, Mctp1, Cdkl5, Begain, Syp, Apba2, Kmt2a, Stx1a, Prkar2a, Fyn, Arhgap44, Chrd, Pla2g6, ...
GO:0099177	regulation of trans-synaptic signaling	-2.73	0.00	Rab3a, Rps6kb1, Cd38, Fbxl20, Cntnap2, Cyfip1, Cask, Grin3a, Grm7, Stxbp5, Dag1, Crtc1, Eif2ak4, Mctp1, Cdkl5, Begain, Syp, Apba2, Kmt2a, Stx1a, Prkar2a, Fyn, Arhgap44, Chrd, Pla2g6, ...
GO:0006836	neurotransmitter transport	-2.68	0.00	Git1, Sv2c, Brsk1, Ppfia2, Grik5, Prrt2, Mef2c, Ntrk2, Syn1, Ptprn2, Ppp1r9a, Npy, Ppfia3, Syt12, Stx1b, Slc29a1, Rimbp2, Syt2, Slc6a7, Kcnh1, Dgki, Unc13c, Cadps, Nrxn3, Synj1, ...
GO:0048167	regulation of synaptic plasticity	-2.65	0.00	Reln, Dbn1, Shank2, Nptn, Epha4, Pten, Kcnq3, Mgll, Brsk1, Abhd6, Grin2d, Prrt2, Mef2c, Ntrk2, Syn1, Syngap1, Kat2a, Vgf, Kcnn2, Ppp1r9a, Htt, Ppfia3, Syt12, Prnp, Nsg1, ...
GO:0060078	regulation of postsynaptic membrane potential	-2.63	0.00	Mecp2, Gabrb1, Atxn1, Cntnap2, Grin3a, Abat, Begain, Gabrb3, Stx1a, Hcn1, Gabra4, Tmem25, Reln, Dvl1, Dbn1, Ppp3ca, Kcnd2, Pten, Tbc1d24, Grik5, Grin2d, Cx3cl1, Mef2c, Kcna2, Ppp1r9a, ...
GO:0007269	neurotransmitter secretion	-2.61	0.00	Git1, Sv2c, Brsk1, Ppfia2, Grik5, Prrt2, Mef2c, Ntrk2, Syn1, Ptprn2, Ppp1r9a, Npy, Ppfia3, Syt12, Stx1b, Rimbp2, Syt2, Kcnh1, Dgki, Unc13c, Cadps, Nrxn3, Synj1, Rims3, Gpr158, ...
GO:0099643	signal release from synapse	-2.61	0.00	Git1, Sv2c, Brsk1, Ppfia2, Grik5, Prrt2, Mef2c, Ntrk2, Syn1, Ptprn2, Ppp1r9a, Npy, Ppfia3, Syt12, Stx1b, Rimbp2, Syt2, Kcnh1, Dgki, Unc13c, Cadps, Nrxn3, Synj1, Rims3, Gpr158, ...
GO:0006119	oxidative phosphorylation	2.59	0.00	Ndufa3, Park7, Cox6b2, Ndufb9, Atp5f1e, Chchd2, Cox6b1, Ndufa7, Atp5if1, Atp5f1d, Ndufa12, Ndufa5, Uqcr11, Nipsnap2, Atp5po, Ndufa13, Ndufv2, Ndufb8, Uqcc2, Ndufs2, Cox8a, Atp5mf, Atp5pd, Cox4i1, Ndufs8, ...
GO:0016079	synaptic vesicle exocytosis	-2.59	0.00	Dvl1, Git1, Sv2c, Brsk1, Ppfia2, Grik5, Prrt2, Syn1, Npy, Ppfia3, Syt12, Stx1b, Rimbp2, Syt2, Kcnh1, Unc13c, Cadps, Synj1, Rims3, Atp2a2, Prkcg, Sv2b, Ncs1, Prepl, Vamp1, ...
GO:0007215	glutamate receptor signaling pathway	-2.58	0.00	Grik5, Grin2d, Cx3cl1, Mef2c, Homer1, Prnp, Grm3, Kcnb1, Frrs1l, Plcb1, Grm5, Nrxn1, Grin1, Gria2, Dlg4, Grm4, Cdk5r1, Tiam1, Gria1, Grin2b, Shank3, Grin2a, Ptk2b, Kalrn, Unc13a, ...
GO:0035418	protein localization to synapse	-2.57	0.00	Vps26b, Dag1, Olfm2, Tnik, Dlg2, Asic2, Rab4a, Arhgap44, Reln, Magi2, Git1, Rapgef4, Homer1, Lrrc7, Stx1b, Nsg1, Dlg3, Klc1, Nlgn2, Nrxn3, Cacng2, Nbea, Iqsec2, Map1a, Lgi1, ...
...	...	...	...	...

Gene Set Enrichment Analysis (GSEA) for Database "GO-BP"

mm39 (Mus musculus)

Genes

We identified **105 up-regulated** and **745 down-regulated** terms from GO-BP in group "Astrocytes_KIN" compared to group "Astrocytes_WT" (Figure 11). The most significantly up-regulated terms were **cytoplasmic translation, translation at synapse, translation at postsynapse** . The most significantly down-regulated terms were **modulation of chemical synaptic transmission, regulation of trans-synaptic signaling, neurotransmitter transport** . The most significantly terms are listed in Table 10.

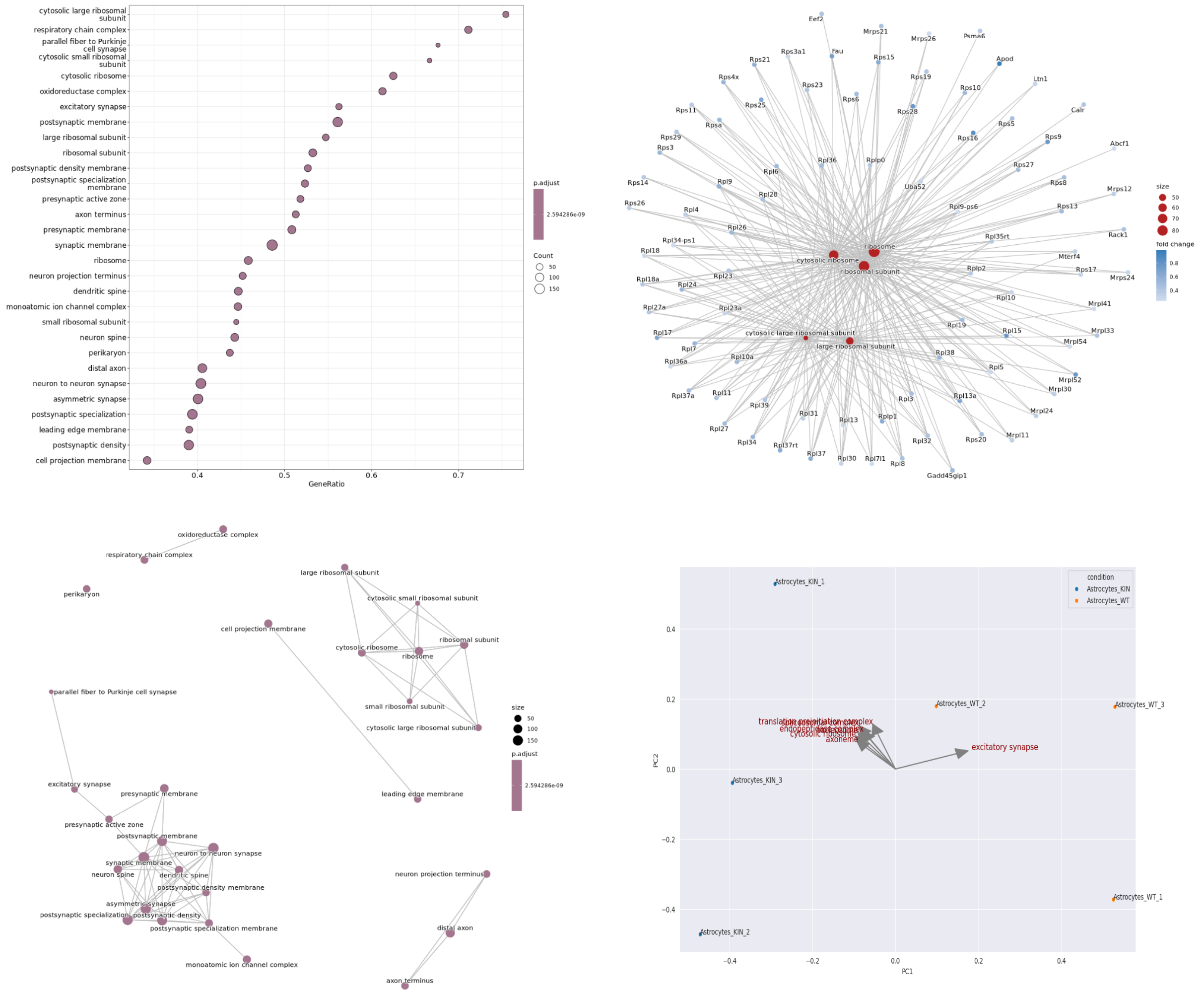

Figure 12. A) Dotplot of enriched pathways. Dots represent enriched pathways, with size indicating gene count and color showing significance (e.g., adjusted p-value). **B)** Cnetplot of gene-pathway relationships. Pathways and their associated genes are connected, with node size and edge thickness indicating enrichment and shared genes. **C)** Enrichment map plot showing overlapping gene sets making it easier to identify functional modules. **D)** PCA biplot showing the average loading of high-variance genes of up to 10 functional modules amongst the enriched terms.

Table 11. Top 15 enriched terms from GO-CC in Astrocytes_KIN compared to Astrocytes_WT. based on gene expression.

ID	Term	NES	Adj. P-Value	Genes
GO:0022626	cytosolic ribosome	3.39	0.00	Apod, Rps16, Rpl15, Rps28, Rps9, Fau, Rpl13a, Rps25, Rpl17, Rplp1, Rps15, Rpl37rt, Rpl37, Rps21, Rps4x, Rpl9, Rpl7, Rpl34, Rps3, Rpl10a, Rps10, Rpl38, Rpl26, Rpsa, Rpl24, ...
GO:0044391	ribosomal subunit	3.35	0.00	Rps16, Rpl15, Mrpl52, Rps28, Rps9, Fau, Rpl13a, Rps25, Rpl17, Rplp1, Rps15, Rpl37rt, Rpl37, Rps21, Rps4x, Rpl9, Rpl7, Rpl34, Rps3, Gadd45gip1, Rpl10a, Rps10, Rpl38, Rpl26, Rpsa, ...
GO:0005840	ribosome	3.22	0.00	Apod, Rps16, Rpl15, Mrpl52, Rps28, Rps9, Fau, Rpl13a, Rps25, Rpl17, Rplp1, Rps15, Rpl37rt, Rpl37, Rps21, Rps4x, Rpl9, Rpl7, Rpl34, Rps3, Gadd45gip1, Rpl10a, Rps10, Rpl38, Rpl26, ...
GO:0022625	cytosolic large ribosomal subunit	3.17	0.00	Rpl15, Rpl13a, Rpl17, Rplp1, Rpl37rt, Rpl37, Rpl9, Rpl7, Rpl34, Rpl10a, Rpl38, Rpl26, Rpl24, Rpl6, Rpl27, Rpl19, Rpl36, Rpl37a, Rpl3, Rpl8, Rpl28, Rpl11, Rpl32, Rpl39, Rpl23, ...
GO:0015934	large ribosomal subunit	3.06	0.00	Rpl15, Mrpl52, Rpl13a, Rpl17, Rplp1, Rpl37rt, Rpl37, Rpl9, Rpl7, Rpl34, Gadd45gip1, Rpl10a, Rpl38, Rpl26, Rpl24, Rpl6, Rpl27, Rpl19, Rpl36, Rpl37a, Rpl3, Rpl8, Rpl28, Rpl11, Rpl32, ...
GO:0022627	cytosolic small ribosomal subunit	2.90	0.00	Rps16, Rps28, Rps9, Fau, Rps25, Rps15, Rps21, Rps4x, Rps3, Rps10, Rpsa, Rps13, Rps27, Rps6, Rps5, Rps8, Rps20, Rps23, Rps19, Rps29, Rps11, Rps14, Rps26, Rps17
GO:0015935	small ribosomal subunit	2.80	0.00	Rps16, Rps28, Rps9, Fau, Rps25, Rps15, Rps21, Rps4x, Rps3, Rps10, Rpsa, Rps13, Rps27, Rps6, Rps5, Rps8, Rps20, Mrps21, Rack1, Rps23, Rps19, Rps29, Rps11, Rps14, Rps26, ...
GO:0060076	excitatory synapse	-2.77	0.00	Dbn1, Git1, Mgll, Susd4, Ntm, Adgrb3, Rapgef4, Ntrk2, Homer1, Atp2b3, Gpm6a, Cbln3, Cadm3, Kcnj9, Kcnh1, Dgki, Nlgn2, Unc13c, Shisa7, Synj1, Ywhah, Cbln1, Plxna4, Baiap2, Nrxn1, ...
GO:0098803	respiratory chain complex	2.75	0.00	Ndufa3, Cox6b2, Ndufb9, Atp5f1e, Cox6b1, Ndufa7, Atp5f1d, Ndufa12, Ndufa5, Uqcr11, Atp5po, Ndufa13, Ndufv2, Ndufb8, Ndufs2, Cox8a, Atp5mf, Atp5mg, Atp5pd, Cox4i1, Ndufs8, Ndufa11, Ndufv1, Ndufa2, Atp5f1b, ...
GO:0097060	synaptic membrane	-2.70	0.00	Grid1, Hip1, Igsf9b, Rgs9, Kcnc2, Olfm1, Apba1, Otof, Usp48, Gabrb1, Pdlim4, Fcho2, Cntnap2, Septin7, Cask, Grin3a, Grm7, Scn2a, Stxbp5, Cnih2, Kcnj3, Ache, Dag1, Olfm2, Sigmar1, ...
GO:0045211	postsynaptic membrane	-2.60	0.00	Pcdh10, Grik2, Dnm3, Akap1, Ptprt, Cadps2, Fxyd6, Dlg1, Sorcs3, Ctnnb1, Ptprs, Ank3, Cadm1, Fbxo45, Plppr4, Plxnb1, Slitrk2, Efnb2, Asic1, Tenm2, Gpr179, Lrp8, Grid1, Hip1, Igsf9b, ...
GO:0043197	dendritic spine	-2.58	0.00	Asic2, Apba2, Palm, Arhgap44, Slc8a1, Fbxo2, Asap1, Septin11, Dvl1, Abi2, Dbn1, Ppp3ca, Als2, Kcnd2, Shank2, Bcr, Epha4, Pten, Atp2b1, Kcnd3, Ppfia2, Prrt2, Hap1, Rapgef4, Ntrk2, ...
GO:0042734	presynaptic membrane	-2.57	0.00	Hip1, Rgs9, Kcnc2, Apba1, Otof, Gabrb1, Fcho2, Cntnap2, Septin7, Cask, Grm7, Scn2a, Stxbp5, Kcnj3, Ache, Syp, Napb, Stx1a, Adam23, Hcn1, Nectin1, Ncam1, Flrt2, Gad2, Nptn, ...
GO:0044309	neuron spine	-2.56	0.00	Syp, Asic2, Apba2, Palm, Arhgap44, Slc8a1, Fbxo2, Asap1, Septin11, Dvl1, Abi2, Dbn1, Ppp3ca, Als2, Kcnd2, Shank2, Bcr, Epha4, Pten, Atp2b1, Kcnd3, Ppfia2, Prrt2, Hap1, Rapgef4, ...
GO:0044306	neuron projection terminus	-2.54	0.00	Hcn1, Slc8a1, Ptprn, Flrt1, Git1, Epha4, Kcnip3, Tbc1d24, Grik5, Prrt2, Hap1, Rapgef4, Ap3d1, Ntrk2, Kcna2, Ptprn2, Npy, Atcay, Prnp, Rimbp2, Apc, Gad1, Kcna1, Dgki, Unc13c, ...
...	...	...	...	...

Gene Set Enrichment Analysis (GSEA) for Database "GO-CC"

mm39 (Mus musculus)

Genes

We identified **79 up-regulated** and **153 down-regulated** terms from GO-CC in group "Astrocytes_KIN" compared to group "Astrocytes_WT" (Figure 12). The most significantly up-regulated terms were **cytosolic ribosome, ribosomal subunit, ribosome** . The most significantly down-regulated terms were **excitatory synapse, synaptic membrane, postsynaptic membrane** . The most significantly terms are listed in Table 11.

Microglia_KIN vs. Microglia_WT

Table 12. Top 20 differentially expressed gene in Microglia_KIN compared to Microglia_WT based on log2 fold change standard error.

Gene	Description	Mean	Log2 Fold Change	Standard Error	P-Value	Adj. P-Value
Apoe	apolipoprotein E [Source:MGI Symbol;Acc:MGI:88057]	1,959.91	3.00	0.37	0.00	0.00
Bc1-201	brain cytoplasmic RNA 1 [Source:MGI Symbol;Acc:MGI:104905]	2,341.52	-1.23	0.39	0.00	0.01
Csf1	colony stimulating factor 1 (macrophage) [Source:MGI Symbol;Acc:MGI:1339753]	57.02	1.79	0.41	0.00	0.00
Cd63	CD63 antigen [Source:MGI Symbol;Acc:MGI:99529]	95.93	1.61	0.41	0.00	0.00
Axl	AXL receptor tyrosine kinase [Source:MGI Symbol;Acc:MGI:1347244]	43.32	2.52	0.44	0.00	0.00
Clec7a	C-type lectin domain family 7, member a [Source:MGI Symbol;Acc:MGI:1861431]	38.70	2.71	0.44	0.00	0.00
Rian-209	RNA imprinted and accumulated in nucleus [Source:MGI Symbol;Acc:MGI:1922995]	124.25	-2.53	0.56	0.00	0.00
ENSMUST00020183598	nan	114.28	-3.13	0.56	0.00	0.00
Itgax	integrin alpha X [Source:MGI Symbol;Acc:MGI:96609]	22.44	3.35	0.60	0.00	0.00
Il1b	interleukin 1 beta [Source:MGI Symbol;Acc:MGI:96543]	46.88	1.42	0.64	0.00	0.02
Mpeg1	macrophage expressed gene 1 [Source:MGI Symbol;Acc:MGI:1333743]	69.03	1.30	0.70	0.00	0.02
Mir326-201	microRNA 326 [Source:MGI Symbol;Acc:MGI:3619338]	63.78	-1.72	0.72	0.00	0.02
Bsn	bassoon [Source:MGI Symbol;Acc:MGI:1277955]	18.39	-2.47	0.73	0.00	0.01
Cst7	cystatin F (leukocystatin) [Source:MGI Symbol;Acc:MGI:1298217]	20.28	3.65	0.75	0.00	0.00
Gpnmb	glycoprotein (transmembrane) nmb [Source:MGI Symbol;Acc:MGI:1934765]	30.14	4.83	0.85	0.00	0.00
Rian-201	RNA imprinted and accumulated in nucleus [Source:MGI Symbol;Acc:MGI:1922995]	16.27	-2.91	0.87	0.00	0.00
Map1a	microtubule-associated protein 1 A [Source:MGI Symbol;Acc:MGI:1306776]	28.35	-2.17	0.88	0.00	0.01
Fam20c	family with sequence similarity 20, member C [Source:MGI Symbol;Acc:MGI:2136853]	16.53	1.80	0.88	0.00	0.02
Plec	plectin [Source:MGI Symbol;Acc:MGI:1277961]	21.83	-1.63	0.92	0.00	0.03
Sptbn2	spectrin beta, non-erythrocytic 2 [Source:MGI Symbol;Acc:MGI:1313261]	13.49	-3.13	0.93	0.00	0.00
...	...	...	...	...	...	...

Differential Expression Analysis (DEA)

mm39 (Mus musculus)

Genes

In total, we identified **11 over-expressed** genes and **11 under-expressed** genes in group "Microglia_KIN" compared to group "Microglia_WT". Differentially expressed genes were strictly filtered by mean, log2 fold change and standard error to further increase specificity of down-stream analyses. The most significantly differentially expressed genes were **Apoe, Bc1-201, Csf1, Cd63, Axl, Clec7a, Rian-209, ENSMUST00020183598, Itgax, Il1b** (Table 12).

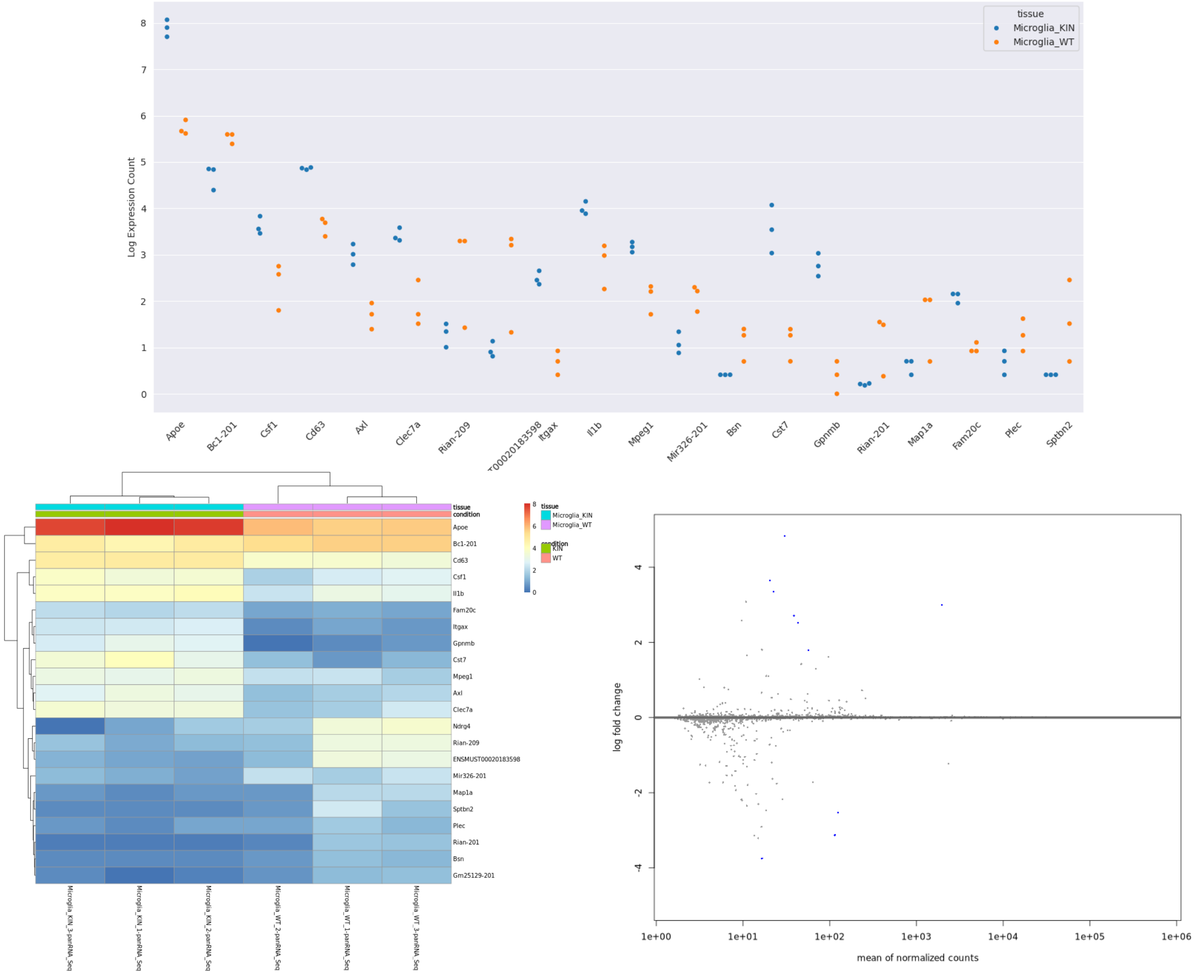

Figure 13. A) Swarm plot showing the log-TPM expression of the genes with the lowest log2 fold change standard error (lfcSE) from the list of significantly differentially expressed genes. **B)** Heatmap showing the log-TPM expression of the genes with the lowest log2 fold change standard error (lfcSE) from the list of significantly differentially expressed genes. **C)** MA plot of differentially expressed genes. The MA plot displays the log2 fold change (y-axis) versus the mean expression (x-axis) for all genes. Significantly differentially expressed genes are highlighted, with points above or below the centerline indicating up-regulated or down-regulated genes. This plot provides insights into the global expression changes and the extent of variability across the feature.dataset.

We created a swarm plot with the standardized and log-transformed expression counts to visually identify false-positive calls (Figure 13A). Using hierarchical clustering on the counts of the differentially expressed genes we **identified clusters** matching the variable Condition with a normalized mutual information (NMI) value of *0.81* (Figure 13B). and we **identified clusters** matching the variable Tissue with a normalized mutual information (NMI) value of *0.81* (Figure 13B). The global distribution of log2FoldChanges in relation to mean expression is shown in (Figure 13C).

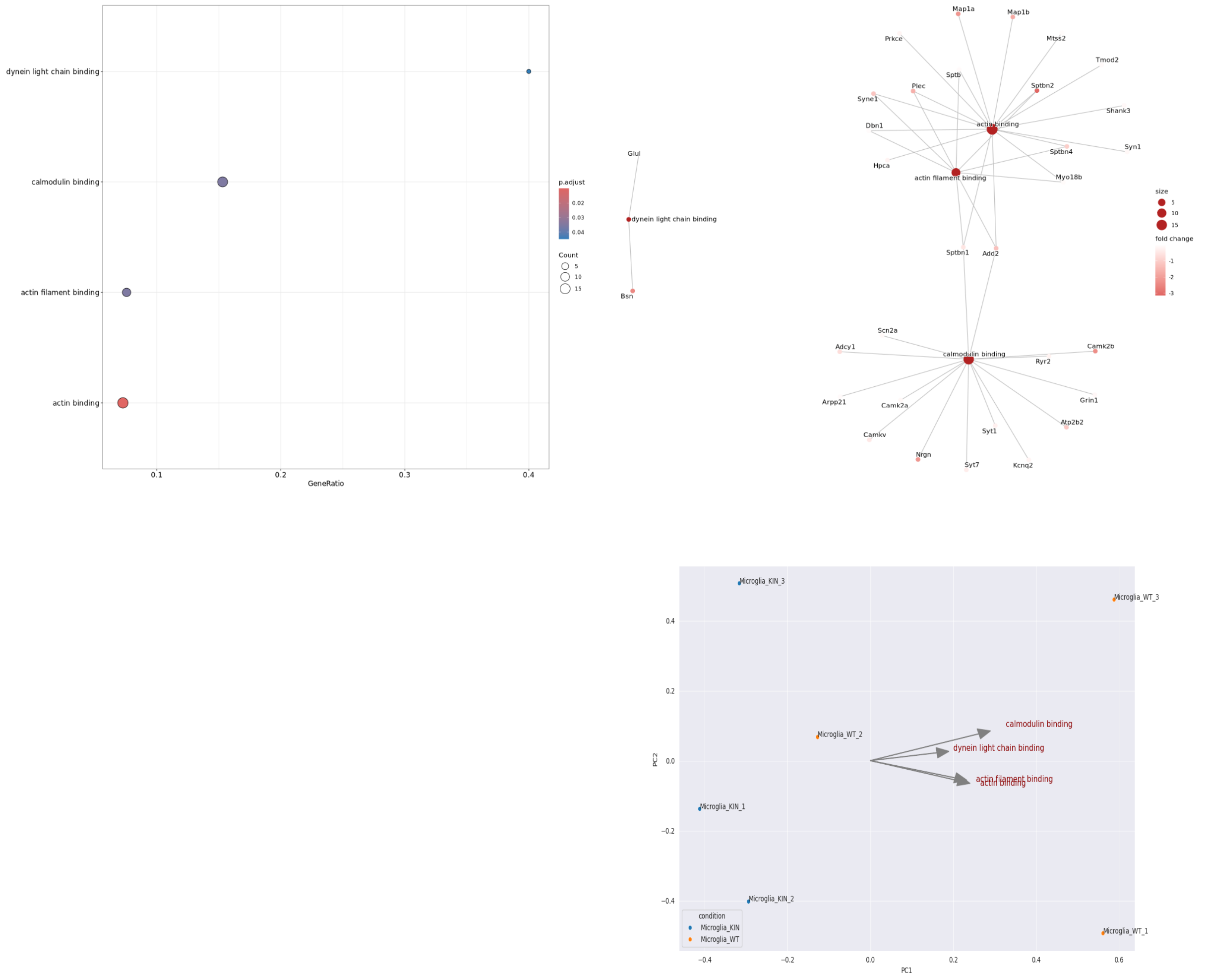

Figure 14. A) Dotplot of enriched pathways. Dots represent enriched pathways, with size indicating gene count and color showing significance (e.g., adjusted p-value). **B)** Cnetplot of gene-pathway relationships. Pathways and their associated genes are connected, with node size and edge thickness indicating enrichment and shared genes. **C)** Enrichment map plot showing overlapping gene sets making it easier to identify functional modules. **D)** PCA biplot showing the average loading of high-variance genes of up to 10 functional modules amongst the enriched terms.

Table 13. Top 4 enriched terms from GO-MF in Microglia_KIN compared to Microglia_WT. based on gene expression.

ID	Term	NES	Adj. P-Value	Genes
GO:0003779	actin binding	-1.55	0.01	Mtss2, Dbn1, Myo18b, Sptb, Tmod2, Shank3, Syn1, Prkce, Hpca, Sptbn1, Sptbn4, Syne1, Add2, Plec, Map1b, Map1a, Sptbn2
GO:0005516	calmodulin binding	-1.48	0.04	Arpp21, Scn2a, Camk2a, Grin1, Kcnq2, Ryr2, Syt1, Syt7, Camkv, Sptbn1, Adcy1, Atp2b2, Add2, Nrgn, Camk2b
GO:0051015	actin filament binding	-1.48	0.04	Dbn1, Myo18b, Sptb, Sptbn1, Sptbn4, Syne1, Add2, Plec, Sptbn2
GO:0045503	dynein light chain binding	-1.50	0.04	Glul, Bsn

Gene Set Enrichment Analysis (GSEA) for Database "GO-MF"

mm39 (Mus musculus)

Genes

We identified **0 up-regulated** and **4 down-regulated** terms from GO-MF in group "Microglia_KIN" compared to group "Microglia_WT" (Figure 14). The most significantly down-regulated terms were **actin binding, calmodulin binding, actin filament binding** . The most significantly terms are listed in Table 13.

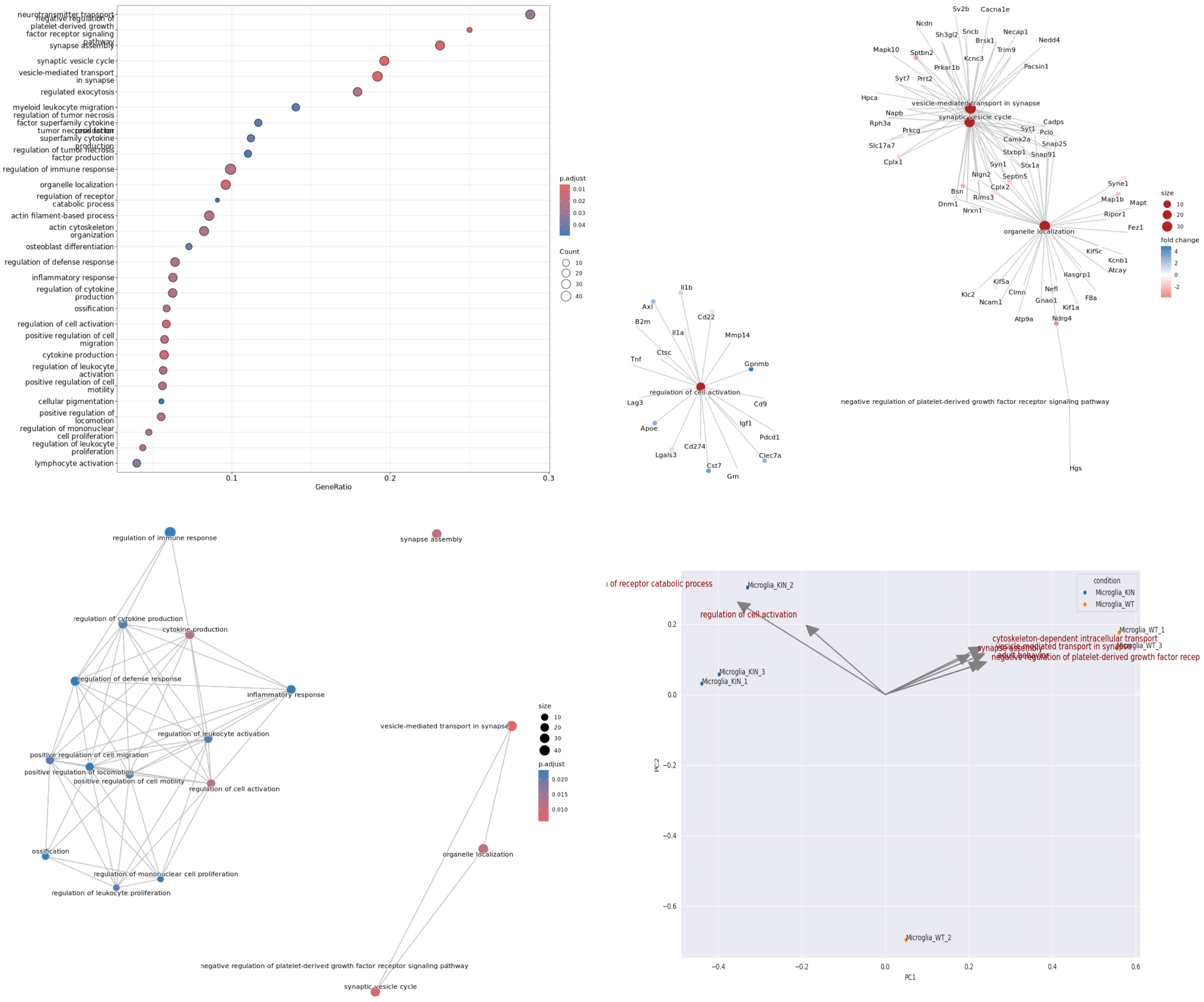

Figure 15. A) Dotplot of enriched pathways. Dots represent enriched pathways, with size indicating gene count and color showing significance (e.g., adjusted p-value). **B)** Cnetplot of gene-pathway relationships. Pathways and their associated genes are connected, with node size and edge thickness indicating enrichment and shared genes. **C)** Enrichment map plot showing overlapping gene sets making it easier to identify functional modules. **D)** PCA biplot showing the average loading of high-variance genes of up to 10 functional modules amongst the enriched terms.

Table 14. Top 15 enriched terms from GO-BP in Microglia_KIN compared to Microglia_WT. based on gene expression.

ID	Term	NES	Adj. P-Value	Genes
GO:0099003	vesicle-mediated transport in synapse	-1.52	0.01	Necap1, Cacna1e, Sv2b, Mapk10, Nrxn1, Nedd4, Snap91, Trim9, Nlgn2, Brsk1, Stx1a, Prkar1b, Syn1, Pacsin1, Camk2a, Kcnc3, Prrt2, Sh3gl2, Napb, Rph3a, Hpca, Cadps, Prkcg, Slc17a7, Pclo, ...
GO:0010642	negative regulation of platelet-derived growth factor receptor signaling pathway	-1.52	0.01	Hgs, Ndrg4
GO:0099504	synaptic vesicle cycle	-1.52	0.01	Nrxn1, Snap91, Trim9, Nlgn2, Brsk1, Stx1a, Prkar1b, Syn1, Pacsin1, Camk2a, Kcnc3, Prrt2, Sh3gl2, Napb, Rph3a, Cadps, Prkcg, Slc17a7, Pclo, Syt1, Syt7, Rims3, Stxbp1, Snap25, Sncb, ...
GO:0050865	regulation of cell activation	1.70	0.01	Gpnmb, Cst7, Apoe, Clec7a, Axl, Il1b, Lgals3, Cd22, B2m, Igf1, Lag3, Pdcd1, Tnf, Mmp14, Cd9, Ctsc, Il1a, Cd274, Grn
GO:0051640	organelle localization	-1.52	0.01	F8a, Kif5c, Clmn, Atp9a, Nrxn1, Snap91, Fez1, Ripor1, Gnao1, Nlgn2, Kcnb1, Stx1a, Nefl, Syn1, Rasgrp1, Klc2, Camk2a, Cadps, Ncam1, Atcay, Mapt, Pclo, Syt1, Rims3, Stxbp1, ...
GO:0007416	synapse assembly	-1.49	0.01	Dlg4, Ntrk3, Caskin1, Nrxn1, Srgap3, Dclk1, Prickle2, Ntrk2, Nlgn2, Nptx1, Arhgap33, Cyfip2, Shank3, Cbln1, Adgrl1, Grin1, Clstn3, Nptxr, Mapt, Adgrb2, Pclo, Lrrc4b, Srcin1, Nrxn2, Snap25, ...
GO:0001816	cytokine production	1.66	0.01	Gpnmb, Clec7a, Axl, Lpl, Il1b, Serpine1, B2m, Igf1, Oas1a, Lag3, Ccl4, Cd14, Zc3hav1, Tnf, Tmem106a, Oas1g, Pld3, H2-Q9, Ccl3, Tlr2, Il1a, Cd274, Ly9, Siglec1, Bst2, ...
GO:2000147	positive regulation of cell motility	1.66	0.02	Gpnmb, Itgax, Clec7a, Csf1, Il1b, Lgals3, Cxcl16, Serpine1, Plau, Igf1, Ccl4, Cxcl10, Mmp14, Ccl3, Tlr2, Il1a, Cd274, Grn
GO:0001817	regulation of cytokine production	1.66	0.02	Gpnmb, Clec7a, Axl, Lpl, Il1b, Serpine1, B2m, Igf1, Oas1a, Lag3, Ccl4, Cd14, Zc3hav1, Tnf, Tmem106a, Oas1g, Pld3, H2-Q9, Ccl3, Tlr2, Il1a, Cd274, Ly9, Siglec1, Bst2, ...
GO:0030335	positive regulation of cell migration	1.66	0.02	Gpnmb, Itgax, Clec7a, Csf1, Il1b, Lgals3, Cxcl16, Serpine1, Plau, Igf1, Ccl4, Cxcl10, Mmp14, Ccl3, Tlr2, Il1a, Cd274, Grn
GO:0002694	regulation of leukocyte activation	1.65	0.02	Gpnmb, Cst7, Clec7a, Axl, Il1b, Lgals3, Cd22, B2m, Igf1, Lag3, Pdcd1, Tnf, Mmp14, Ctsc, Il1a, Cd274, Grn
GO:0070663	regulation of leukocyte proliferation	1.57	0.02	Gpnmb, Csf1, Il1b, Lgals3, Cd22, Igf1
GO:0040017	positive regulation of locomotion	1.67	0.02	Gpnmb, Itgax, Clec7a, Csf1, Il1b, Lgals3, Cxcl16, Serpine1, Plau, Igf1, Ccl4, Cxcl10, Mmp14, Ccl3, Tlr2, Il1a, Cd274, Grn
GO:0006954	inflammatory response	1.66	0.02	Cst7, Apoe, Clec7a, Axl, Csf1, Lpl, Il1b, Serpine1, Igf1, Usp18, Ccl4, Cxcl10, Cd14, Naip6, Tnf, Ctsc, Pld3, Ccl3, Rps19, Tlr2, Il1a, Ctss, Grn, C3
GO:0031347	regulation of defense response	1.62	0.02	Cst7, Apoe, Clec7a, Lpl, Il1b, Serpine1, Igf1, Oas1a, Usp18, Lag3, Cd14, Zc3hav1, Tnf, Ctsc, Oas1g, Ccl3, Rps19, Tlr2, Cd274, Ctss, Grn, C3, Pf4, Ifi204, Eif2ak2, ...
...	...	...	...	...

Gene Set Enrichment Analysis (GSEA) for Database "GO-BP"

mm39 (Mus musculus)

Genes

We identified **24 up-regulated** and **12 down-regulated** terms from GO-BP in group "Microglia_KIN" compared to group "Microglia_WT" (Figure 15). The most significantly up-regulated terms were **regulation of cell activation, cytokine production, positive regulation of cell motility** . The most significantly down-regulated terms were **vesicle-mediated transport in synapse, negative regulation of platelet-derived growth factor receptor signaling pathway, synaptic vesicle cycle** . The most significantly terms are listed in Table 14.

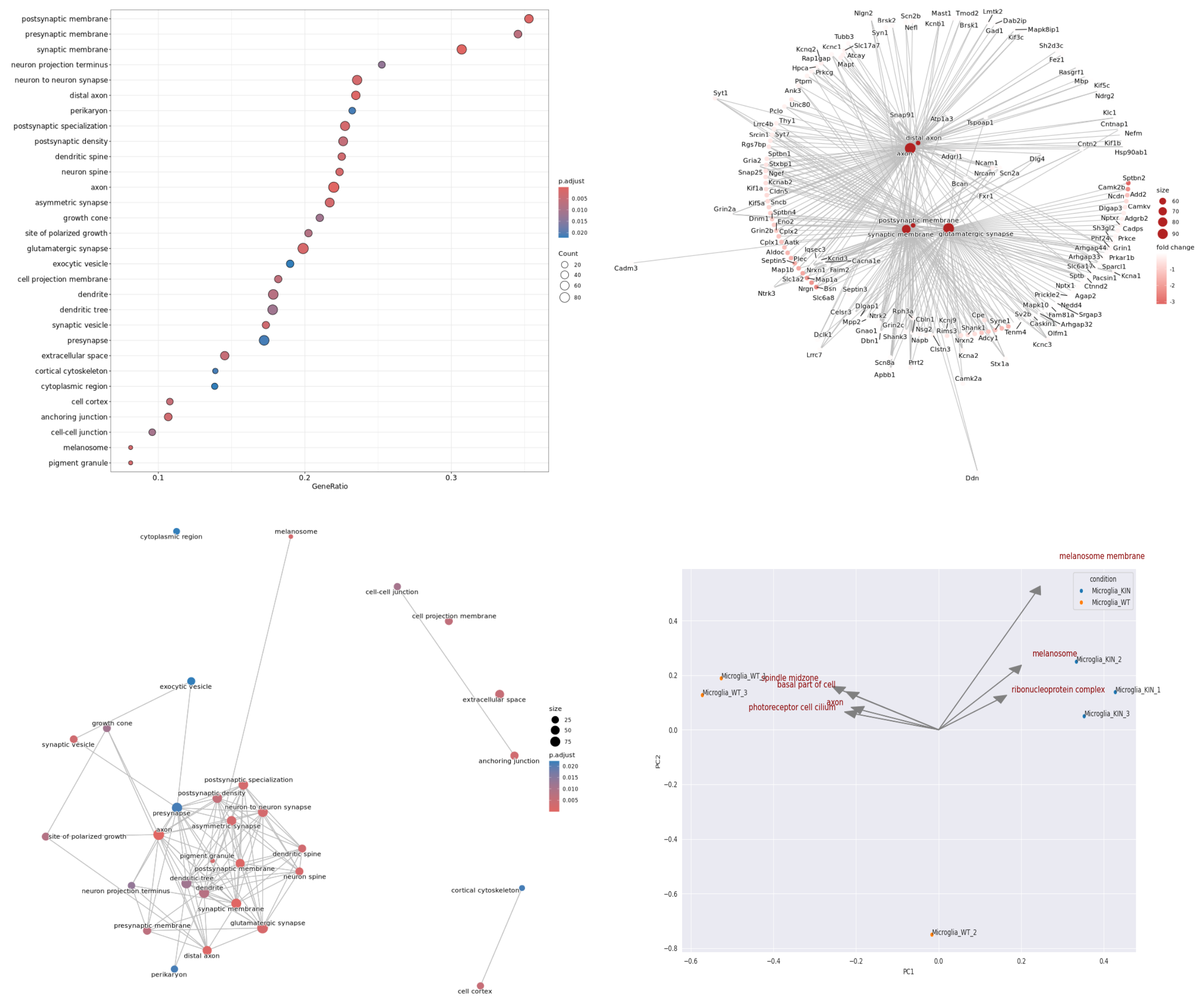

Figure 16. A) Dotplot of enriched pathways. Dots represent enriched pathways, with size indicating gene count and color showing significance (e.g., adjusted p-value). **B)** Cnetplot of gene-pathway relationships. Pathways and their associated genes are connected, with node size and edge thickness indicating enrichment and shared genes. **C)** Enrichment map plot showing overlapping gene sets making it easier to identify functional modules. **D)** PCA biplot showing the average loading of high-variance genes of up to 10 functional modules amongst the enriched terms.

Table 15. Top 15 enriched terms from GO-CC in Microglia_KIN compared to Microglia_WT. based on gene expression.

ID	Term	NES	Adj. P-Value	Genes
GO:0030424	axon	-1.63	0.00	Hsp90ab1, Kif1b, Nefm, Cntnap1, Gria2, Klc1, Tspoap1, Grin2a, Dlg4, Bcan, Ndrg2, Ntrk3, Kif5c, Mbp, Cntn2, Rasgrf1, Nrxn1, Snap91, Fez1, Sh2d3c, Atp1a3, Lrrc7, Dclk1, Scn8a, Ntrk2, ...
GO:0097060	synaptic membrane	-1.56	0.00	Slc6a8, Kcnj9, Gria2, Iqsec3, Kcnd3, Grin2a, Dlg4, Bcan, Cacna1e, Ntrk3, Faim2, Cntn2, Celsr3, Dlgap1, Nrxn1, Mpp2, Snap91, Atp1a3, Lrrc7, Scn8a, Ddn, Gnao1, Cadm3, Ntrk2, Dbn1, ...
GO:0150034	distal axon	-1.52	0.00	Hsp90ab1, Gria2, Klc1, Tspoap1, Grin2a, Ndrg2, Kif5c, Rasgrf1, Nrxn1, Snap91, Fez1, Atp1a3, Dclk1, Ntrk2, Kif3c, Mapk8ip1, Dbn1, Gad1, Lmtk2, Brsk1, Apbb1, Tmod2, Nefl, Olfm1, Brsk2, ...
GO:0045211	postsynaptic membrane	-1.49	0.00	Slc6a8, Gria2, Iqsec3, Kcnd3, Grin2a, Dlg4, Cacna1e, Ntrk3, Faim2, Cntn2, Celsr3, Dlgap1, Mpp2, Snap91, Lrrc7, Scn8a, Ddn, Gnao1, Ntrk2, Dbn1, Nlgn2, Apbb1, Grin2c, Kcnb1, Stx1a, ...
GO:0098978	glutamatergic synapse	-1.52	0.00	Syngap1, Slc6a8, Tenm4, Cntnap1, Gria2, Tspoap1, Grin2a, Dlg4, Bcan, Ntrk3, Sv2b, Caskin1, Arhgap32, Celsr3, Dlgap1, Mapk10, Fam81a, Nrxn1, Mpp2, Srgap3, Nedd4, Lrrc7, Dclk1, Scn8a, Gnao1, ...
GO:0044309	neuron spine	-1.48	0.00	Gria2, Kcnd3, Grin2a, Dlg4, Arhgap32, Mpp2, Nedd4, Atp1a2, Sez6, Atp1a3, Lrrc7, Ddn, Ntrk2, Dbn1, Apbb1, Fxr1, Arhgap33, Arhgap44, Shank3, Camk2a, Grin1, Kcnc3, Prrt2, Rph3a, Hpca, ...
GO:0042470	melanosome	1.65	0.00	Gpnmb, Apoe, Cd63, Ctsb, Mfsd12, Mmp14
GO:0048770	pigment granule	1.65	0.00	Gpnmb, Apoe, Cd63, Ctsb, Mfsd12, Mmp14
GO:0098984	neuron to neuron synapse	-1.51	0.00	Gria1, Akap7, Dnm3, Dynll2, Igsf9b, Spock1, Epb41l1, Syngap1, Nefm, Gria2, Iqsec3, Grin2a, Dlg4, Arhgap32, Celsr3, Dlgap1, Mapk10, Fam81a, Nrxn1, Mpp2, Cit, Snap91, Wwc1, Atp1a3, Lrrc7, ...
GO:0099572	postsynaptic specialization	-1.50	0.00	Igsf9b, Spock1, Epb41l1, Syngap1, Nefm, Gria2, Iqsec3, Kcnd3, Grin2a, Dlg4, Arhgap32, Celsr3, Dlgap1, Mapk10, Fam81a, Mpp2, Cit, Snap91, Wwc1, Lrrc7, Dclk1, Scn8a, Prickle2, Ntrk2, Dbn1, ...
GO:0032279	asymmetric synapse	-1.49	0.00	Igsf9b, Spock1, Epb41l1, Syngap1, Nefm, Gria2, Iqsec3, Grin2a, Dlg4, Arhgap32, Celsr3, Dlgap1, Mapk10, Fam81a, Mpp2, Cit, Snap91, Wwc1, Lrrc7, Dclk1, Scn8a, Prickle2, Ntrk2, Dbn1, Gng3, ...
GO:0070161	anchoring junction	-1.50	0.00	Ppfia1, Tnk2, Pdxp, Ank2, Coro2b, Vapa, Syne2, Tns2, Gria1, Tmem204, Il16, Magi3, Cntnap1, Cdc42bpa, Dlg4, Nrxn1, Mpp2, Pcdh1, Atp1a2, Lrrc7, Scn8a, Cadm3, Dbn1, Ctnnd2, Jcad, ...
GO:0043197	dendritic spine	-1.48	0.00	Gria2, Kcnd3, Grin2a, Dlg4, Arhgap32, Mpp2, Nedd4, Atp1a2, Sez6, Atp1a3, Lrrc7, Ddn, Ntrk2, Dbn1, Apbb1, Fxr1, Arhgap33, Arhgap44, Shank3, Camk2a, Grin1, Kcnc3, Prrt2, Rph3a, Hpca, ...
GO:0008021	synaptic vesicle	-1.47	0.00	Rab3c, Kif1b, Gria2, Grin2a, Dlg4, Sv2b, Snap91, Trim9, Kif3c, Brsk1, Svop, Slc6a17, Stx1a, Syn1, Phf24, Grin1, Prrt2, Sh3gl2, Rph3a, Slc17a7, Syt1, Syt7, Snap25, Septin2, Kif1a, ...
GO:0005615	extracellular space	1.60	0.01	Cst7, Spp1, Apoe, Axl, Fam20c, Csf1, Lpl, Cd63, Il1b, Lgals3, Cxcl16, Apoc1, Ctsb, Lgals3bp, Serpine1, B2m, Lyz2, Plau, Igf1, Ccl4, Npc2, Cxcl10, Cd14, Tpt1, Fabp5, ...
...	...	...	...	...

Gene Set Enrichment Analysis (GSEA) for Database "GO-CC"

mm39 (Mus musculus)

Genes

We identified **20 up-regulated** and **51 down-regulated** terms from GO-CC in group "Microglia_KIN" compared to group "Microglia_WT" (Figure 16). The most significantly up-regulated terms were **melanosome, pigment granule, extracellular space** . The most significantly down-regulated terms were **axon, synaptic membrane, distal axon** . The most significantly terms are listed in Table 15.

Neurons_KIN vs. Neurons_WT

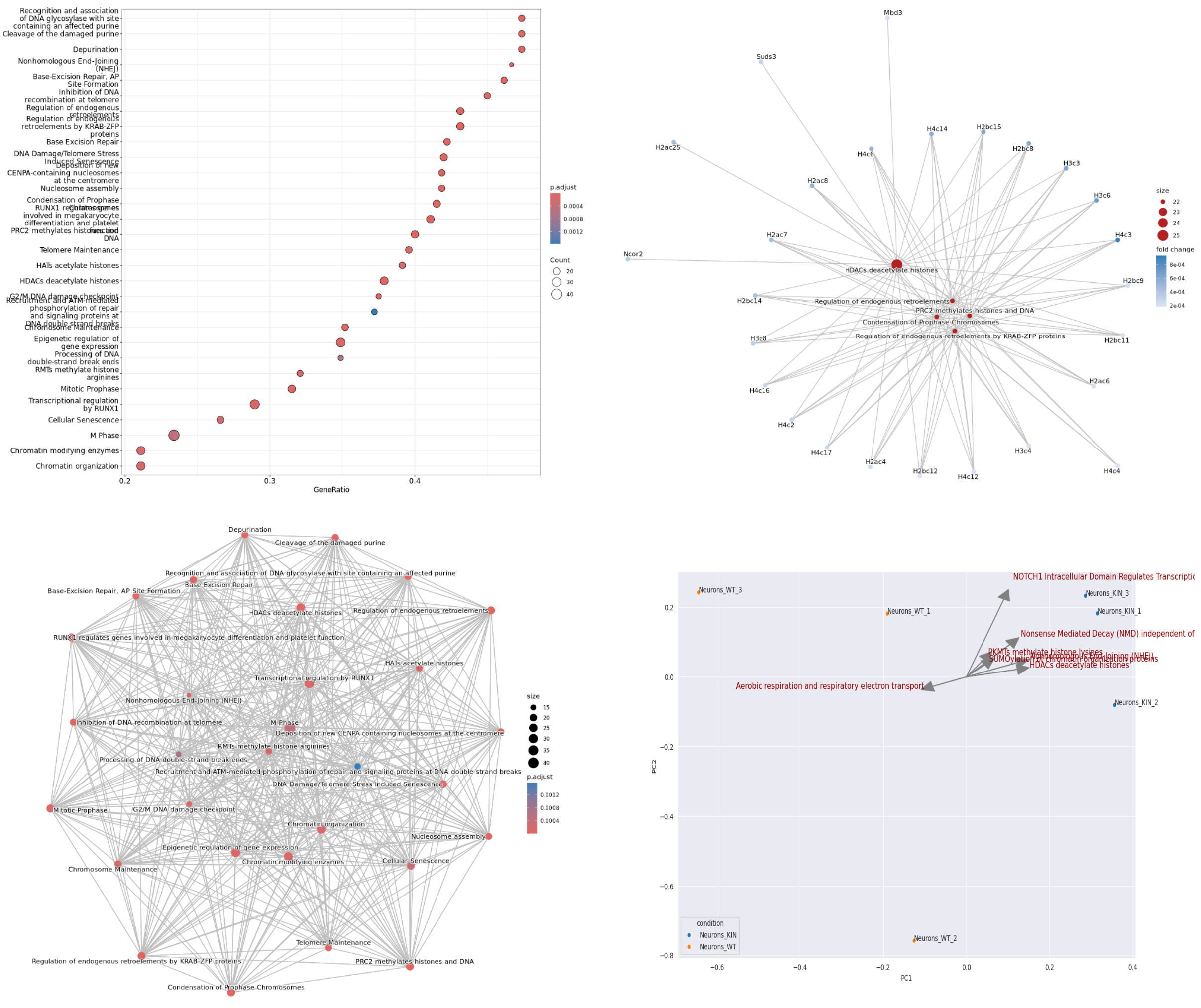

Figure 17. A) Dotplot of enriched pathways. Dots represent enriched pathways, with size indicating gene count and color showing significance (e.g., adjusted p-value). **B)** Cnetplot of gene-pathway relationships. Pathways and their associated genes are connected, with node size and edge thickness indicating enrichment and shared genes. **C)** Enrichment map plot showing overlapping gene sets making it easier to identify functional modules. **D)** PCA biplot showing the average loading of high-variance genes of up to 10 functional modules amongst the enriched terms.

Table 16. Top 15 enriched terms from Reactome in Neurons_KIN compared to Neurons_WT. based on gene expression.

ID	Term	NES	Adj. P-Value	Genes
R-MMU-3214815	HDACs deacetylate histones	2.50	0.00	H4c3, H3c6, H3c3, H2bc8, H2bc15, H4c14, H4c6, H2ac8, H2ac7, H2ac25, H2bc14, Ncor2, H3c8, Suds3, H4c16, H4c2, H4c17, H2ac4, H2ac6, H4c12, H3c4, H2bc9, H2bc11, Mbd3, H2bc12
R-MMU-9842860	Regulation of endogenous retroelements	2.44	0.00	H4c3, H3c6, H3c3, H2bc8, H2bc15, H4c14, H4c6, H2ac8, H2ac7, H2bc14, H3c8, H4c16, H4c2, H4c17, H2ac4, H2ac6, H4c12, H3c4, H2bc9, H2bc11, H2bc12, H4c4
R-MMU-9843940	Regulation of endogenous retroelements by KRAB-ZFP proteins	2.44	0.00	H4c3, H3c6, H3c3, H2bc8, H2bc15, H4c14, H4c6, H2ac8, H2ac7, H2bc14, H3c8, H4c16, H4c2, H4c17, H2ac4, H2ac6, H4c12, H3c4, H2bc9, H2bc11, H2bc12, H4c4
R-MMU-2299718	Condensation of Prophase Chromosomes	2.43	0.00	H4c3, H3c6, H3c3, H2bc8, H2bc15, H4c14, H4c6, H2ac8, H2ac7, H2bc14, H3c8, H4c16, H4c2, H4c17, H2ac4, H2ac6, H4c12, H3c4, H2bc9, H2bc11, H2bc12, H4c4
R-MMU-212300	PRC2 methylates histones and DNA	2.40	0.00	H4c3, H3c6, H3c3, H2bc8, H2bc15, H4c14, H4c6, H2ac8, H2ac7, H2bc14, H3c8, H4c16, H4c2, H4c17, H2ac4, H2ac6, H4c12, H3c4, H2bc9, H2bc11, H2bc12, H4c4
R-MMU-2559586	DNA Damage/Telomere Stress Induced Senescence	2.44	0.00	H4c3, H1f2, H2bc8, H2bc15, H4c14, H4c6, H2ac8, H2ac7, H2bc14, H4c16, H4c2, H1f4, H4c17, H2ac4, H2ac6, H4c12, H2bc9, H2bc11, H2bc12, H1f0, H4c4
R-MMU-110330	Recognition and association of DNA glycosylase with site containing an affected purine	2.42	0.00	H4c3, H2bc8, H2bc15, H4c14, H4c6, H2ac8, H2ac7, H2bc14, H4c16, H4c2, H4c17, H2ac4, H2ac6, H4c12, H2bc9, H2bc11, H2bc12, H4c4
R-MMU-110331	Cleavage of the damaged purine	2.42	0.00	H4c3, H2bc8, H2bc15, H4c14, H4c6, H2ac8, H2ac7, H2bc14, H4c16, H4c2, H4c17, H2ac4, H2ac6, H4c12, H2bc9, H2bc11, H2bc12, H4c4
R-MMU-73927	Depurination	2.42	0.00	H4c3, H2bc8, H2bc15, H4c14, H4c6, H2ac8, H2ac7, H2bc14, H4c16, H4c2, H4c17, H2ac4, H2ac6, H4c12, H2bc9, H2bc11, H2bc12, H4c4
R-MMU-73884	Base Excision Repair	2.39	0.00	H4c3, H2bc8, H2bc15, H4c14, H4c6, H2ac8, H2ac7, H2bc14, H4c16, H4c2, H4c17, H2ac4, Lig1, H2ac6, H4c12, H2bc9, H2bc11, H2bc12, H4c4
R-MMU-9670095	Inhibition of DNA recombination at telomere	2.42	0.00	H4c3, H2bc8, H2bc15, H4c14, H4c6, H2ac8, H2ac7, H2bc14, H4c16, H4c2, H4c17, H2ac4, H2ac6, H4c12, H2bc9, H2bc11, H2bc12, H4c4
R-MMU-8936459	RUNX1 regulates genes involved in megakaryocyte differentiation and platelet function	2.35	0.00	H4c3, H3c6, H3c3, H2bc8, H2bc15, H4c14, H4c6, H2ac8, H2ac7, H2bc14, H3c8, H4c16, H4c2, H4c17, H2ac4, H2ac6, H4c12, H3c4, H2bc9, H2bc11, H2bc12, H4c4, Ash2l
R-MMU-73929	Base-Excision Repair, AP Site Formation	2.41	0.00	H4c3, H2bc8, H2bc15, H4c14, H4c6, H2ac8, H2ac7, H2bc14, H4c16, H4c2, H4c17, H2ac4, H2ac6, H4c12, H2bc9, H2bc11, H2bc12, H4c4
R-MMU-606279	Deposition of new CENPA-containing nucleosomes at the centromere	2.37	0.00	H4c3, H2bc8, H2bc15, H4c14, H4c6, H2ac8, H2ac7, H2bc14, H4c16, H4c2, H4c17, H2ac4, H2ac6, H4c12, H2bc9, H2bc11, H2bc12, H4c4
R-MMU-774815	Nucleosome assembly	2.37	0.00	H4c3, H2bc8, H2bc15, H4c14, H4c6, H2ac8, H2ac7, H2bc14, H4c16, H4c2, H4c17, H2ac4, H2ac6, H4c12, H2bc9, H2bc11, H2bc12, H4c4
...	...	...	...	...

Gene Set Enrichment Analysis (GSEA) for Database "Reactome"

mm39 (Mus musculus)

Genes

We identified **63 up-regulated** and **2 down-regulated** terms from Reactome in group "Neurons_KIN" compared to group "Neurons_WT" (Figure 17). The most significantly up-regulated terms were **HDACs deacetylate histones, Regulation of endogenous retroelements, Regulation of endogenous retroelements by KRAB-ZFP proteins** . The most significantly down-regulated terms were **Aerobic respiration and respiratory electron transport, Respiratory electron transport** . The most significantly terms are listed in Table 16.

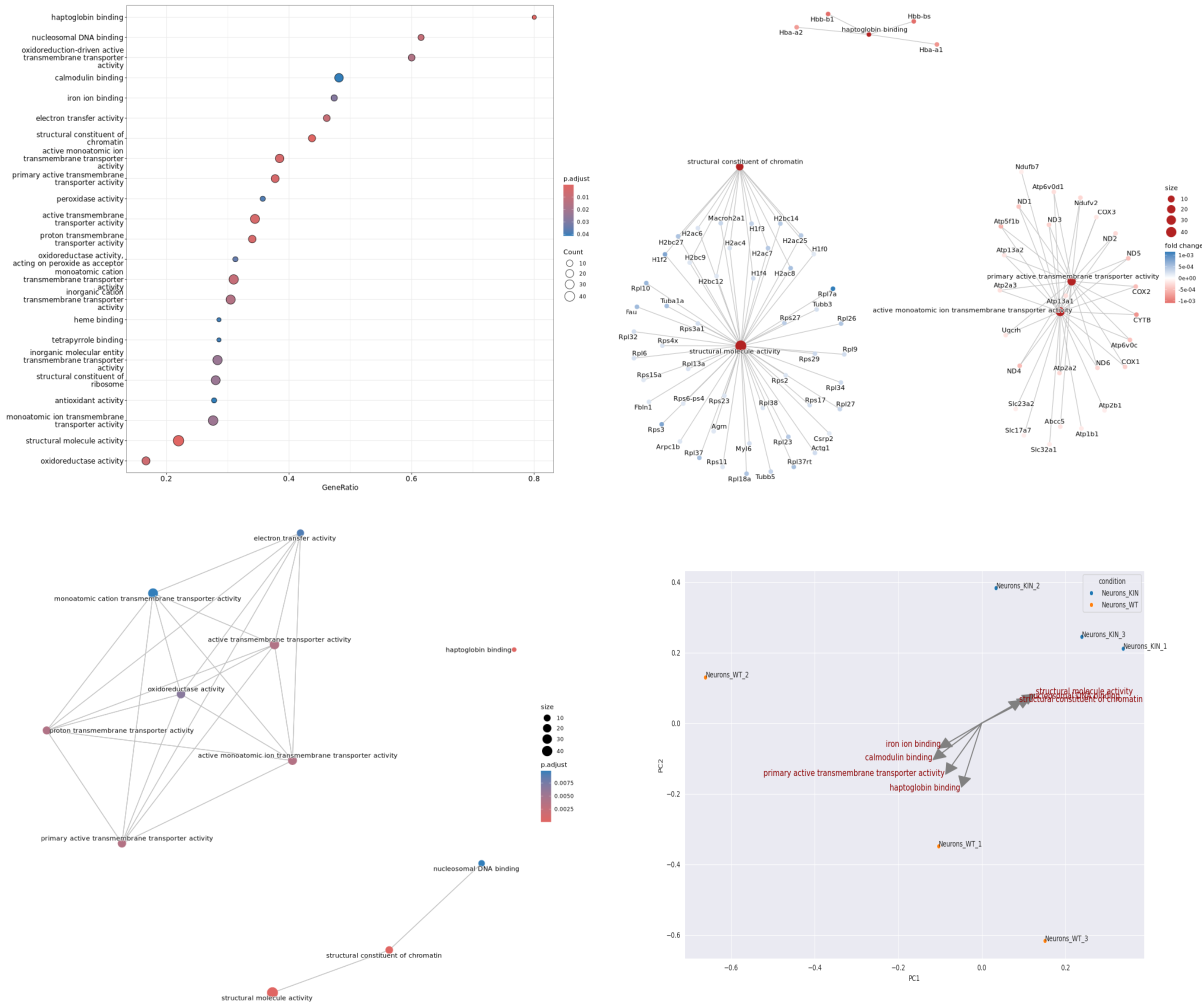

Figure 18. A) Dotplot of enriched pathways. Dots represent enriched pathways, with size indicating gene count and color showing significance (e.g., adjusted p-value). **B)** Cnetplot of gene-pathway relationships. Pathways and their associated genes are connected, with node size and edge thickness indicating enrichment and shared genes. **C)** Enrichment map plot showing overlapping gene sets making it easier to identify functional modules. **D)** PCA biplot showing the average loading of high-variance genes of up to 10 functional modules amongst the enriched terms.

Table 17. Top 15 enriched terms from GO-MF in Neurons_KIN compared to Neurons_WT. based on gene expression.

ID	Term	NES	Adj. P-Value	Genes
GO:0031720	haptoglobin binding	-1.93	0.00	Hba-a2, Hba-a1, Hbb-bs, Hbb-b1
GO:0005198	structural molecule activity	1.95	0.00	Rpl7a, Rps3, H1f2, Rpl10, Fau, Tuba1a, Rpl37rt, Rpl37, H2ac8, H2bc27, Rpl18a, Rpl26, H2ac7, Rpl23, H2ac25, H2bc14, Rps27, H1f3, Tubb5, Macroh2a1, Rpl27, Rps29, Rpl34, Actg1, Rpl9, ...
GO:0030527	structural constituent of chromatin	2.39	0.00	H1f2, H2ac8, H2bc27, H2ac7, H2ac25, H2bc14, H1f3, Macroh2a1, H1f4, H2ac4, H2ac6, H2bc9, H2bc12, H1f0
GO:0015399	primary active transmembrane transporter activity	-2.12	0.00	COX3, Abcc5, Atp13a2, Uqcrh, Atp2a3, Atp6v0d1, Atp13a1, ND2, Atp2a2, ND6, Ndufv2, COX1, ND3, ND1, Atp6v0c, ND5, COX2, ND4, Atp5f1b, CYTB
GO:0022853	active monoatomic ion transmembrane transporter activity	-2.06	0.00	Slc17a7, Slc23a2, Ndufb7, Atp2b1, Slc32a1, Atp1b1, COX3, Atp13a2, Uqcrh, Atp2a3, Atp6v0d1, Atp13a1, ND2, Atp2a2, ND6, Ndufv2, COX1, ND3, ND1, Atp6v0c, ND5, COX2, ND4, Atp5f1b, CYTB
GO:0015078	proton transmembrane transporter activity	-2.09	0.00	COX3, Uqcrh, Atp6v0d1, ND2, ND6, Ndufv2, Atp5pd, COX1, ND3, ATP6, ND1, Atp6v0c, ND5, COX2, ND4, Atp5f1b, CYTB
GO:0022804	active transmembrane transporter activity	-2.01	0.00	Slc17a7, Slc23a2, Ndufb7, Atp2b1, Slc29a4, Slc32a1, Atp1b1, Slc25a1, COX3, Abcc5, Atp13a2, Uqcrh, Xpr1, Atp2a3, Atp6v0d1, Atp13a1, Slc25a23, ND2, Atp2a2, ND6, Ndufv2, COX1, ND3, Mfsd12, ND1, ...
GO:0016491	oxidoreductase activity	-1.87	0.01	Fth1, Tecr, Hmgcr, ND2, ND6, Ndufv2, Ctbp1, Kdm4b, COX1, ND3, Scd2, ND1, ND5, COX2, ND4, Hbb-bt, Hba-a2, Hba-a1, CYTB, Hbb-bs, Hbb-b1
GO:0009055	electron transfer activity	-2.13	0.01	COX3, Uqcrh, ND2, ND6, Ndufv2, COX1, ND3, ND1, ND5, COX2, ND4, CYTB
GO:0031492	nucleosomal DNA binding	2.00	0.01	H1f10, H1f2, H1f3, Macroh2a1, Hmgn2, H1f4, H1f0, H2az1
GO:0008324	monoatomic cation transmembrane transporter activity	-1.84	0.01	Slc17a7, Slc23a2, Ndufb7, Atp2b1, Slc29a4, Hcn3, Cacna1c, Tmbim6, Ncs1, Slc32a1, Atp1b1, COX3, Atp13a2, Uqcrh, Kcnq2, Kcnc1, Atp2a3, Atp6v0d1, Atp13a1, ND2, Atp2a2, ND6, Ndufv2, Atp5pd, COX1, ...
GO:0022890	inorganic cation transmembrane transporter activity	-1.83	0.02	Slc17a7, Slc23a2, Ndufb7, Atp2b1, Hcn3, Cacna1c, Tmbim6, Ncs1, Slc32a1, Atp1b1, COX3, Uqcrh, Kcnq2, Kcnc1, Atp2a3, Atp6v0d1, ND2, Atp2a2, ND6, Ndufv2, Atp5pd, COX1, ND3, ATP6, ND1, ...
GO:0015453	oxidoreduction-driven active transmembrane transporter activity	-2.07	0.02	COX3, Uqcrh, ND2, ND6, Ndufv2, COX1, ND3, ND1, ND5, COX2, ND4, CYTB
GO:0015075	monoatomic ion transmembrane transporter activity	-1.73	0.02	Slc17a7, Slc23a2, Ndufb7, Atp2b1, Slc29a4, Hcn3, Cacna1c, Tmbim6, Ncs1, Slc32a1, Tmem120a, Atp1b1, COX3, Atp13a2, Uqcrh, Kcnq2, Sfxn3, Kcnc1, Atp2a3, Atp6v0d1, Atp13a1, ND2, Atp2a2, ND6, Ndufv2, ...
GO:0015318	inorganic molecular entity transmembrane transporter activity	-1.78	0.02	Slc17a7, Slc23a2, Ndufb7, Atp2b1, Hcn3, Cacna1c, Tmbim6, Ncs1, Slc32a1, Atp1b1, COX3, Uqcrh, Xpr1, Kcnq2, Kcnc1, Atp2a3, Atp6v0d1, Slc25a23, ND2, Atp2a2, ND6, Ndufv2, Atp5pd, COX1, ND3, ...
...	...	...	...	...

Gene Set Enrichment Analysis (GSEA) for Database "GO-MF"

mm39 (Mus musculus)

Genes

We identified **4 up-regulated** and **19 down-regulated** terms from GO-MF in group "Neurons_KIN" compared to group "Neurons_WT" (Figure 18). The most significantly up-regulated terms were **structural molecule activity, structural constituent of chromatin, nucleosomal DNA binding** . The most significantly down-regulated terms were **haptoglobin binding, primary active transmembrane transporter activity, active monoatomic ion transmembrane transporter activity** . The most significantly terms are listed in Table 17.

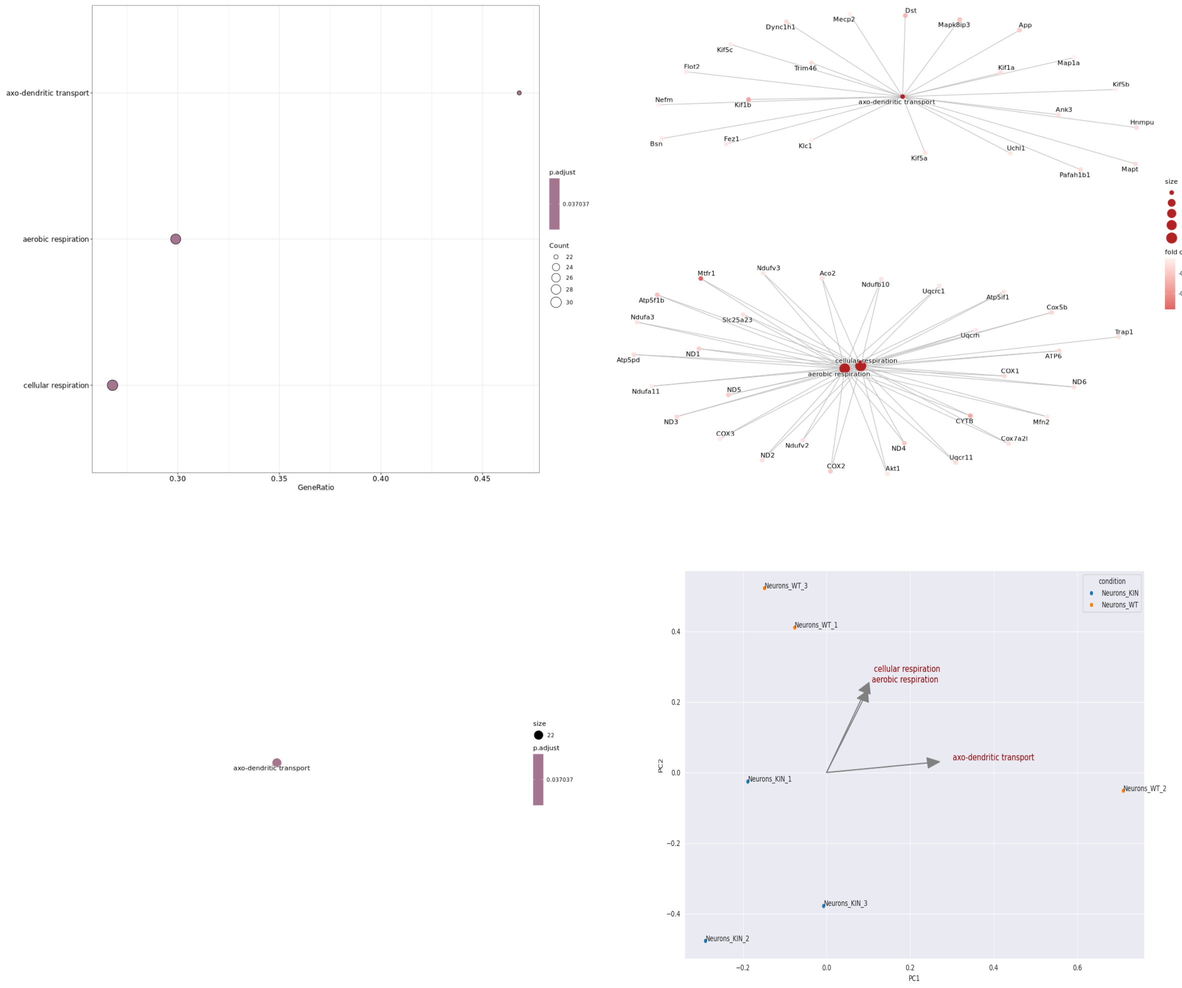

Figure 19. **A)** Dotplot of enriched pathways. Dots represent enriched pathways, with size indicating gene count and color showing significance (e.g., adjusted p-value). **B)** Cnetplot of gene-pathway relationships. Pathways and their associated genes are connected, with node size and edge thickness indicating enrichment and shared genes. **C)** Enrichment map plot showing overlapping gene sets making it easier to identify functional modules. **D)** PCA biplot showing the average loading of high-variance genes of up to 10 functional modules amongst the enriched terms.

Table 18. Top 3 enriched terms from GO-BP in Neurons_KIN compared to Neurons_WT. based on gene expression.

ID	Term	NES	Adj. P-Value	Genes
GO:0008088	axo-dendritic transport	-2.06	0.04	Kif5b, Klc1, Map1a, Nefm, Mecp2, Kif5c, Flot2, Bsn, Fez1, Uchl1, Kif5a, Ank3, Kif1a, Dync1h1, Trim46, Mapt, Pafah1b1, Hnrnpu, App, Mapk8ip3, Dst, Kif1b
GO:0009060	aerobic respiration	-2.02	0.04	COX3, Mfn2, Ndufa11, Akt1, Uqcrh, Uqcrc1, Ndufv3, Ndufa3, Ndufb10, Cox7a2l, Atp5if1, Slc25a23, Aco2, ND2, ND6, Uqcr11, Ndufv2, Atp5pd, COX1, ND3, ATP6, Cox5b, ND1, ND5, COX2, ...
GO:0045333	cellular respiration	-1.97	0.04	COX3, Mfn2, Ndufa11, Akt1, Uqcrh, Uqcrc1, Ndufv3, Ndufa3, Ndufb10, Cox7a2l, Atp5if1, Slc25a23, Aco2, ND2, Trap1, ND6, Uqcr11, Ndufv2, Atp5pd, COX1, ND3, ATP6, Cox5b, ND1, ND5, ...

Gene Set Enrichment Analysis (GSEA) for Database "GO-BP"

mm39 (Mus musculus)

Genes

We identified **0 up-regulated** and **3 down-regulated** terms from GO-BP in group "Neurons_KIN" compared to group "Neurons_WT" (Figure 19). The most significantly down-regulated terms were **axo-dendritic transport, aerobic respiration, cellular respiration** . The most significantly terms are listed in Table 18.

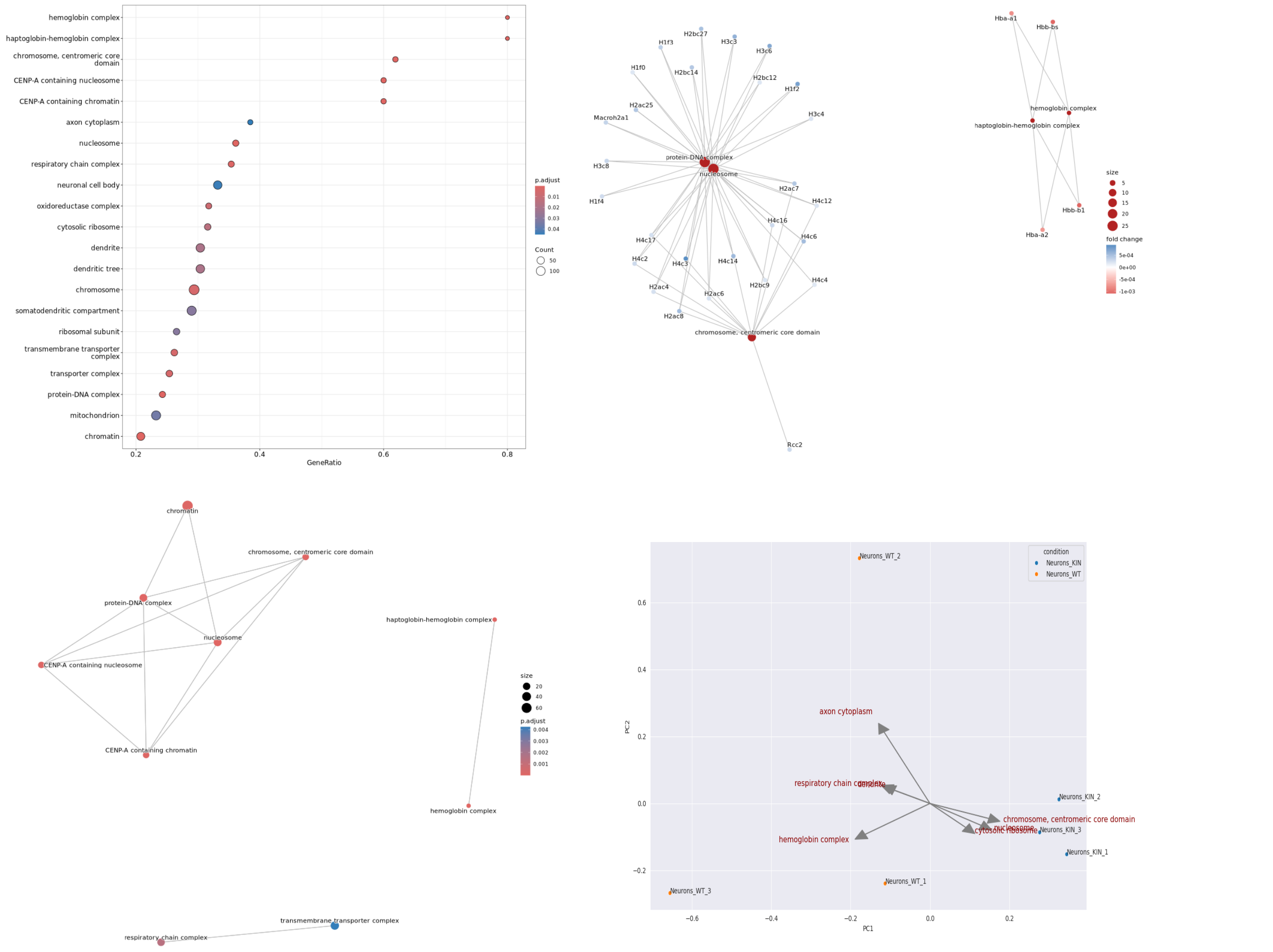

Figure 20. **A)** Dotplot of enriched pathways. Dots represent enriched pathways, with size indicating gene count and color showing significance (e.g., adjusted p-value). **B)** Cnetplot of gene-pathway relationships. Pathways and their associated genes are connected, with node size and edge thickness indicating enrichment and shared genes. **C)** Enrichment map plot showing overlapping gene sets making it easier to identify functional modules. **D)** PCA biplot showing the average loading of high-variance genes of up to 10 functional modules amongst the enriched terms.

Table 19. Top 15 enriched terms from GO-CC in Neurons_KIN compared to Neurons_WT. based on gene expression.

ID	Term	NES	Adj. P-Value	Genes
GO:0000786	nucleosome	2.50	0.00	H4c3, H1f2, H3c6, H3c3, H4c14, H4c6, H2ac8, H2bc27, H2ac7, H2ac25, H2bc14, H1f3, Macroh2a1, H3c8, H4c16, H4c2, H1f4, H4c17, H2ac4, H2ac6, H4c12, H3c4, H2bc9, H2bc12, H1f0, ...
GO:0005833	hemoglobin complex	-1.86	0.00	Hba-a2, Hba-a1, Hbb-bs, Hbb-b1
GO:0031838	haptoglobin-hemoglobin complex	-1.86	0.00	Hba-a2, Hba-a1, Hbb-bs, Hbb-b1
GO:0034506	chromosome, centromeric core domain	2.38	0.00	H4c3, H4c14, H4c6, H2ac8, H2ac7, H4c16, H4c2, Rcc2, H4c17, H2ac4, H2ac6, H4c12, H4c4
GO:0032993	protein-DNA complex	2.17	0.00	H4c3, H1f2, H3c6, H3c3, H4c14, H4c6, H2ac8, H2bc27, H2ac7, H2ac25, H2bc14, H1f3, Macroh2a1, H3c8, H4c16, H4c2, H1f4, H4c17, H2ac4, H2ac6, H4c12, H3c4, H2bc9, H2bc12, H1f0
GO:0000785	chromatin	1.71	0.00	H4c3, H1f2, Tcf4, H3c6, H3c3, H4c14, H4c6, H2ac8, H2bc27, H2ac7, H2ac25, H2bc14, H1f3, Ncor2, Macroh2a1, Mad2l2, Egr1, H3c8, Suds3, H4c16, Hdac5, H4c2, Actg1, Hmgn2, H1f4, ...
GO:0043505	CENP-A containing nucleosome	2.34	0.00	H4c3, H4c14, H4c6, H2ac8, H2ac7, H4c16, H4c2, H4c17, H2ac4, H2ac6, H4c12, H4c4
GO:0061638	CENP-A containing chromatin	2.34	0.00	H4c3, H4c14, H4c6, H2ac8, H2ac7, H4c16, H4c2, H4c17, H2ac4, H2ac6, H4c12, H4c4
GO:0098803	respiratory chain complex	-2.07	0.00	COX3, Ndufa11, Uqcrh, Uqcrc1, Ndufv3, Ndufa3, Ndufb10, Cox7a2l, ND2, ND6, Uqcr11, Ndufv2, Atp5pd, COX1, ND3, ATP6, Cox5b, ND1, ND5, COX2, ND4, Atp5f1b, CYTB
GO:1902495	transmembrane transporter complex	-1.86	0.00	COX3, Ndufa11, Uqcrh, Kcnq2, Uqcrc1, Ndufv3, Akap9, Kcnc1, Ndufa3, Vamp2, Ndufb10, Atp6v0d1, Cox7a2l, ND2, Atp2a2, ND6, Uqcr11, Ndufv2, Atp5pd, COX1, ND3, ATP6, Akap6, Cox5b, Calm1, ...
GO:1990351	transporter complex	-1.85	0.00	COX3, Ndufa11, Uqcrh, Kcnq2, Uqcrc1, Ndufv3, Akap9, Kcnc1, Ndufa3, Vamp2, Ndufb10, Atp6v0d1, Cox7a2l, ND2, Atp2a2, ND6, Uqcr11, Ndufv2, Atp5pd, COX1, ND3, ATP6, Akap6, Cox5b, Calm1, ...
GO:0005694	chromosome	1.49	0.00	H4c3, H1f10, H1f2, Tcf4, H3c6, H3c3, H4c14, Tuba1a, H4c6, H2ac8, H2bc27, H2ac7, H2ac25, H2bc14, H1f3, Ncor2, Macroh2a1, Mad2l2, Egr1, H3c8, Suds3, H4c16, Hdac5, H4c2, Rcc2, ...
GO:1990204	oxidoreductase complex	-1.96	0.01	COX3, Ndufa11, Uqcrh, Uqcrc1, Ndufv3, Ndufa3, Ndufb10, Cox7a2l, ND2, ND6, Uqcr11, Ndufv2, COX1, ND3, Cox5b, ND1, ND5, COX2, ND4, CYTB
GO:0022626	cytosolic ribosome	1.77	0.01	Rpl7a, Rps3, Rpl10, Fau, Rpl37rt, Rpl37, Rpl18a, Rpl26, Rpl23, Rps27, Rpl27, Rps29, Rpl34, Rpl9, Rps3a1, Rpl32, Rps17, Rpl6, Rpl13a, Rps4x, Rpl38, Rps2, Rps23, Rps11, Rps6-ps4, ...
GO:0030425	dendrite	-1.56	0.02	Ptch1, Cd3e, Apc, Stau1, Dlg3, Whrn, Thy1, Atxn10, Dip2b, Srgap2, Ntrk2, Ppt1, Git1, Bptf, Arhgap33, Lrrc7, Srsf10, Map1a, Gripap1, Src, Amigo1, Txn2, Mt3, Wdr47, Atp2b1, ...
...	...	...	...	...

Gene Set Enrichment Analysis (GSEA) for Database "GO-CC"

mm39 (Mus musculus)

Genes

We identified **9 up-regulated** and **12 down-regulated** terms from GO-CC in group "Neurons_KIN" compared to group "Neurons_WT" (Figure 20). The most significantly up-regulated terms were **nucleosome, chromosome, centromeric core domain, protein-DNA complex** . The most significantly down-regulated terms were **hemoglobin complex, haptoglobin-hemoglobin complex, respiratory chain complex** . The most significantly terms are listed in Table 19.

References

1. Martin, M. (2011) Cutadapt Removes Adapter Sequences from High-Throughput Sequencing Reads. EMBnet Journal, 17, 10-12. https://doi.org/10.14806/ej.17.1.200
2. https://www.bioinformatics.babraham.ac.uk/projects/fastqc/
3. Langmead, B., & Salzberg, S. L. (2012). Fast gapped-read alignment with Bowtie 2. Nature methods, 9(4), 357-359. https://doi.org/10.1038/nmeth.1923
4. Pantaleoni J, Subtil N. NVBIO. 2015. https://nvlabs.github.io/nvbio
5. Love, M. I., Huber, W., & Anders, S. (2014). Moderated estimation of fold change and dispersion for RNA-seq data with DESeq2. Genome biology, 15(12), 550. https://doi.org/10.1186/s13059-014-0550-8
6. Anders, S., Pyl, P. T., & Huber, W. (2015). HTSeq--a Python framework to work with high-throughput sequencing data. Bioinformatics (Oxford, England), 31(2), 166-169. https://doi.org/10.1093/bioinformatics/btu638
7. Stephens, M. (2017). False discovery rates: a new deal. Biostatistics, 18(2), 275-294.
8. Xu, S., Hu, E., Cai, Y., Xie, Z., Luo, X., Zhan, L., ... & Yu, G. (2024). Using clusterProfiler to characterize multiomics data. Nature protocols, 1-29.
9. https://www.canvasxpress.org/
10. Ewels, P., Magnusson, M., Lundin, S., & Käller, M. (2016). MultiQC: summarize analysis results for multiple tools and samples in a single report. Bioinformatics (Oxford, England), 32(19), 3047-3048. https://doi.org/10.1093/bioinformatics/btw354

Suppl. Material S2

Materials and Methods

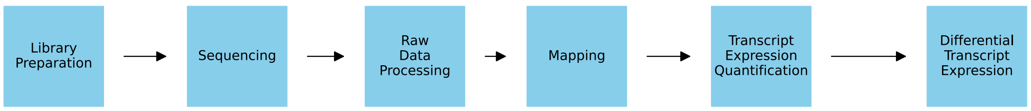

Figure 1: Directed graph showing the order of operations.

Library Preparation and Sequencing

Small RNA libraries were prepared using the efficient smallRNA-Seq Kit (GenXPro GmbH) according to the manual of the manufacturer. The smallRNA molecules were ligated with the 3'- and 5'-adapters containing TrueQuant UMIs, reverse transcribed, followed by PCR amplification with a minimal number of cycles and silica-bead purification. Sequencing was performed on an Illumina NEXTSEQ instrument with 1x76 bps.

Data Processing

Unprocessed sequencing reads were adapter-trimmed and quality-trimmed using Cutadapt (version 4.6, [1]) with the arguments "-e 0.1 -O 3 -q 20 -m 20 -n 8". FastQC (0.11.9, [2]) was used to assess the quality of sequencing reads. Processed sequencing reads were mapped using Bowtie2 (2.4.4, [3]) on mirna (with arguments: "--local --ma 1 --score-min L,0,0.9 --mp 1,1 --rdg 2,1 --rfg 2,1") and trna (with arguments: "--sensitive --local") and pirna (with arguments: "--sensitive --local") and ENSEMBL_ncrna (with arguments: "--sensitive --local") and ENSEMBL_cdna (with arguments: "--sensitive --local") of mm39 (Mus musculus). The mapping was done iteratively, meaning only those reads not mapping on the previous reference were mapped on the next one. Quantification of mapped reads to each transcript was performed using HTSeq (version 2.0.2, [4]) mirna (with arguments: "-i transcript_id -r name -a 0 -m union" and strandedness "no") and trna (with arguments: "-i transcript_id -r name -a 0 -m union" and strandedness "no") and pirna (with arguments: "-i transcript_id -r name -a 0 -m union" and strandedness "no") and ENSEMBL_ncrna (with arguments: "-i transcript_id -r name -a 0 -m union" and strandedness "no") and ENSEMBL_cdna (with arguments: "-i transcript_id -r name -a 0 -m union" and strandedness "no"). MultiQC (version 1.23, [7]) was used to create a single report visualising output from multiple tools across many samples, enabling global trends and biases to be quickly identified.

Differential Expression Analysis (DEA)

DEA was performed using DESeq2 (version 1.38, [5]). Only entries having at least a raw count of [None, None, None, None, None] in at least [None, None, None, None, None] samples were used in the DEA. Log2FoldChange values were shrinked using "ashr" ([6]). DEA results with an FDR-adjusted p-value lesser or equal than [None, None, None, None, None] and an absolute log2FoldChange greater or equal than [None, None, None, None, None] were called significant.

Results

Quality Control

Sample	# Total reads
Astrocytes_KIN_1	6,132,120
Astrocytes_KIN_2	6,556,783
Astrocytes_KIN_3	6,287,151
Astrocytes_WT_1	5,657,484
Astrocytes_WT_2	6,477,263
Astrocytes_WT_3	6,058,159
Microglia_KIN_1	6,297,143
Microglia_KIN_2	6,299,618
Microglia_KIN_3	6,562,774
Microglia_WT_1	6,331,412
Microglia_WT_2	6,689,722
Microglia_WT_3	6,885,245
Neurons_KIN_1	6,680,289
Neurons_KIN_2	7,139,079
Neurons_KIN_3	6,885,199
Neurons_WT_1	7,317,238
Neurons_WT_2	7,433,159
Neurons_WT_3	7,441,052

Table 1: Reads after raw data processing for nan for nan

Sample	Mirna	Trna	Pirna	ENSEMBL_ncrna	ENSEMBL_cdna
Astrocytes_KIN_1	10.14 %	11.63 %	4.33 %	54.44 %	17.46 %
Astrocytes_KIN_2	9.02 %	12.29 %	4.34 %	55.62 %	17.57 %
Astrocytes_KIN_3	8.48 %	12.2 %	4.5 %	57.4 %	18.05 %
Astrocytes_WT_1	4.76 %	10.61 %	4.75 %	56.05 %	18.15 %
Astrocytes_WT_2	8.32 %	11.13 %	4.86 %	58.11 %	17.78 %
Astrocytes_WT_3	5.74 %	9.62 %	4.61 %	56.37 %	17.95 %
Microglia_KIN_1	5.68 %	13.58 %	4.96 %	53.04 %	19.65 %
Microglia_KIN_2	9.05 %	16.8 %	6.64 %	53.85 %	20.67 %
Microglia_KIN_3	8.79 %	17.43 %	6.52 %	52.26 %	20.12 %
Microglia_WT_1	5.02 %	9.09 %	6.64 %	55.4 %	20.87 %
Microglia_WT_2	5.05 %	11.24 %	5.92 %	56.16 %	20.93 %
Microglia_WT_3	5.82 %	8.28 %	6.63 %	55.61 %	20.76 %
Neurons_KIN_1	1.79 %	4.94 %	4.97 %	54.18 %	18.11 %
Neurons_KIN_2	1.15 %	4.37 %	4.85 %	57.04 %	19.02 %
Neurons_KIN_3	1.37 %	3.87 %	4.62 %	56.63 %	18.61 %
Neurons_WT_1	0.71 %	2.15 %	3.6 %	50.54 %	16.17 %
Neurons_WT_2	1.55 %	8.5 %	4.18 %	49.89 %	16.25 %
Neurons_WT_3	0.78 %	3.27 %	4.12 %	47.85 %	16.05 %

Table 2: Percentage of reads mapping to each reference.

Sample	Condition
Astrocytes_KIN_1	Astrocytes_KIN
Astrocytes_KIN_2	Astrocytes_KIN
Astrocytes_KIN_3	Astrocytes_KIN
Astrocytes_WT_1	Astrocytes_WT
Astrocytes_WT_2	Astrocytes_WT
Astrocytes_WT_3	Astrocytes_WT
Microglia_KIN_1	Microglia_KIN
Microglia_KIN_2	Microglia_KIN
Microglia_KIN_3	Microglia_KIN
Microglia_WT_1	Microglia_WT
Microglia_WT_2	Microglia_WT
Microglia_WT_3	Microglia_WT
Neurons_KIN_1	Neurons_KIN
Neurons_KIN_2	Neurons_KIN
Neurons_KIN_3	Neurons_KIN
Neurons_WT_1	Neurons_WT
Neurons_WT_2	Neurons_WT
Neurons_WT_3	Neurons_WT

Table 3: Sample Metadata.

We studied 18 samples in total of which 18 were analyzed using tRNA-Seq. The samples were grouped by condition (Astrocytes_KIN, Astrocytes_WT, Microglia_KIN, Microglia_WT, Neurons_KIN, Neurons_WT). After raw data processing the average sequencing depth per sample was 3,251,031 reads. On average, 5.18% of reads mapped to mirna and 9.5% of reads mapped to trna and 5.06% of reads mapped to pirna and 54.47% of reads mapped to ENSEMBL_ncrna and 18.56% of reads mapped to ENSEMBL_cdna.

General Results

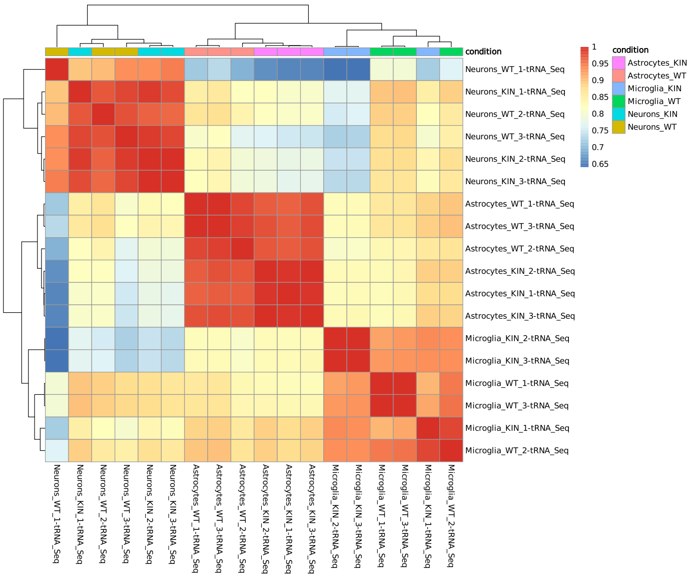

Figure 2: Heatmap showing correlation of samples based on transcript expression.

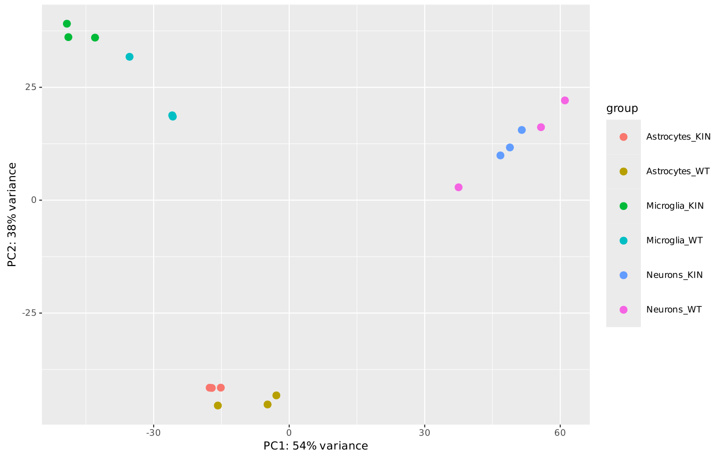

Figure 3: Principal Component Analysis (PCA) based on the 500 transcripts with the highest variance.

Results for Astrocytes_KIN

Name	Description
Yipf1-208	Yip1 domain family, member 1 [Source:MGI Symbol;Acc:MGI:1915532]
Hspa8-208	heat shock protein 8 [Source:MGI Symbol;Acc:MGI:105384]
mmu-miR-125b-5p	length=22;sequence=TCCCTGAGACCCTAACTTGTGA
mt-Ts2-201	mitochondrially encoded tRNA serine 2 [Source:MGI Symbol;Acc:MGI:102474]
Rpl13-202	ribosomal protein L13 [Source:MGI Symbol;Acc:MGI:105922]
Snord68-201	small nucleolar RNA, C/D box 68 [Source:MGI Symbol;Acc:MGI:3819550]
Snord2-201	small nucleolar RNA, C/D box 2 [Source:MGI Symbol;Acc:MGI:3819528]
mmu-miR-9-5p	length=23;sequence=TCTTTGGTTATCTAGCTGTATGA
Ttyh1-210	tweety family member 1 [Source:MGI Symbol;Acc:MGI:1889007]
mmu-piR-68681	length=32
mmu-piR-22375	length=29;sequence=CTGAAATGAAGAGAATACTCTTGCTGATC
Snord83b-201	small nucleolar RNA, C/D box 83B [Source:MGI Symbol;Acc:MGI:3819557]
mt-Tv-201	mitochondrially encoded tRNA valine [Source:MGI Symbol;Acc:MGI:102472]
Snord14c-201	small nucleolar RNA, C/D box 14C [Source:MGI Symbol;Acc:MGI:97971]
Gm23344-201	predicted gene, 23344 [Source:MGI Symbol;Acc:MGI:5453121]

Table 4: Top 15 expressed transcripts and smallRNAs based on tags-per-million-normalized counts.

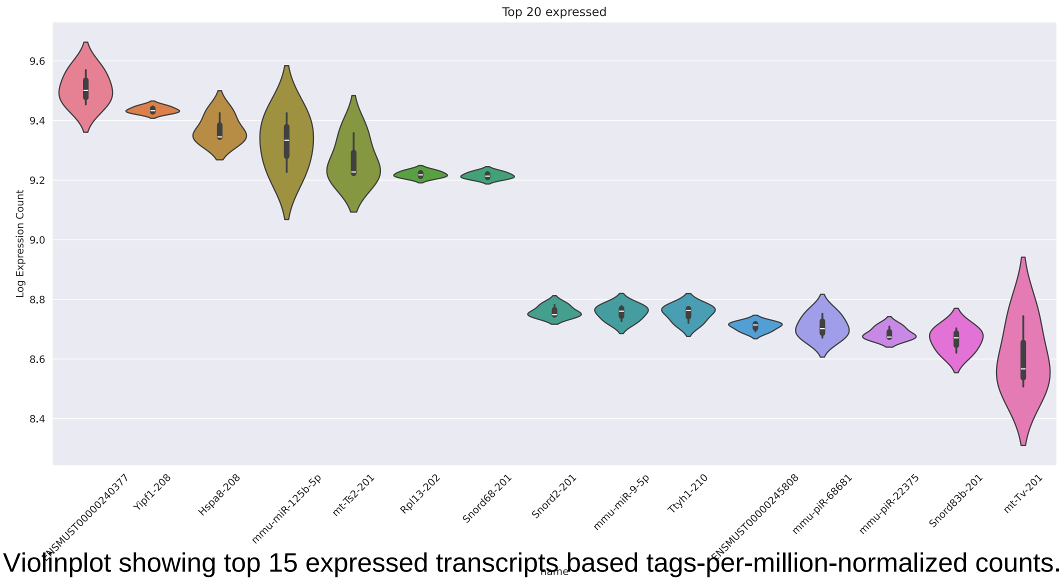

Figure 4: Violinplot showing top 15 expressed transcripts based tags-per-million-normalized counts.

Results for Astrocytes_WT

Name	Description
mt-Ts2-201	mitochondrially encoded tRNA serine 2 [Source:MGI Symbol;Acc:MGI:102474]
Hspa8-208	heat shock protein 8 [Source:MGI Symbol;Acc:MGI:105384]
Rpl13-202	ribosomal protein L13 [Source:MGI Symbol;Acc:MGI:105922]
Snord68-201	small nucleolar RNA, C/D box 68 [Source:MGI Symbol;Acc:MGI:3819550]
Yipf1-208	Yip1 domain family, member 1 [Source:MGI Symbol;Acc:MGI:1915532]
mmu-piR-68681	length=32
mt-Tv-201	mitochondrially encoded tRNA valine [Source:MGI Symbol;Acc:MGI:102472]
Snord83b-201	small nucleolar RNA, C/D box 83B [Source:MGI Symbol;Acc:MGI:3819557]
mmu-miR-125b-5p	length=22;sequence=TCCCTGAGACCCTAACTTGTGA
Snord61-201	small nucleolar RNA, C/D box 61 [Source:MGI Symbol;Acc:MGI:2148805]
Gm23344-201	predicted gene, 23344 [Source:MGI Symbol;Acc:MGI:5453121]
mmu-miR-9-5p	length=23;sequence=TCTTTGGTTATCTAGCTGTATGA
Snord2-201	small nucleolar RNA, C/D box 2 [Source:MGI Symbol;Acc:MGI:3819528]
mmu-piR-50001	length=30;sequence=TTCGCCTTTTACTAAAGATTTCCGTGGAGA
Snord14c-201	small nucleolar RNA, C/D box 14C [Source:MGI Symbol;Acc:MGI:97971]

Table 5: Top 15 expressed transcripts and smallRNAs based on tags-per-million-normalized counts.

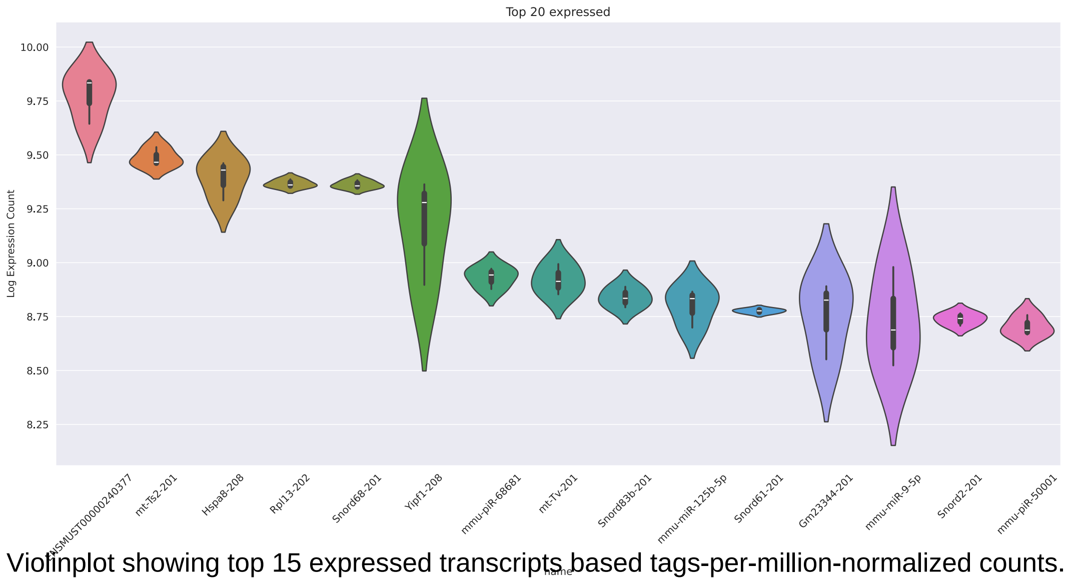

Figure 5: Violinplot showing top 15 expressed transcripts based tags-per-million-normalized counts.

Results for Microglia_KIN

Name	Description
Yipf1-208	Yip1 domain family, member 1 [Source:MGI Symbol;Acc:MGI:1915532]
mmu-piR-68681	length=32
mmu-miR-146a-5p	length=22;sequence=TGAGAACTGAATTCCATGGGTT
Rpl13-202	ribosomal protein L13 [Source:MGI Symbol;Acc:MGI:105922]
Snord68-201	small nucleolar RNA, C/D box 68 [Source:MGI Symbol;Acc:MGI:3819550]
Snord43-201	small nucleolar RNA, C/D box 43 [Source:MGI Symbol;Acc:MGI:3819534]
Snord2-201	small nucleolar RNA, C/D box 2 [Source:MGI Symbol;Acc:MGI:3819528]
Hspa8-208	heat shock protein 8 [Source:MGI Symbol;Acc:MGI:105384]
Snord83b-201	small nucleolar RNA, C/D box 83B [Source:MGI Symbol;Acc:MGI:3819557]
mmu-piR-22375	length=29;sequence=CTGAAATGAAGAGAATACTCTTGCTGATC
Mettl26-201	methyltransferase like 26 [Source:MGI Symbol;Acc:MGI:1915597]
Mir146-201	microRNA 146 [Source:MGI Symbol;Acc:MGI:2676831]
mmu-piR-23501	length=32
mmu-piR-69027	length=28;sequence=AAAGTCAGCCCTCGACACAAGGGTTTGT
Rpl7a-202	ribosomal protein L7A [Source:MGI Symbol;Acc:MGI:1353472]

Table 6: Top 15 expressed transcripts and smallRNAs based on tags-per-million-normalized counts.

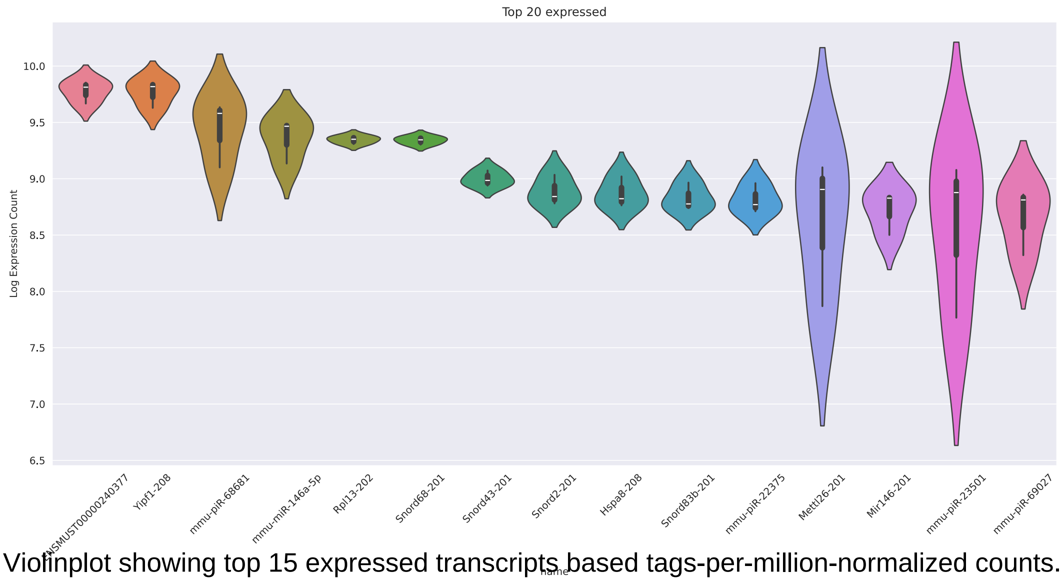

Figure 6: Violinplot showing top 15 expressed transcripts based tags-per-million-normalized counts.

Results for Microglia_WT

Name	Description
Rpl13-202	ribosomal protein L13 [Source:MGI Symbol;Acc:MGI:105922]
Snord68-201	small nucleolar RNA, C/D box 68 [Source:MGI Symbol;Acc:MGI:3819550]
mmu-piR-68681	length=32
Yipf1-208	Yip1 domain family, member 1 [Source:MGI Symbol;Acc:MGI:1915532]
Hspa8-208	heat shock protein 8 [Source:MGI Symbol;Acc:MGI:105384]
Snord43-201	small nucleolar RNA, C/D box 43 [Source:MGI Symbol;Acc:MGI:3819534]
Snord83b-201	small nucleolar RNA, C/D box 83B [Source:MGI Symbol;Acc:MGI:3819557]
Snord2-201	small nucleolar RNA, C/D box 2 [Source:MGI Symbol;Acc:MGI:3819528]
mmu-piR-50001	length=30;sequence=TTCGCCTTTTACTAAAGATTTCCGTGGAGA
mmu-piR-22375	length=29;sequence=CTGAAATGAAGAGAATACTCTTGCTGATC
mmu-piR-69027	length=28;sequence=AAAGTCAGCCCTCGACACAAGGGTTTGT
Rpl7a-202	ribosomal protein L7A [Source:MGI Symbol;Acc:MGI:1353472]
Creb5-215	cAMP responsive element binding protein 5 [Source:MGI Symbol;Acc:MGI:2443973]
mmu-miR-146a-5p	length=22;sequence=TGAGAACTGAATTCCATGGGTT
Snord55-201	small nucleolar RNA, C/D box 55 [Source:MGI Symbol;Acc:MGI:3819543]

Table 7: Top 15 expressed transcripts and smallRNAs based on tags-per-million-normalized counts.

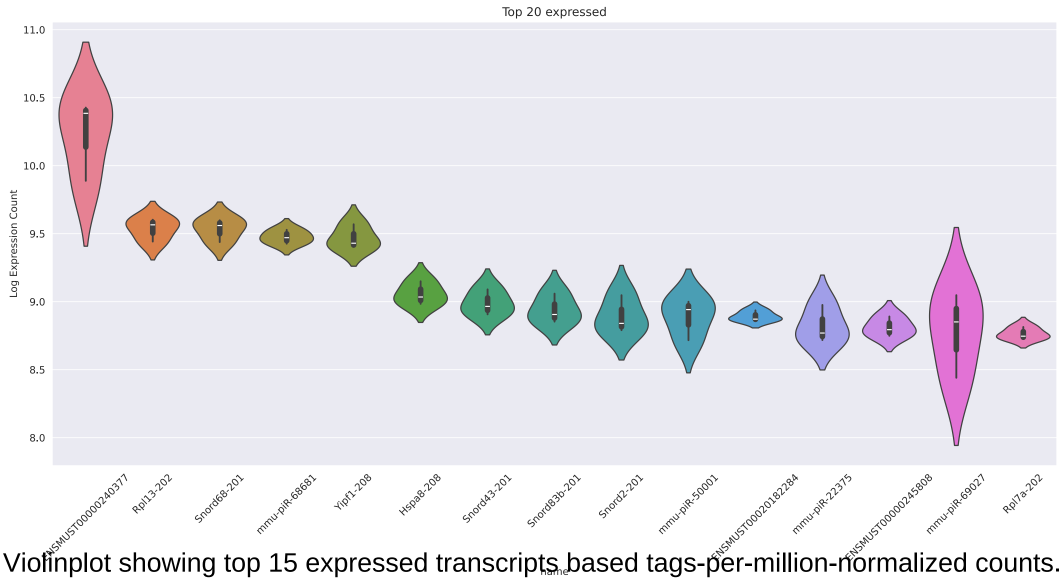

Figure 7: Violinplot showing top 15 expressed transcripts based tags-per-million-normalized counts.

Results for Neurons_KIN

Name	Description
Rpl13-202	ribosomal protein L13 [Source:MGI Symbol;Acc:MGI:105922]
Snord68-201	small nucleolar RNA, C/D box 68 [Source:MGI Symbol;Acc:MGI:3819550]
Hspa8-208	heat shock protein 8 [Source:MGI Symbol;Acc:MGI:105384]
Snord32a-201	small nucleolar RNA, C/D box 32A [Source:MGI Symbol;Acc:MGI:1351324]
Nop56-212	NOP56 ribonucleoprotein [Source:MGI Symbol;Acc:MGI:1914384]
Snord96a-201	small nucleolar RNA, C/D box 96A [Source:MGI Symbol;Acc:MGI:3819568]
Gm23344-201	predicted gene, 23344 [Source:MGI Symbol;Acc:MGI:5453121]
Gm26202-201	predicted gene, 26202 [Source:MGI Symbol;Acc:MGI:5455979]
Snord57-201	small nucleolar RNA, C/D box 57 [Source:MGI Symbol;Acc:MGI:3819544]
Snord83b-201	small nucleolar RNA, C/D box 83B [Source:MGI Symbol;Acc:MGI:3819557]
Rack1-206	receptor for activated C kinase 1 [Source:MGI Symbol;Acc:MGI:101849]
mmu-piR-58792	length=29;sequence=TGGAAAGGATGAACGAACTTGGCCTGACC
Snord2-201	small nucleolar RNA, C/D box 2 [Source:MGI Symbol;Acc:MGI:3819528]
Rpl7a-202	ribosomal protein L7A [Source:MGI Symbol;Acc:MGI:1353472]
mmu-piR-22375	length=29;sequence=CTGAAATGAAGAGAATACTCTTGCTGATC

Table 8: Top 15 expressed transcripts and smallRNAs based on tags-per-million-normalized counts.

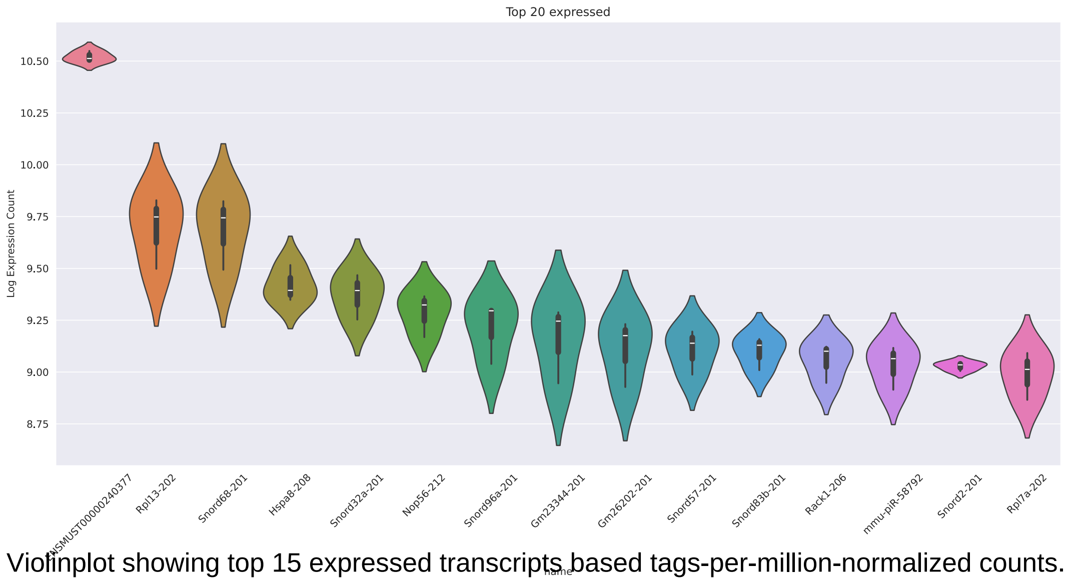

Figure 8: Violinplot showing top 15 expressed transcripts based tags-per-million-normalized counts.

Results for Neurons_WT

Name	Description
Rpl13-202	ribosomal protein L13 [Source:MGI Symbol;Acc:MGI:105922]
Snord68-201	small nucleolar RNA, C/D box 68 [Source:MGI Symbol;Acc:MGI:3819550]
Snord32a-201	small nucleolar RNA, C/D box 32A [Source:MGI Symbol;Acc:MGI:1351324]
Gm23344-201	predicted gene, 23344 [Source:MGI Symbol;Acc:MGI:5453121]
Nop56-212	NOP56 ribonucleoprotein [Source:MGI Symbol;Acc:MGI:1914384]
Hspa8-208	heat shock protein 8 [Source:MGI Symbol;Acc:MGI:105384]
Snord57-201	small nucleolar RNA, C/D box 57 [Source:MGI Symbol;Acc:MGI:3819544]
Snord55-201	small nucleolar RNA, C/D box 55 [Source:MGI Symbol;Acc:MGI:3819543]
Snord96a-201	small nucleolar RNA, C/D box 96A [Source:MGI Symbol;Acc:MGI:3819568]
Snord83b-201	small nucleolar RNA, C/D box 83B [Source:MGI Symbol;Acc:MGI:3819557]
Gm26202-201	predicted gene, 26202 [Source:MGI Symbol;Acc:MGI:5455979]
mmu-piR-58792	length=29;sequence=TGGAAAGGATGAACGAACTTGGCCTGACC
Rpl7a-202	ribosomal protein L7A [Source:MGI Symbol;Acc:MGI:1353472]
Rack1-206	receptor for activated C kinase 1 [Source:MGI Symbol;Acc:MGI:101849]
Snord2-201	small nucleolar RNA, C/D box 2 [Source:MGI Symbol;Acc:MGI:3819528]

Table 9: Top 15 expressed transcripts and smallRNAs based on tags-per-million-normalized counts.

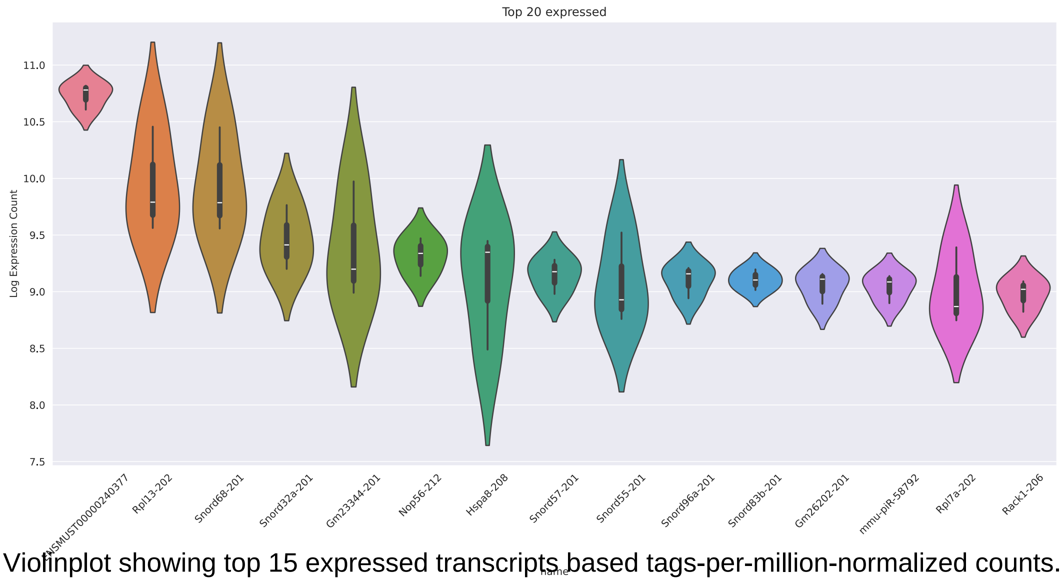

Figure 9: Violinplot showing top 15 expressed transcripts based tags-per-million-normalized counts.

Astrocytes_KIN vs. Astrocytes_WT

Name	log2 FoldChange	Pvalue	Padj
Mus_musculus_tRNA-Thr-AGT-2-1	1.14	0.0	0.0
Mus_musculus_tRNA-Thr-AGT-1-2	1.1	0.0	0.0
Mus_musculus_tRNA-Thr-TGT-2-1	1.07	0.0	0.0
Mir181a-1hg-201	1.07	0.0	0.0
Gm12895-201	1.01	0.0	0.0
Mus_musculus_tRNA-Thr-AGT-1-1	1.13	0.0	0.0
Gm12896-201	1.19	0.0	0.0
Mus_musculus_tRNA-Glu-TTC-3-2	1.16	0.0	0.0
Mus_musculus_tRNA-Thr-AGT-1-3	1.02	0.0	0.0
Mus_musculus_tRNA-Ala-AGC-10-1	1.03	0.0	0.0
Mus_musculus_tRNA-Glu-TTC-3-1	1.25	0.0	0.0
mmu-miR-181b-5p	1.05	0.0	0.0
Mir181b-2-201	1.1	0.0	0.0
mmu-miR-92a-3p	1.35	0.0	0.0
Mir335-201	2.16	0.0	0.0
Mir181b-1-201	1.08	0.0	0.0
Mus_musculus_tRNA-Thr-AGT-3-1	1.36	0.0	0.0
Mus_musculus_tRNA-Ala-TGC-6-1	1.16	0.0	0.0
Anks1b-230	1.17	0.0	0.0
Mir93-201	1.01	0.0	0.0

Table 10: Top differentially upregulated transcripts and smallRNAs in Astrocytes_KIN.

Name	log2 FoldChange	Pvalue	Padj
Slc1a2-202	-1.55	0.0	0.0
Sparcl1-201	-1.56	0.0	0.0
Atp1a2-201	-1.94	0.0	0.0
Scd2-201	-1.16	0.0	0.0
Slc6a11-201	-2.51	0.0	0.0
Malat1-204	-1.16	0.0	0.0
ENSMUST00000239709	-1.0	0.0	0.01
Glul-201	-1.51	0.0	0.0
Atp1b2-202	-1.8	0.0	0.0
mmu-piR-67239	-1.14	0.0	0.0
Gpr37l1-201	-1.12	0.0	0.01
Atp1b2-201	-1.83	0.0	0.0
mmu-piR-68892	-1.24	0.0	0.0
Cst3-201	-1.36	0.0	0.0
Atp1a2-202	-1.74	0.0	0.0
ENSMUST00000240382	-1.07	0.0	0.02
Bc1-201	-1.31	0.0	0.0
Slc4a4-207	-1.57	0.0	0.0
ENSMUST00000239851	-1.02	0.0	0.02
Atp1a2-203	-2.16	0.0	0.0

Table 11: Top differentially downregulated transcripts and smallRNAs in Astrocytes_KIN.

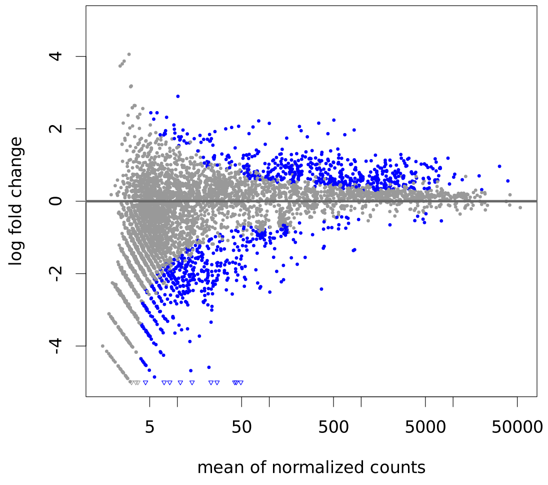

Figure 10: MA-plot showing the log2 fold changes attributable to a given variable over the mean of normalized transcript counts for all the samples. Points will be colored blue if the adjusted p-value is less than 0.1..

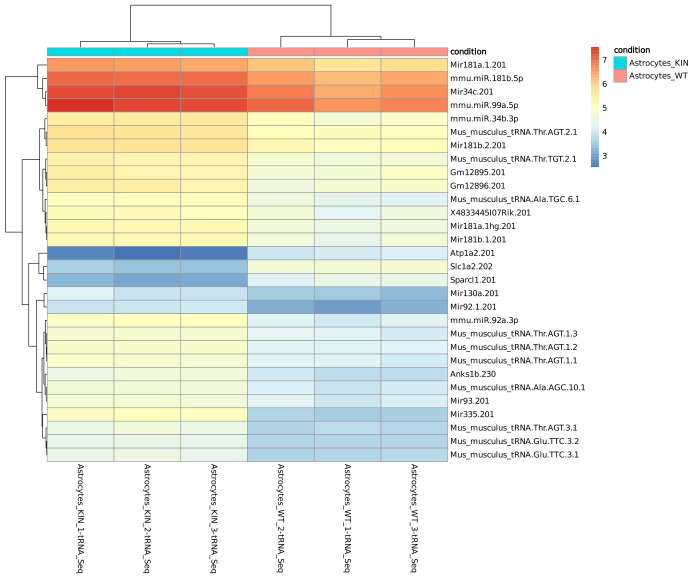

Figure 11: Clustered heatmap showing top differencially expressed transcripts and smallRNAs.

Microglia_KIN vs. Microglia_WT

Name	log2 FoldChange	Pvalue	Padj
mmu-miR-34a-5p	1.95	0.0	0.0
Mir34a-201	1.94	0.0	0.0
mmu-miR-146a-5p	1.17	0.0	0.0
Mir146-201	1.28	0.0	0.0
mmu-miR-107-3p	1.06	0.0	0.0
Mir3074-1-201	1.11	0.0	0.0
Tlcd2-203	1.27	0.0	0.0
mmu-miR-221-3p	1.62	0.0	0.0
mmu-miR-1839-5p	1.14	0.0	0.0
mmu-miR-22-3p	1.29	0.0	0.0
mmu-let-7i-5p	1.24	0.0	0.0
mmu-miR-324-5p	1.3	0.0	0.0
Mir221-201	1.67	0.0	0.0
Mir222-201	1.88	0.0	0.0
mmu-miR-17-3p	1.01	0.0	0.0
Aopep-205	1.07	0.0	0.0
Mir22hg-201	1.22	0.0	0.0
Mir22-201	1.23	0.0	0.0
Mir146b-201	1.7	0.0	0.0
Mir24-1-201	1.15	0.0	0.0

Table 12: Top differentially upregulated transcripts and smallRNAs in Microglia_KIN.

Name	log2 FoldChange	Pvalue	Padj
Gm23105-201	-1.01	0.0	0.0
Scarna8-201	-1.3	0.0	0.0
mmu-piR-67886	-1.16	0.0	0.0
mmu-piR-57926	-1.03	0.0	0.0
Gm31600-201	-1.11	0.0	0.0
mmu-piR-57305	-1.14	0.0	0.0
mmu-piR-67555	-1.06	0.0	0.01
Gm24362-201	-1.31	0.0	0.0
mmu-piR-68887	-1.14	0.0	0.01
mmu-piR-68030	-1.11	0.0	0.01
mmu-piR-68014	-1.3	0.0	0.0
mmu-piR-61509	-1.13	0.0	0.01
ENSMUST00020182858	-1.33	0.0	0.0
mmu-miR-124-3p	-1.24	0.0	0.01
Ccnb1ip1-202	-1.2	0.0	0.01
mmu-miR-9-5p	-1.19	0.0	0.01
Mir6240-201	-1.09	0.0	0.03
Lars2-205	-1.08	0.0	0.03
Ccdc107-206	-1.62	0.0	0.0
mmu-piR-67239	-1.52	0.0	0.0

Table 13: Top differentially downregulated transcripts and smallRNAs in Microglia_KIN.

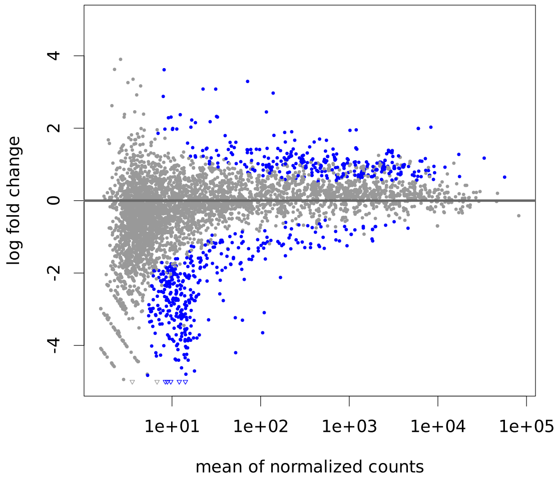

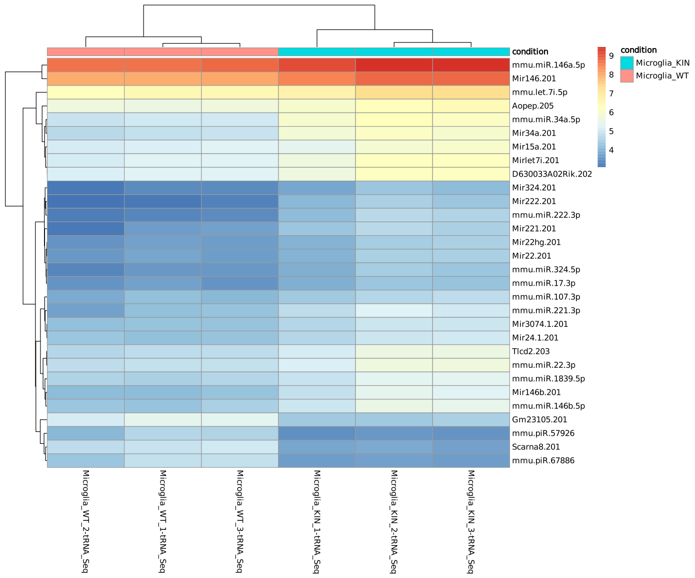

Figure 12: MA-plot showing the log2 fold changes attributable to a given variable over the mean of normalized transcript counts for all the samples. Points will be colored blue if the adjusted p-value is less than 0.1..

Figure 13: Clustered heatmap showing top differencially expressed transcripts and smallRNAs.

Neurons_KIN vs. Neurons_WT

Name	log2 FoldChange	Pvalue	Padj
ENSMUST00000243265	1.1	0.01	1.0
mmu-miR-92a-3p	1.07	0.01	1.0
Mir9-1-201	1.05	0.01	1.0
ENSMUST00000242216	1.19	0.01	0.92
Mir9-2-201	1.1	0.01	1.0
ENSMUST00000244522	1.08	0.01	1.0
ENSMUST00000246401	1.05	0.01	1.0
Mir181b-2-201	1.02	0.02	1.0
Mir92-1-201	1.11	0.01	1.0
mmu-miR-9-5p	1.22	0.01	1.0
mmu-piR-52917	1.16	0.03	1.0
mmu-miR-142a-3p	1.32	0.02	1.0
Gm24405-201	1.23	0.04	1.0
mmu-miR-9b-3p	1.59	0.01	1.0
Mus_musculus_tRNA-Tyr-GTA-1-1	1.54	0.03	1.0
Mir92-2-201	1.61	0.05	1.0
mmu-miR-17-3p	1.72	0.05	1.0
mmu-miR-29a-5p	2.45	0.02	1.0
Nasp-201	2.21	0.04	1.0
Myo9a-205	3.29	0.04	1.0

Table 14: Top differentially upregulated transcripts and smallRNAs in Neurons_KIN.

Name	log2 FoldChange	Pvalue	Padj
mmu-piR-44760	-1.17	0.0	0.82
Eva1c-207	-1.05	0.02	1.0
1700122C19Rik-203	-1.04	0.03	1.0
Dido1-201	-1.44	0.0	0.92
Gm19220-201	-1.35	0.01	1.0
mmu-miR-451a	-1.57	0.0	0.92
ENSMUST00000240057	-1.2	0.03	1.0
4930488L21Rik-201	-1.29	0.02	1.0
Hbb-bs-201	-1.57	0.01	1.0
Mir451b-201	-1.44	0.01	1.0
Sec61a1-208	-1.59	0.01	1.0
Gm12792-201	-1.44	0.02	1.0
mmu-miR-486a-5p	-1.64	0.02	1.0
mmu-miR-200b-3p	-2.09	0.0	0.69
Cltc-202	-1.67	0.02	1.0
Prkg2-203	-1.76	0.01	1.0
D030040B21Rik-201	-1.49	0.04	1.0
mmu-miR-200a-3p	-2.69	0.0	0.16
Mir200a-201	-2.69	0.0	0.17
ENSMUST00020183045	-2.52	0.0	0.33

Table 15: Top differentially downregulated transcripts and smallRNAs in Neurons_KIN.

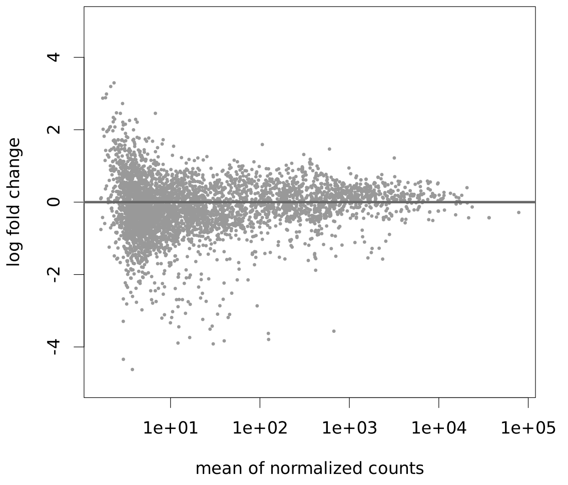

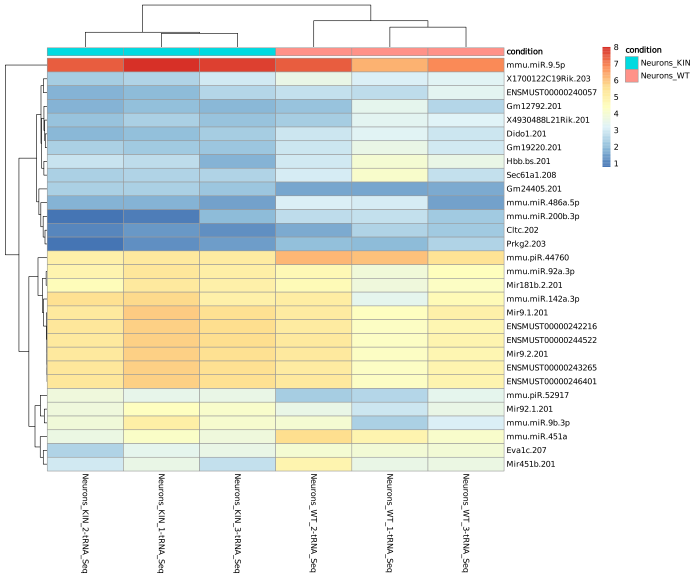

Figure 14: MA-plot showing the log2 fold changes attributable to a given variable over the mean of normalized transcript counts for all the samples. Points will be colored blue if the adjusted p-value is less than 0.1..

Figure 15: Clustered heatmap showing top differencially expressed transcripts and smallRNAs.

References

[1] Martin, M. (2011) Cutadapt Removes Adapter Sequences from High-Throughput Sequencing Reads. EMBnet Journal, 17, 10-12. https://doi.org/10.14806/ej.17.1.200
[2] https://www.bioinformatics.babraham.ac.uk/projects/fastqc/
[3] Langmead, B., & Salzberg, S. L. (2012). Fast gapped-read alignment with Bowtie 2. Nature methods, 9(4), 357-359. https://doi.org/10.1038/nmeth.1923
[4] Anders, S., Pyl, P. T., & Huber, W. (2015). HTSeq--a Python framework to work with high-throughput sequencing data. Bioinformatics (Oxford, England), 31(2), 166-169. https://doi.org/10.1093/bioinformatics/btu638
[5] Love, M. I., Huber, W., & Anders, S. (2014). Moderated estimation of fold change and dispersion for RNA-seq data with DESeq2. Genome biology, 15(12), 550. https://doi.org/10.1186/s13059-014-0550-8
[6] Stephens, M. (2017). False discovery rates: a new deal. Biostatistics, 18(2), 275-294.
[7] Ewels, P., Magnusson, M., Lundin, S., & Käller, M. (2016). MultiQC: summarize analysis results for multiple tools and samples in a single report. Bioinformatics (Oxford, England), 32(19), 3047-3048. https://doi.org/10.1093/bioinformatics/btw354
