# Supplementary figures and images for: Astro-Versus Microglia-Enriched Transcriptomes from Aged Atxn2-CAG100-Knockin Mice Suggest Underlying Pathology of RNA Processing at Ribosomes, and Possibly at U-Bodies
Source: Cells. 2026 Apr 15;15(8):699. doi: 10.3390/cells15080699 (PMC13115098; doi:10.3390/cells15080699)

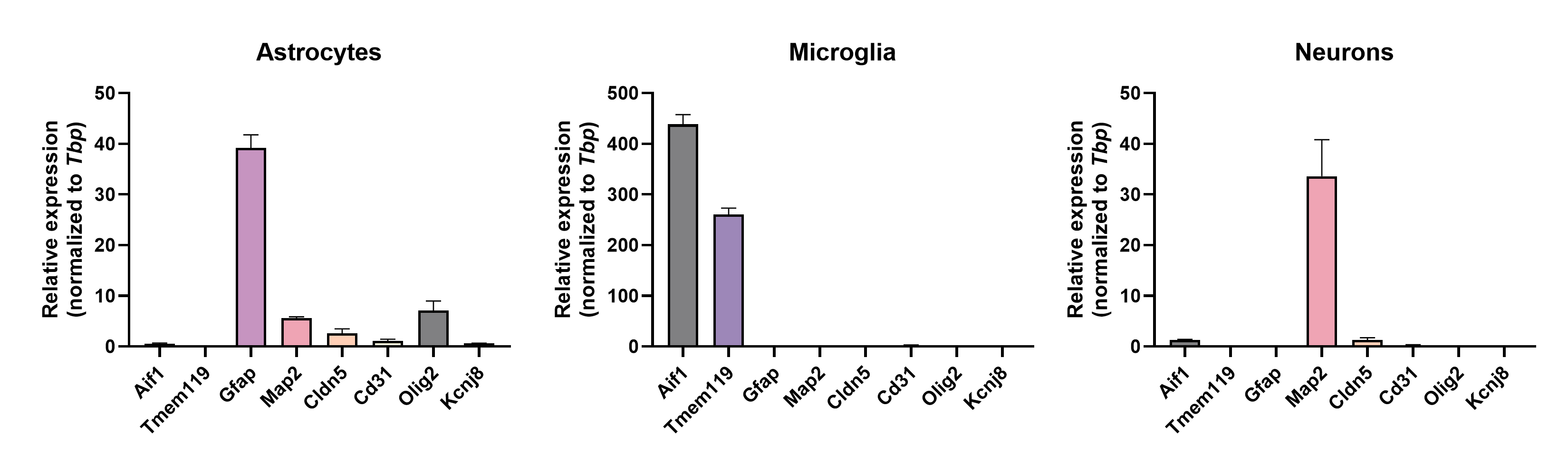

Supplement: Supplementary file 1 [file cells-15-00699-s001.zip › AuburgerKandi_SupplFigS1.tif]
